# Compositional Data Analysis of Periodontal Disease Microbial Communities

**DOI:** 10.3389/fmicb.2021.617949

**Published:** 2021-05-17

**Authors:** Laura Sisk-Hackworth, Adrian Ortiz-Velez, Micheal B. Reed, Scott T. Kelley

**Affiliations:** ^1^Department of Biology, San Diego State University, San Diego, CA, United States; ^2^Department of Nanoengineering, Joint School of Nanoscience and Nanoengineering, North Carolina Agricultural and Technical State University, Greensboro, NC, United States

**Keywords:** periodontal disease, CLR, compositional data analysis, microbiome, oral microbiome, C-reactive protein

## Abstract

Periodontal disease (PD) is a chronic, progressive polymicrobial disease that induces a strong host immune response. Culture-independent methods, such as next-generation sequencing (NGS) of bacteria 16S amplicon and shotgun metagenomic libraries, have greatly expanded our understanding of PD biodiversity, identified novel PD microbial associations, and shown that PD biodiversity increases with pocket depth. NGS studies have also found PD communities to be highly host-specific in terms of both biodiversity and the response of microbial communities to periodontal treatment. As with most microbiome work, the majority of PD microbiome studies use standard data normalization procedures that do not account for the compositional nature of NGS microbiome data. Here, we apply recently developed compositional data analysis (CoDA) approaches and software tools to reanalyze multiomics (16S, metagenomics, and metabolomics) data generated from previously published periodontal disease studies. CoDA methods, such as centered log-ratio (clr) transformation, compensate for the compositional nature of these data, which can not only remove spurious correlations but also allows for the identification of novel associations between microbial features and disease conditions. We validated many of the studies’ original findings, but also identified new features associated with periodontal disease, including the genera *Schwartzia* and *Aerococcus* and the cytokine C-reactive protein (CRP). Furthermore, our network analysis revealed a lower connectivity among taxa in deeper periodontal pockets, potentially indicative of a more “random” microbiome. Our findings illustrate the utility of CoDA techniques in multiomics compositional data analysis of the oral microbiome.

## Introduction

Periodontal disease (PD) manifests as bacterial biofilms (plaque) that lead to gum inflammation, recession, and, in later stages, degradation of the bone and tooth loss. Despite the prevalence of the disease, which affects over 45% of United States adults, the precise role of the oral microbiome in the progression of PD remains elusive (Eke et al., 2012). Prior to the development of next-generation sequencing (NGS) technologies, a cluster of three species deemed the “red complex,” consisting of *Porphyromonas gingivalis*, *Treponema denticola*, and *Tannerella forsythia*, was found to be associated with the PD clinical factors gum pocket depth and bleeding (Socransky et al., 1998). While some individual species of the oral microbiome contributing to PD, such as the members of the red complex, have been studied extensively (Lamont and Jenkinson, 1998), the presence of these specific species is not enough to explain the occurrence of PD (Ximénez-Fyvie et al., 2000). NGS technologies have revealed greater diversity of the oral microbiome and a complex relationship between microbiome composition and periodontal disease states, including an association between increasing microbial diversity and pocket depth (Kroes et al., 1999; Paster et al., 2001; Faveri et al., 2008; Griffen et al., 2012). Analysis of periodontal disease metagenomes has also revealed a novel bacterium strongly associated with the red complex and periodontal disease (Torres et al., 2019). Furthermore, high inter-patient diversity of the oral microbiome complicates deciphering the relationship between periodontal treatments or changes in disease state on the associated microbiome (Kumar et al., 2006; Schwarzberg et al., 2014; Califf et al., 2017).

While NGS technologies illuminate a great deal of information about the oral microbiome, most microbiome analyses ignore the compositional structure of NGS microbiome data, which presents problems in statistical and biological interpretation. Microbiome data are compositional for two main reasons. First, sequencing only captures a proportion of the microbes in a sample, so the counts of taxa in each sample are relative rather than absolute. As the measurement of one taxon increases, the measurement of another taxon must decrease regardless of whether its absolute abundance is actually lower. Second, as the count total obtained in a run of NGS sequencing is capped by sequencing depth limitations, each sample size is different, rendering the counts of taxa between samples incomparable. Common normalization methods for microbiome data, such as rarefaction, transcripts per million, and library size normalizations, attempt to make samples with different library sizes comparable, but generate proportional data still constrained by its relative nature (Gloor et al., 2017). Metabolomics, another data type that is commonly used in conjunction with microbiome analysis, is also relative in nature and therefore compositional. Furthermore, the integration of multiomics data, or different “omics” datasets like proteomics, metabolomics, and metagenomics, from the same sample is challenging due to the different scales with which these data are measured.

Most statistical tests assume that the sample data exist in real space, where Euclidian geometry and distance formulas can be used to describe the distance between points. However, compositional data exist in a space known as the simplex where dimensions are arbitrary and values are subject to spurious correlations (Aitchison, 1982; Gloor et al., 2017). Compositional data analysis (CoDA) approaches have been developed to deal with these constraints of compositional data. One method gaining traction is the centered log-ratio (clr) transformation, which recasts relative count data with respect to the sample’s geometric mean and creates scale-invariant data in Euclidian space where the use of multivariate statistical methods is valid (Gloor et al., 2017; Quinn et al., 2018). We recently showed that analyzing clr-transformed compositional datasets can reveal novel relationships, allow better discrimination between variables, and facilitate the integration of multiomics 16S, internal transcribed spacer (ITS), and metabolomic datasets (Sisk-Hackworth and Kelley, 2020).

In this work, we applied CoDA approaches, namely, clr transformation prior to standard methods such as non-metric multidimensional scaling (NMDS) ordination, Spearman’s correlation, multiomics structure correlation, beta dispersion, random forest, and network analysis, as well as the log-ratio balance method used in the R package selbal (Rivera-Pinto et al., 2018), to 16S, metagenomic, cytokine, and metabolomic datasets from prior studies of patients with periodontal disease before and after treatments. By reanalyzing these data with a CoDA approach, we integrated these multiomics datasets to reveal patterns and correlations between the disease state, microbes, metabolites, and cytokines, in addition to the relationships between community structure and disease state not identified with standard normalization methods.

## Materials and Methods

### Study Descriptions

This study incorporated data from two separate studies. The standard periodontal treatment (PT) study consisted of patients with periodontal disease and investigated the biofilms of periodontal pockets through 16S sequences, metagenomic sequences, and serum cytokine levels before and after standard periodontal treatments (Schwarzberg et al., 2014; Delange et al., 2018; Vijay Kumar et al., 2018). A total of 21 males and 38 females with an average age of 29 years were recruited from an American Indian/Alaska Native population in Southern California for the PT study. Eight patients had mild periodontitis (pocket depth less than 3 mm), 40 had moderate periodontitis (3–6 mm), and 11 had severe periodontitis (pocket depth over 6 mm). The second study measured pocket metabolites, 16S sequences, metagenomic sequences, and the serum cytokine levels of patients before and after treatments with 0.25% sodium hypochlorite (SHT) (Califf et al., 2017). For this study, 19 males and 15 females with an average age of 41 years were recruited among patients of the Ostrow School of Dentistry at the University of Southern California. In the SHT study, periodontal pocket depths ranged from 3 to 12 mm, while pocket depths in the PT ranged from 1.3 to 3.8 mm. The disease classes for the SHT study were separated into class “A” (pocket depth up to 6 mm), class “B” (pocket depth between 6 and 8 mm), and class “C” (pocket depth over 8 mm). Further details on the patient populations can be found in Schwarzberg et al. (2014) and Delange et al. (2018) for the PT study and in Califf et al. (2017) for the SHT study.

### PT Study Data

The original PT data contained 76 samples of 247 16S operational taxonomic units (OTUs), 144 samples of six cytokine inflammatory markers, and 23 samples of 3,830 bacterial metagenomic OTUs. The 16S ribosomal RNA (rRNA) sequences and the mapping file from in this study are accessible at: http://dx.doi.org/10.6084/m9.figshare.855613 and http://dx.doi.org/10.6084/m9.figshare.855612.

The serum cytokine data, raw reads from the 16S rRNA sequences, and metagenomic OTUs, classified by Kraken, were published previously (Delange et al., 2018; Vijay Kumar et al., 2018; Torres et al., 2019). Details on the study population, sampling, disease classification, and cytokine identification can be found in previously published papers (Schwarzberg et al., 2014; Delange et al., 2018; Vijay Kumar et al., 2018; Torres et al., 2019).

### SHT Study Data

The SHT study contained 286 samples of 773 16S OTUs, 215 samples of 914 tandem mass spectrometry (MS/MS) features, and 24 samples of 3,770 bacterial metagenomic features. The 16S rRNA sequences used in this study were accessed through the European Nucleotide Archive under project PRJEB19122 (Califf et al., 2017). Metabolite data from tandem mass spectrometry were downloaded from the online MassIVE repository of the GNPS database under MassIVE ID number MSV000078894. Metagenomic sequence libraries, generated from 24 subgingival samples from the SHT study patients and classified *via* Kraken, were obtained from Dr. Pedro Torres (Torres et al., 2019).

### 16S Sequence Analysis

16S sequencing data were analyzed using QIIME 2020.2 (Bolyen et al., 2019). Sequences were clustered into 100% identity using v-search OTU clustering (Rognes et al., 2016). Taxonomy was assigned to sequences using the RDP Classifier (Wang et al., 2007) retrained on Greengenes 13_5 (McDonald et al., 2012) *via* QIIME 2.

### Data Reduction and Transformation

**FIGURE 1:**
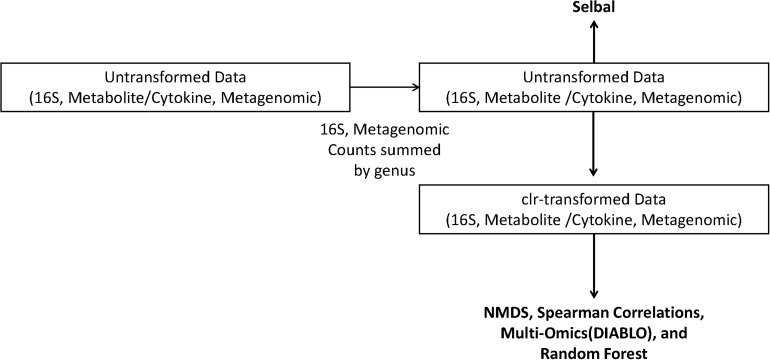
Schematic of the analyses performed on the centered log-ratio (clr)-transformed periodontal treatment (PT) and sodium hypochlorite (SHT) data. Only untransformed data were inputted into selbal, and clr-transformed data were used to calculate Spearman’s correlations, non-metric multidimensional scaling (NMDS), and DIABLO analyses.

Due to computational constraints, the numbers of features in the original sequencing and metabolomic datasets were reduced for *selbal* analysis (see below). The same reduced datasets were then used for the rest of the analyses. Genera of the 16S bacterial taxa present in greater than 10% of the samples, the 181 most abundant metagenomic taxa counts, and the 65 most abundant metabolites were selected for correlation analysis. For both PT and SHT studies, samples with a NA value for pocket depth in the mapping file were removed from all analyses. For the PT data, only samples with an overall response of improved or worsened were kept for all the analyses, determined by whether pocket depth decreased or increased, respectively (Schwarzberg et al., 2014). For the SHT data, only subgingival samples with disease class “A” or “C” were used on all analyses, as class “B” contained too few samples. Disease status was classified by maximum pocket depth (“A” = up to 6 mm, “B” = 6–8 mm, and “C” = over 8 mm) (Califf et al., 2017). For both the PT and SHT studies, OTUs were summed by genus for each sample in each of the 16S and metagenomic datasets. Zero replacement was performed with the pseudo-counts method from the R package zCompositions (Palarea-Albaladejo and Martín-Fernández, 2015) version 1.3.3. clr transformation was performed separately on all datasets (not on combined “multiomics” datasets). The clr transformation was computed for each sample *j*: each feature in that sample was divided by the geometric mean of all the feature counts in the sample, then the natural log of that ratio was taken (Aitchison, 1982).

$$\text{clr}\,\left(X_{j}\right)=\left[\ln\left(\frac{X_{1\,j}}{g\,\left(X_{j}\right)}\right),\cdots ,\ln\,\left(\frac{X_{D\,j}}{g\,\left(X_{j}\right)}\right)\right]$$

where *X*_*j*_ is the list of features in a sample, *g*(*X*_*j*_) is the geometric mean of the features in sample *X*_j_, *X*_1_*_j_* is the first feature in a sample, and *X*_Dj_ is the last feature in a sample of *D* values. To guide the reader, we have provided a diagram of the various datasets and analyses used in this study (Figure 1).

### NMDS Ordination Plots

clr-transformed values were used to generate the NMDS ordination plots with the R package vegan version 2.5.6 using Euclidean distances (Oksanen et al., 2019). The NMDS plots were created in R using ggplot2 version 3.2.1c (Wickham, 2016) and the samples were colored and shaped by periodontal treatment, overall response, and disease class. Permutational multivariate analysis of variance (PERMANOVA) was performed for each condition (periodontal treatment, overall response, and disease class) in every dataset (16S bacteria, cytokine, metabolites, and bacterial metagenomics) using the R package vegan with 9,999 permutations, with the *p* values corrected for multiple comparisons using the Benjamini–Hochberg method. The multivariate PERMANOVA test determines whether the centroid of a sample set is equal among the specified categories (e.g., periodontal treatment and disease class). The centroid was estimated using the between-sample Euclidean distances.

### Beta Dispersion

Beta dispersion, which measures the distance of each sample in a category from the centroid of that category, was estimated with the R package vegan using between-sample Euclidian distances. The beta dispersion test is a multivariate test used to determine whether the dispersion of samples is equivalent among categories. The *p* values were adjusted using the Benjamini–Hochberg method.

### Spearman’s Correlations

Spearman’s correlations were computed using the R package psych v1.0.67 (Revelle, 2020). For each periodontal treatment, overall response, and disease class, we computed the correlations between genera from four different combined multiomics datasets: (1) 16S bacteria and cytokine (PT); (2) 16S bacteria, cytokine, and bacterial metagenomics (PT); (3) bacteria and metabolite (SHT); and (4) bacteria, metabolite, and bacterial metagenomics (SHT). The *p* values were adjusted with the Bonferroni correction.

### Multiomics Integration

We integrated the same datasets as in the Spearman’s correlations using the DIABLO framework, a method for multiomics classification and integration, in the mixOmics R package version 6.10.8c (Rohart et al., 2017). We assessed the correlation structure at the component level for each of the three conditions on their respective dataset: periodontal treatment, overall response, and disease class.

### Microbial Balances

We identified differentially abundant taxa, metabolites, and cytokines using the R package selbal version 0.1, a compositional data analysis method that detects microbial signatures between different sample types by identifying the smallest number of differentially abundant taxa that is predictive of sample condition. Raw measurements of cytokines, metabolites, 16S, and metagenomic taxa summed by genus were inputted to selbal, as it performs zero handling and transformation within the package. Although selbal was designed with microbial balances in mind, the method is valid for finding balances of other data types, such as metabolite and cytokine data. Furthermore, selbal only finds balances for dichotomous and continuous response variables, so we performed this analysis only for the variables periodontal treatment (dichotomous), disease class (dichotomous), and pocket depth (continuous).

### Random Forest

A random forest classifier was implemented in Python using the scikit-learn package (Pedregosa et al., 2012) to identify cytokines, metabolites, and 16S genera that discriminate between pocket depth (PT) and disease class (SHT). The metrics used to analyze the random forest classifier include accuracy, out-of-bag (OOB) score, mean accuracy, and area under the curve of the receiver of components (AUC-ROC). For the PT data, the pocket depth boundary used to distinguish high and low pocket depths was 2.6 mm. For the SHT study, only disease classes “A” and “C” were used as disease class “B” had few samples.

### Network Analysis

Using the R package psych, we calculated Pearson’s correlations for each of the following datasets: 16S OTU, combined 16S OTU–cytokine for the PT study, and 16S for the SHT study. Correlations with a magnitude of | 0.55| or greater were kept; all other values were changed to zero. Using the psych package, *p* values were calculated for each pairwise Pearson’s correlation. The correlation matrix and the *p* value matrix were then filtered to contain only significant correlations (those with a Bonferroni-corrected *p* value below 0.05). The resulting adjacency matrix was transformed into an igraph object using a function from the SpiecEasi library (Kurtz et al., 2015). Using igraph v.1.2.5 package (Csardi and Nepusz, 2006), a network was constructed from the adjacency matrix using the OTUs as nodes and the Pearson’s correlation values as edge weights. Networks were constructed with nodes scaled according to the eigen centrality.

For each network, we calculated the number of nodes, edges, as well as the diameter and transitivity. Nodes represent individual genera or cytokines and edges are lines representing relationships between genera or cytokines. The diameter of a network is the shortest distance between the furthest apart nodes in a network. Transitivity, ranging from 0 to 1, measures the average connectedness of a network, with higher values signifying that a high proportion of nodes are connected to surrounding nodes, which indicates the presence of tightly connected clusters of nodes. To identify taxa that occupy important structures of the network, the R package igraph was used to calculate the eigenvector centrality (eigen centrality) and betweenness centrality. Eigen centrality identifies which highly connected nodes are connected to other highly connected nodes; these highly connected nodes therefore form most of the architecture that orders the network. Betweenness centrality represents the frequency that a node is traversed when the shortest paths in a network are calculated; high betweenness centrality indicates nodes that facilitate correlations between other nodes.

## Results

### Beta Diversity

**FIGURE 2:**
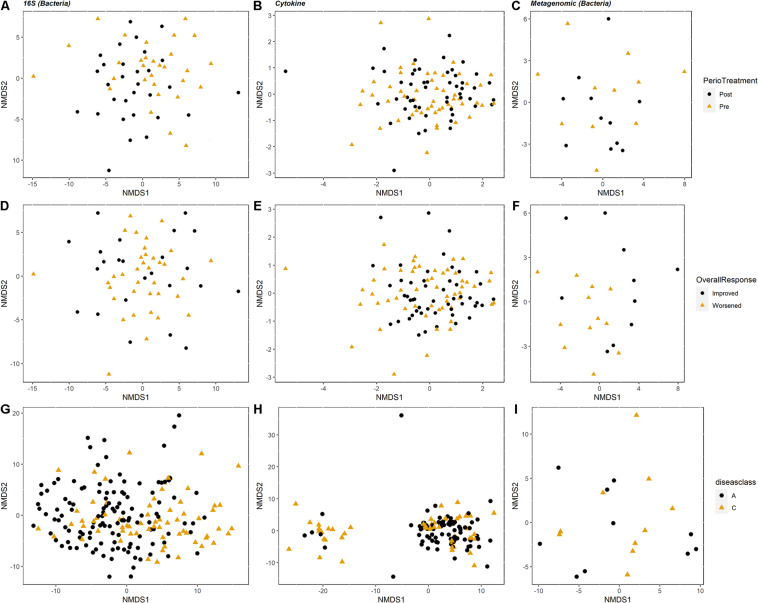
Non-metric multidimensional scaling (NMDS) ordination plots showing clustering of the samples. For the periodontal treatment (PT) study **(A–F)**, *columns* correspond to dataset type: 16S, cytokines, and metagenomics are columns 1, 2, and 3, respectively (*n* = 60, 104, and 22). The *top row* **(A–C)** is colored by periodontal treatment and the *second row* **(D–F)** colored by overall response. For the sodium hypochlorite (SHT) study **(G–I)**, *columns* correspond to dataset type: 16S, metabolomics, and metagenomics are columns 1, 2, and 3, respectively (*n* = 209, 153, and 24). Plots are colored by disease class.

We used NMDS ordination to determine the clustering of samples by condition (periodontal treatment, overall response, disease class, and pocket depth) for microbes, cytokines, and metabolites for the PT and SHT study datasets. For the 16S, cytokine, and metagenomic datasets from the PT study, we did not observe clustering of samples by periodontal treatment or overall response (Figure 2) or pocket depth (Supplementary Figure 1). The most distinct separation was seen in the metabolites for disease class in the SHT study, where most of the samples in disease class “A” clustered together and the samples in class “C” split into two groups (Figure 2H); a similar pattern was observed in the metabolites for pocket depth (Supplementary Figure 2). PERMANOVA indicated a difference between periodontal treatment groups for the 16S and cytokine datasets in the PT study (*p* −*adj* = 0.0234 for both comparisons; Table 1) and for the 16S and metabolite datasets in the SHT study (*p* −adj = 0.0.0012 and 0.006, respectively; Table 1). Analysis of beta dispersion showed no differences between the periodontal treatment or overall response groups in the PT study or by disease class in the SHT class (Supplementary Figure 3).

**TABLE 1:** PERMANOVA results (9,999 permutations) for centered log-ratio (clr)-transformed periodontal treatment (PT) 16S, cytokine, and metagenomic datasets and sodium hypochlorite (SHT) 16S, metabolite, and metagenomic datasets.

Dataset	Variable	*R*^2^	*p*	*p* −*adj*^a^
**PT study**				
16S	Periodontal treatment	0.0343	0.0053	0.0234
	Overall response	0.0129	0.7848	0.7848
Cytokine	Periodontal treatment	0.0343	0.0078	0.0234
	Overall response	0.0175	0.1152	0.1683
Metagenomic	Periodontal treatment	0.0687	0.1403	0.1683
	Overall response	0.0972	0.0293	0.0586
**SHT study**				
16S	Disease class	0.032	0.0004	0.001
Metabolite	Disease class	0.028	0.0040	0.006
Metagenomic	Disease class	0.044	0.5482	0.548

*^*a*^Benjamini–Hochberg corrected for multiple comparisons.*

### Spearman’s Correlations

clr transformation reduces spurious correlations in compositional data, such as microbiome and metabolome data, and allows the application of statistical methods such as Spearman’s correlation (Quinn and Erb, 2019). We applied Spearman’s correlation to analyze the relationships between the multiomics datasets from both the PT and SHT studies. Most correlations between the combined multiomics datasets were within the same datasets (e.g., bacteria to bacteria), while few between-omics correlations (e.g., bacteria to cytokines) were detected. For the PT datasets, no significant (*p* < 0.05) bacteria–cytokine correlations were observed, except in the posttreatment samples that had worsened. In these samples, *Prevotella* was strongly correlated (*R*^2^ = 0.808) with interleukin (IL)-1 (Supplementary Figure 4).

In the SHT study, we observed many significant correlations among bacteria and metabolites when the datasets for samples of disease classes “A” and “C” were combined; *Acinetobacter*, *Rubrivivax*, and *Treponema* were positively correlated with six metabolites, while *Desulfovibrio*, *Paludibacter*, *Peptococcus*, *TG5*, and *Treponema* were negatively correlated with six different metabolites (Supplementary Figure 5A). In samples that were only disease class “A,” *Olsenella* and *Atopobium* were positively correlated with two metabolites, while *Treponema* was negatively correlated with one metabolite (Supplementary Figure 5B). No bacterial–metabolite correlations were observed in samples that were only disease class “C.”

### Multiomics Integration

**FIGURE 3:**
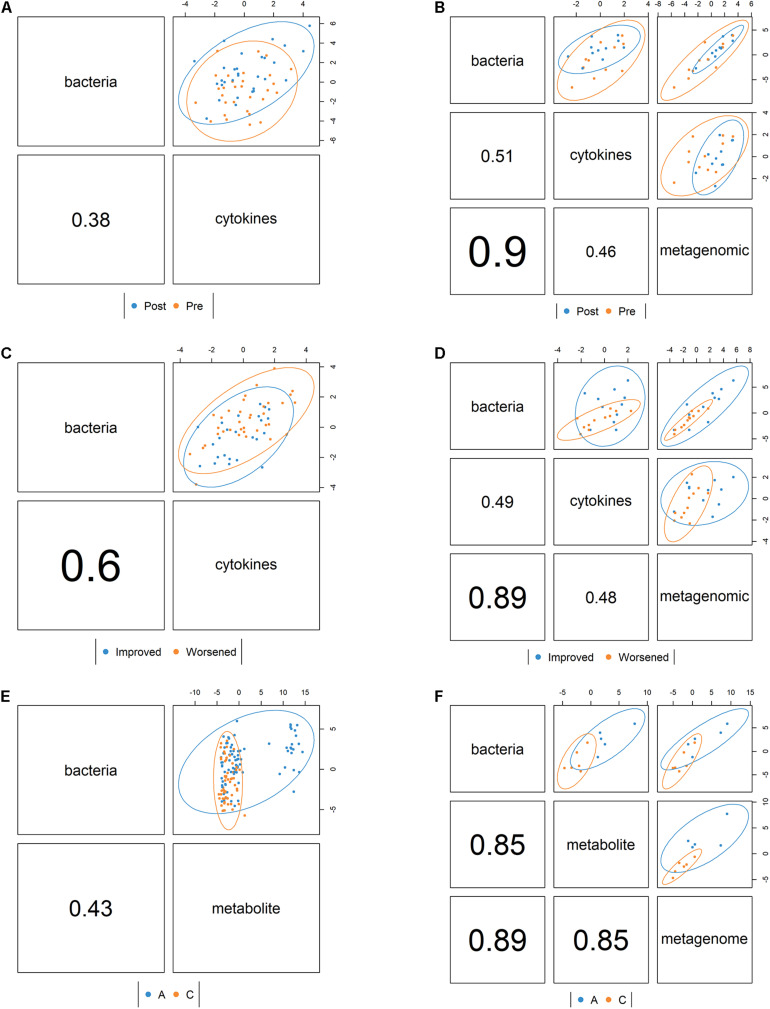
Correlation structure between datasets as determined by the mixOmics DIABLO framework colored by pre- *vs*. posttreatment for the periodontal treatment (PT) study **(A)** bacterial 16S and cytokine datasets and **(B)** bacterial 16S, cytokine, and metagenomic datasets and also colored by whether the disease improved or worsened for **(C)** the bacterial 16S and cytokine datasets and **(D)** the bacterial 16S, cytokine, and metagenomic datasets. For the sodium hypochlorite (SHT) study, samples were colored by disease class for **(E)** the bacterial 16S and metabolic datasets and **(F)** the bacterial 16S, metabolites, and metagenomic datasets. Values indicate the between-dataset correlation structure. *Ellipses* indicate discrimination by the multiomics components between samples by condition.

Using DIABLO, we found that the correlation structure between the 16S and cytokine datasets in the PT study was better when the overall response (improved or worsened) variable was included than when the time (pre *vs*. post) variable was incorporated (Figures 3A,C). When metagenomic data were included in the multiomics correlation (excluding samples that did not get the metagenome sequenced), the correlation structure did not change dramatically, but there was greater discrimination of samples by overall response (Figures 3B,D). For the SHT study, the metabolite and 16S combined datasets strongly distinguished between disease class, but the correlation structure was low (Figure 3E). When metagenomic data were included in the multiomics correlation (excluding samples that did not get the metagenome sequenced), the correlation structure greatly increased, but this may be an artifact of the severely reduced sample size (Figure 3F).

**FIGURE 4:**
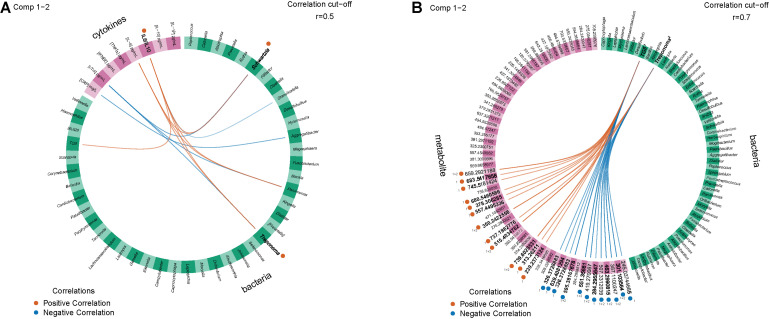
Circos plots show 16S and other features with inter-omics links indicating positive or negative correlations with an *R*^2^ greater than 0.5 between **(A)** the periodontal treatment (PT) study for the improved and worsened samples with the strongest correlation (0.6) for IL-6/IL-10 positively correlated with *Schwartzia* and *Treponema* and **(B)** the sodium hypochlorite (SHT) study for correlations with an *R*^2^ greater than 0.7 for disease classes “A” and “C.” *Bold labels* were the strongest correlations.

We generated Circos plots showing the correlations between the “omics” datasets using the DIABLO correlation structure. In the PT study, IL-6/IL-10 were strongly negatively correlated with *Treponema* and *Schwartzia* (Figure 4A). In the SHT study, TG5 and *Treponema* were strongly correlated with many unidentified metabolites (Figure 4B).

### Microbial Balances

Using selbal, we analyzed the differentially abundant genera, cytokines, and metabolites between different conditions. In the PT study, selbal identified many bacterial genera predictive of pretreatment samples, *Desulfobulbus*, *Bulleidia*, and *Hylemonella*, while *Abiotrophia* and *Haemophilus* were more predictive of posttreatment samples (Table 2 and Supplementary Figure 6A). For cytokines, selbal identified IL-6 as the most predictive of pretreatment samples and IL-6/IL-10 as the most predictive of posttreatment samples (Table 2 and Supplementary Figure 5B). From the metagenomic dataset, selbal identified that *Prevotella* predicted pretreatment and *Haemophilus* predicted posttreatment samples (Table 2 and Supplementary Figure 5C). In distinguishing overall response among the datasets, selbal identified *Desulfobulbus*, IL-6, and *Burkholderia* as more predictive in samples that improved and *Peptococcus*, C-reactive protein (CRP), and *Coryebacterium* as more predictive in samples that worsened (Table 2 and Supplementary Figures 6D–F). We then analyzed pre- and posttreatment balances for samples that improved and samples that worsened. In pretreatment samples that ended up improving, *Campylobacter*, *Bulleidia*, *Treponema*, IL-10, and *Actinomyces* were more relatively abundant, while in improved samples posttreatment *Peptococcus*, IL-1I(^2), and *Rothia* were relatively abundant (Table 2 and Supplementary Figures 7A–C). In pretreatment samples that later worsened, *TG5*, CRP, and *Veillonella* were more relatively abundant, while in posttreatment samples that had worsened, *Fusobacterium*, IL-6/IL-10, and *Haemophilus* were more relatively abundant (Table 2 and Supplementary Figures 7D–F).

**TABLE 2:** Summary of the microbial and metabolic balances generated from the periodontal treatment (PT) datasets.

Dataset	Variable	Denominator	Numerator	AUC-ROC
16S	Periodontal treatment	*Desulfobulbus*, *Bulleidia*, *Hylemonella*	*Abiotrophia*, *Haemophilus*	0.85
	Overall response	*Desulfobulbus*	*Peptococcus*	0.76
	Periodontal treatment for improved	*Campylobacter*, *Bulleidia*, *Treponema*	*Haemophilus*	0.83
	Periodontal treatment for worsened	*TG5*	*Fusobacterium*	0.76
	Pocket depth for improved	*Selenomonas*	*Filifactor*	0.73
	Pocket depth for worsened	*Filifactor*	*Paludibacter*	0.23
Cytokine	Periodontal treatment	IL-6	IL-6/IL-10	0.60
	Overall response	IL-6	CRP	0.66
	Periodontal treatment for improved	IL-10	IL-1I(^2)	0.66
	Periodontal treatment for worsened	CRP	IL-6/IL-10	0.58
	Pocket depth for improved	IL-10	IL-1I(^2)	0.42
	Pocket depth for worsened	CRP	IL-6/IL-10	0.06
Metagenomic	Periodontal treatment	*Prevotella*	*Haemophilus*	0.86
	Overall response	*Burkholderia*	*Corynebacterium*	0.90
	Periodontal treatment for improved	*Actinomyces*	*Rothia*	1.00
	Periodontal treatment for worsened	*Veillonella*	*Haemophilus*	1.00
	Pocket depth for improved	*Selenomonas*	*Porphyromonas*	0.90
	Pocket depth for worsened	*Veillonella*	*Bacillus*	0.74

**AUC-ROC*, area under the curve of the receiver of components.*

We also used selbal to explore which features’ relative abundance changed with the sum of all pocket depths. Within the improved samples, selbal identified *Selenomonas* and IL-10 as more associated with shallow pocket depths and *Filifactor*, IL-1I(^2), and *Porphyromonas* as more associated with deeper pocket depths (Table 2 and Supplementary Figures 8A–C). Within the worsened samples, *Filifactor*, CRP, and *Veillonella* were associated with shallow pocket depths, while *Paludibacter*, IL-6/IL-10, and *Bacillus* were associated with deeper pocket depths (Table 2 and Supplementary Figures 8D–F).

We also used selbal to determine the metabolite and microbial balances in the SHT study 16S and metagenomic datasets for disease class and pocket depth. Both 16S and metagenomic microbial balances identified the genus *Tanerella* as more predictive for disease class “A” and *Fusobacterium* as more predictive of disease class “C” (Table 3 and Supplementary Figures 9A,C). Metabolite balances were clearly identified, but the specific metabolites in the balance remain unknown (Table 3 and Supplementary Figures 8B,E,H). In disease class “A” samples, selbal identified *Desulfobulbus* and *Rothia* as more predictive of shallower pocket depths and *SHD-231* and *Fusobacterium* as more predictive of deeper pocket depth (Table 3 and Supplementary Figures 9D,F). For the samples in disease class “C,” shallow pocket depth was more associated with *Lactobacillus* and *Parabacteroides*, while deeper pocket depth was more associated with *Desulfovibrio* and *Bacteroides* (Table 3 and Supplementary Figures 9G,I).

**TABLE 3:** Summary of the microbial and metabolic balances generated from the sodium hypochlorite (SHT) datasets.

Dataset	Variable	Denominator	Numerator	AUC-ROC
16S	Disease class	*Capnocytophaga*	*Porphyromonas*	0.77
	Pocket depth for class A	*Desulfobulbus*	*SHD-231*	0.04
	Pocket depth for class C	*Lactobacillus*	*Desulfovibrio*	0.208
Metabolite	Disease class	301.1035645	637.32011211, 554.3658664	0.74
	Pocket depth for class A	419.270067	284.2958512	0.127
	Pocket depth for class C	245.0374468	494.5759899	0.264
Metagenomic	Disease class	*Tannerella*	*Fusobacterium*	0.88
	Pocket depth for class A	*Bacillus*	*Rothia*, *Fusobacterium*	0.892
	Pocket depth for class C	*Parabacteroides*	*Bacteroides*	0.848

**AUC-ROC*, area under the curve of the receiver of components.*

### Random Forest

**FIGURE 5:**
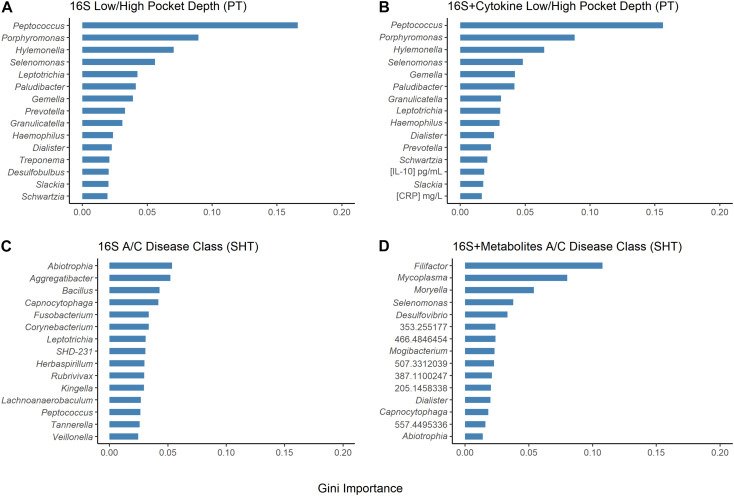
Results of random forest Gini importance bar plots. Periodontal treatment (PT) dataset high/low periodontal disease (PD) using **(A)** 16S and **(B)** 16S and cytokines. Sodium hypochlorite (SHT) dataset A/C disease classes **(C)** 16S and **(D)** 16S and metabolites. Larger Gini importance indicates that features can resolve more nodes in decision trees with more confidence.

The random forest machine learning classifier was trained to determine how accurately pocket depth class and disease class could be predicted from 16S bacteria OTUs and cytokines for the PT study or from 16S bacteria and metabolites for the SHT study. The most important cytokines were IL-10 and CRP (Figure 5B). The 16S features in the SHT study most predictive of disease class changed dramatically when metabolite data were included in the random forest analysis; the only feature recognized as highly important in both classifiers was *Abiotrophia* (Figures 5A–C). The addition of metabolite data to the 16S data increased the AUC-ROC scores compared with the 16S by itself in the SHT study (Table 4). However, the inclusion of the cytokine features did not improve the AUC-ROC scores in the PT study (Table 4).

**TABLE 4:** Results of random forest analysis with single and combined multiomics.

Comparisons	Accuracy	OOB score	Mean accuracy	AUC-ROC
**PT study**				
High *vs*. low (16S)	0.63	0.65	0.59	0.59
High *vs*. low (16S + cytokine)	0.63	0.57	0.59	0.59
**SHT study**				
A *vs*. C (16S)	0.72	0.69	0.73	0.6
A *vs*. C (16S + metabolite)	0.76	0.71	0.74	0.7

**OOB score*, out-of-bag score; *AUC-ROC*, area under the curve of the receiver of components.*

### Network Analysis

**FIGURE 6:**
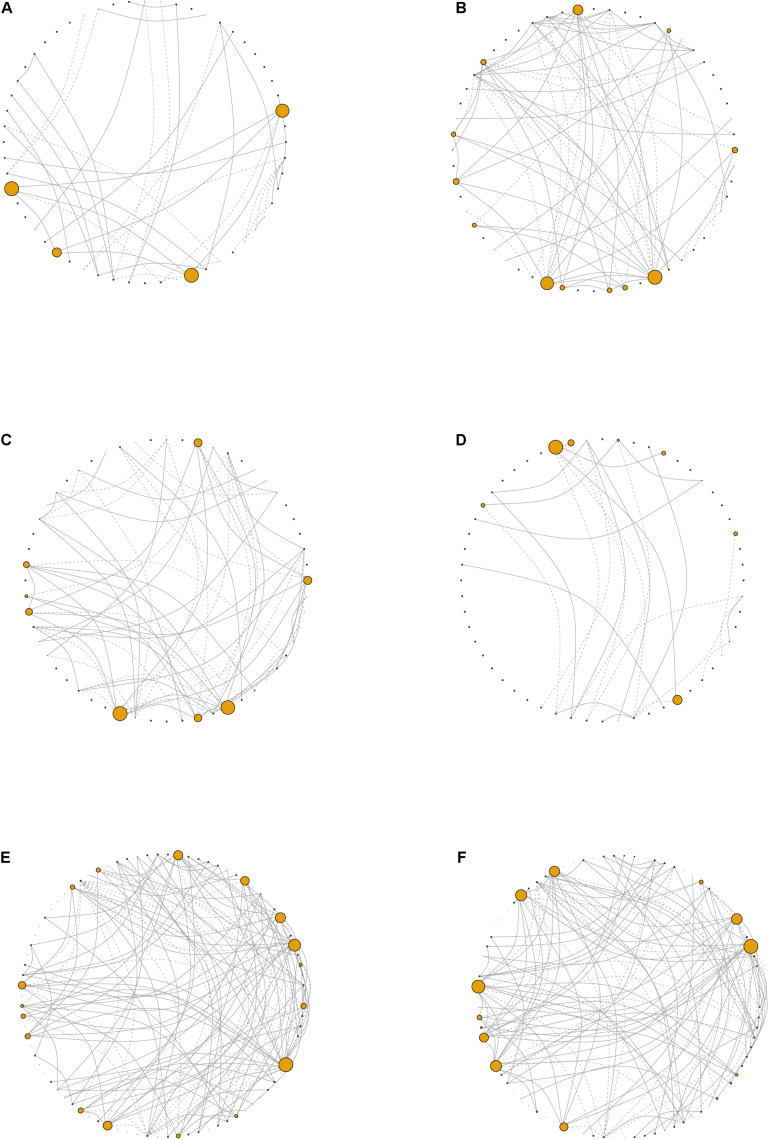
Network visualizations. Network plots derived from the periodontal treatment (PT) study patients whose oral status was designated as **(A)** gingivitis, **(B)** moderate, **(C)** shallow, or **(D)** deep. For the sodium hypochlorite (SHT) study, network plots for biofilms from patients with **(E)** class “A” or **(F)** class “C.” Node size corresponds to eigen centrality. *Dashed lines* represent negative correlations and *solid lines* represent positive correlations.

In the samples with moderate disease conditions, the Pearson’s correlation networks had approximately twice as many edges as those with gingivitis and a small diameter (Table 5 and Figures 6A,B). Transitivity was not different between disease conditions, implying that both networks have similar levels of inter-nodal interactions. In the gingivitis network, seven taxa formed correlations, of which four had eigen centralities that were greater than 0.1: *Pelomonas*, *Thermoanaerobacterium*, *Aeribacillus*, and *Ralstonia* (Figure 6A and Supplementary Table 1). The degree distribution of the gingivitis network did not strictly follow the characteristic shape of a power law distribution, but it did reveal high amounts of “low connectivity” and low amounts of “high connectivity,” while the moderate network had a degree distribution that followed the power law trend much more closely (Supplementary Figures 10A,B).

**TABLE 5:** Summary statistics of the networks generated from the periodontal treatment (PT) datasets comparing 16S and cytokines based on disease severity and pocket depth and the sodium hypochlorite (SHT) 16S datasets comparing oral microbiomes with different disease classes.

Data	Condition	Vertices	Edges	Diameter	Transitivity
16S	Gingivitis	48	32	10	0.49
	Moderate	48	69	6	0.45
16S + cytokine	Gingivitis	56	42	12	0.43
	Moderate	56	78	7	0.42
16S	Shallow	48	62	7	0.55
	Deep	48	22	6	0.20
16S + cytokine	Shallow	56	70	8	0.52
	Deep	56	26	6	0.18
16S	Class A	86	130	8	0.50
	Class C	86	124	8	0.46

Networks were also constructed for samples that had either shallow or deep pocket depths. Networks from the samples with shallow pockets had approximately three times as many edges as those with deep pockets (Table 5 and Figures 6C,D). The networks from the samples with shallower pockets had greater transitivity than those with deep pockets. No cytokines were highly connected (high eigen centralities) within any of the networks we constructed (Supplementary Tables 1–4), suggesting that cytokines were not strongly correlated with many taxa. The degree distribution for these networks followed the typical shape of a power law distribution (Supplementary Figures 10C,D).

For the SHT study, networks were constructed with 16S data for the samples from class A and class C disease status. The network structure characteristics were similar between the two networks describing the microbiomes of patients diagnosed with different disease classes (Table 4 and Figures 6E,F). The taxa with the highest eigen centralities in disease class “A” were *Streptobacillus*, *Aerococcus*, and *Arthrobacter*, while *Aerococcus*, *Massilia*, and *Gemella* had the highest eigen centralities in class “C” (Supplementary Tables 6,7). The degree distribution for these networks followed the typical shape of a power law distribution (Supplementary Figures 10E,F).

## Discussion

Our reanalysis, and expanded analysis, of previously published data from periodontal disease studies using CoDA techniques improved our ability to detect patterns and correlations in these data and provided new insights into the relationships of organisms, cytokines, and metabolites to the disease process. Analysis of beta-diversity using the clr-transformed datasets detected distinct clustering not observed in the original studies. In the SHT study, unlike the original PCoA analysis that saw no separation of samples by disease class for any “omics” dataset (Califf et al., 2017), we identified clustering of clr-transformed data by both disease class and pocket depth in the NMDS ordination plots, with disease class C and deeper pocket depth in the left cluster and disease class A and shallower pocket depth in the right cluster (Figure 2H and Supplementary Figure 2B). In the PT study, we did not see clustering of the samples by 16S, cytokine, or metagenomic datasets for any of the classifications (periodontal treatment, overall response, or pocket depth) in the NMDS ordination (Figure 2), which concurs with the findings from the original study (Schwarzberg et al., 2014). However, PERMANOVA showed differences between the pre-and posttreatment samples and disease class in the PT and SHT studies’ 16S data, findings not determined in the original studies due to the high level of individual variability (Tables 1–3).

The original PT study reported significant relationships between the combined abundance of *P. gingivalis*, *Fusobacterium nucleatum*, *T. forsythia*, and *T. denticola*, which are periodontal pathogens, and IL-1β (Vijay Kumar et al., 2018). However, the only significant correlation we observed in the PT study dataset was between IL-1 and *Prevotella*. IL-1 has been associated with periodontal disease severity (Offenbacher et al., 2007), and *Prevotella* includes species in the orange complex associated with periodontal disease (Socransky et al., 1998). For the SHT study, we observed significant bacterial–metabolite correlations among the diseased samples, which the original study did not investigate (Supplementary Figures 5A,B). Among the samples in all disease classes, *Paludibacter*, a bacterial genus associated with plaque in healthy patients (Chen et al., 2018), was negatively correlated with two metabolites (Supplementary Figure 5A). However, selbal identified this genus as more predictive of worsened samples with high pocket depth (Table 3 and Supplementary Figure 8D), so this genus may not be “health-associated” as previously thought. Other genera negatively correlated with metabolites in the samples of all disease classes were *Desulfovibrio*, *Peptococcus*, and *TG5* (Supplementary Figure 4A). *Desulfovibrio* species may stimulate the immune response (Dzierżewicz et al., 2010) and have been observed in periodontal pockets (Loubinoux et al., 2002). *Peptococcus* and *TG5* have been found in the oral microbiomes of periodontitis patients (Kumar et al., 2005; Apatzidou et al., 2017). *Acinetobacter*, which has been previously associated with periodontitis samples (Souto et al., 2014), and *Rubrivivax* were positively correlated with metabolites (Supplementary Figure 5A). As *Rubrivivax* is not commonly associated with periodontal disease, this genus may be a new route of study. *Treponema*, a “red complex” species, was correlated with most metabolites in the samples from all disease classes (Supplementary Figure 5A) and was correlated with one metabolite among the samples that were only within disease class “A” (Supplementary Figure 4B). Also significantly correlated with metabolites in disease class “A” samples were *Olsenella* and *Atopobium* (Supplementary Figure 5B). In periodontitis patients, *Olsenella* species have been detected in abundance (Chávez de Paz et al., 2004) and *Atopobium* species have been associated with periodontal disease (Kumar et al., 2005; Apatzidou et al., 2017).

The DIABLO multiomics integration uncovered an additional negative relationship between IL-6/IL-10 and *Treponema* and *Schwartzia* in the PT study and many correlations between *Treponema* and *TG5* with many metabolites in the SHT study (Figures 3A,B). *Schwartzia*, *Treponema*, and *TG5* have been associated with the biofilms of periodontal disease patients (Socransky et al., 1998; Camelo-Castillo et al., 2015; Apatzidou et al., 2017). Additionally, *T. denticola* has been shown to degrade IL-1β and IL-6 (Miyamoto et al., 2006), and infection of both *P. gingivalis* and *T. denticola* synergistically stimulated the production of IL-6 by macrophage-like cells (Tamai et al., 2009). Little is known about the association between *Schwartzia*, periodontal disease, and the immune response, so this genus is a potential new target for investigation. For the SHT study multiomics integration, disease class was able to distinguish integrated 16S and metabolomic samples (Figure 3E); disease class was also the only variable that distinguished between samples in our NMDS analysis, indicating that this discrimination may be largely due to metabolite differences.

The orange complex described by Socransky et al. (1998) as highly intercorrelated and associated with PD includes species from the genera *Prevotella* and *Fusobacterium*, which were both identified in our selbal analysis of microbial balances for the PT and SHT datasets. *Prevotella* relative abundance predicted pretreatment samples in the PT dataset and *Fusobacterium* relative abundance predicted high pocket depth and posttreatment samples that had worsened (Table 2 and Supplementary Figures 6C, 7D). *Fusobacterium* was also predictive of disease class “C” in the SHT study (Table 3 and Supplementary Figure 9C). This aligns well with the original PT studies, where *Fusobacterium* was significantly correlated with pocket depth and a decrease in *Prevotella* after treatment was associated with improvement (Schwarzberg et al., 2014; Califf et al., 2017). Some *Desulfobulbus* species likely play a role in the development of periodontal disease (Camelo-Castillo et al., 2015; Cross et al., 2018), and selbal identified this genus as predictive of pretreatment samples, samples that improved, and shallow pocket depth (Table 2 and Supplementary Figures 6A,D, 9D). Selbal indicated that *Porphyromonas*, the genus that includes a red complex species, was more predictive of deeper pockets in the PT study and disease class “C” in the SHT study (Tables 2 and 3 and Supplementary Figures 8C, 9A) and was found in the original study as correlating with high pocket depth (Califf et al., 2017). The most commonly identified cytokine by selbal, IL-6, was found in the original study to be significantly associated with severe periodontitis (Delange et al., 2018), while we found that IL-6 levels were predictive of pretreatment samples and samples that improved (Table 2 and Supplementary Figures 6B,E). Additionally, selbal identified CRP as predictive of samples that worsened (Table 2 and Supplementary Figure 6E), which is in contrast to the previous study which did not find a strong association between CRP and periodontal disease status (Delange et al., 2018). CRP was also identified by random forest as one of the two top important cytokines for predicting pocket depth (Figure 5B). Random forest also identified *Abiotrophia*, species of which have been isolated from dental plaques (Mikkelsen et al., 2000), as the most stable genus in predicting disease class in the SHT study 16S data, while in the PT study data, *Peptococcus* and *Porphyromonas* were the most important genera in predicting pocket depth (Figure 5).

Analysis of correlation networks can provide insights into the complexity, stability, and function of a microbial community (Barberán et al., 2012). The most striking disparity in overall network connectivity occurred in the PT study. The network analysis found that, for the PT study, networks of 16S and cytokines for the samples with deep pockets had fewer edges and lower transitivity (Table 5). Fewer inter-nodal connections and a lower overall network connectedness indicate a lack of interdependence of taxa in deeper pockets. Multiple studies have shown that pocket depth is correlated with greater alpha diversity and more pathogenic taxa (Christersson et al., 1992; Stoltenberg et al., 1993; Takeshita et al., 2016). The early stages of periodontal development involve well-known interactions between bacterial species (Nyvad and Kilian, 1987; Diaz et al., 2006; Chalmers et al., 2008), but as biofilms develop and become increasingly anaerobic, more pathogenic species establish within the biofilm (Van Winkelhoff et al., 2002; Vieira Colombo et al., 2016). The fewer connections that we observed in deep pockets could reflect a more random, or less stable, biofilm in the later stages of disease. Additionally, the networks constructed from the samples with shallow pockets had greater transitivity (Table 5), which implies the presence of more inter-nodal interactions within the shallow pocket networks and may be indicative of niche filtering, where similarities rather than differences between species allow the persistence of species in an environment (Röttjers and Faust, 2018). Network analysis also revealed that *Aerococcus* had a high eigen centrality value in the networks for disease classes “A” and “C” (Supplementary Tables 5,6). Higher eigen centralities indicate that nodes are critical for network stability and may point toward keystone species (Bauer et al., 2010; Mandakovic et al., 2018). While *Aerococcus* species have been found in the biofilms of periodontitis patients (Voropaeva et al., 2008), but as little is known about the association between *Aerococcus* and periodontal disease, this may be an interesting future avenue of study.

We should note potential effects of the study population demographics on our data. Most participants from the PT study had an overweight or obese body mass index, and 37% were smokers, both of which could affect the microbiome composition and inflammation levels measured in this study. Additionally, the PT study participants were American Indian/Alaskan Native, on average over a decade younger, and had a higher prevalence of females (66 *vs*. 44%) than the SHT study participants, so the differences in the results between the two studies could be due to effects of ethnicity, aging, or sex on the oral microbiome and inflammation. Our use of CoDA techniques, which confirmed many of the prior studies’ results and uncovered new findings, shows how this approach is a valuable addition to the current methods of microbiome data analysis for investigating oral disease. We have shown how CoDA approaches are especially useful when integrating multiomics due to the scale-invariance that the clr transformation confers on datasets. The identification of CRP as predictive of pocket depth and samples that worsened is a new finding and an important area of further study. We also identified understudied genera potentially important in periodontal disease (*Schwartzia*, *Rubrivivax*, and *Aerococcus*). Furthermore, the ability of unknown metabolites to discriminate between samples in selbal analyses, and the associations we determined between metabolites and particular taxa, highlights the need to study these compounds in the context of periodontal disease.

## Data Availability Statement

The datasets analyzed for this study and code for all analyses can be found on zenodo: https://doi.org/10.5281/zenodo.4604009 further inquiries can be directed to the corresponding author/s.

## Ethics Statement

The studies involving human participants were reviewed and approved by USC Health Sciences Campus Institutional Review Board, the SDSU Institutional Review Board (IRB) and the IRB of the Southern California American Indian Health Clinic. The patients/participants provided their written informed consent to participate in this study.

## Author Contributions

AO-V and MR performed the computations and generated figures with guidance from LS-H and SK. LS-H wrote the initial draft of the manuscript. SK, AO-V, and MR edited the manuscript. All authors discussed the results and contributed to the final manuscript.

## Conflict of Interest

The authors declare that the research was conducted in the absence of any commercial or financial relationships that could be construed as a potential conflict of interest.

## Supplementary Material

Supplementary Figure 1 NMDS ordination plots showing clustering of PT samples by pocket depth. Columns correspond to dataset type; 16S, Cytokine, and Metagenomic datasets are columns one, two, and three, respectively (*n* = 60, 104, and 22).

Supplementary Figure 2 NMDS ordination plots showing clustering of SHT samples by pocket depth. Columns correspond to dataset type; 16S, Metabolites, and Metagenomic datasets are columns one, two, and three, respectively (*n* = 209, 153, and 24).

Supplementary Figure 3 Beta dispersion analysis by periodontal treatment, response to treatment, and disease class. For the PT study, distance from the centroid of beta dispersion is shown for samples pre- and post-treatment for **(A)** 16S bacterial, **(B)** metabolite, and **(C)** metagenomic datasets and for response to treatment for **(D)** 16S bacterial, **(E)** metabolite, and **(F)** metagenomic datasets. Larger distances indicate samples which are far from the group centroid, small distances indicate samples which are close to the group centroid. For the SHT study, distance from the centroid is shown for samples by disease class for **(G)** 16S bacterial, **(H)** metabolite, and **(I)** metagenomic datasets.

Supplementary Figure 4 Bacterial-cytokine correlation matrix for post-treatment, worsened samples. All spaces with color are p-adj <0.05. Blue indicates a positive correlation; Dark orange indicates a negative correlation.

Supplementary Figure 5 Correlation matrices between comparisons of different SHT datasets (bacterial 16S-metabolite and bacterial 16S-metagenomic). **(A)** Bacterial-metabolite correlations on a combined dataset from all disease classes; **(B)** Bacterial-metabolite correlations on disease class “A.” All spaces with color are p-adj <0.05. Blue indicates a positive correlation, Dark orange indicates a negative correlation.

Supplementary Figure 6 Microbial and cytokine balances for PT dataset at different conditions computed with selbal, where numerator genera are more relatively abundant than denominator genera for higher balance values. Balances are shown for samples before and after periodontal treatment for **(A)** bacterial 16S data, **(B)** cytokine, and **(C)** metagenomic data. Balances are shown for improved and worsened samples of **(D)** bacteria 16S data, **(E)** cytokine, and **(F)** metagenomic data.

Supplementary Figure 7 Microbial and cytokine balances for PT dataset for improved **(A–C)** and worsened **(D–F)** samples computed with selbal. Balances before and after treatment are shown for samples that improved for **(A)** bacterial 16S data **(B)** cytokine, and **(C)** metagenomic data. Balances before and after treatment are shown for samples that worsened for **(D)** bacterial 16S data, **(E)** cytokine, and **(F)** metagenomic data.

Supplementary Figure 8 Selbal-computed microbial and cytokine balances for PT dataset associated with the sum of all pocket depths. Balances for improved samples by pocket depth (response variable) are shown for **(A)** 16S bacterial, **(B)** cytokine, and **(C)** metagenomic datasets. Balances for worsened samples by pocket depth are shown for **(D)** 16S bacterial, **(E)** cytokine, and **(F)** metagenomic datasets.

Supplementary Figure 9 Selbal-computed balances for SHT dataset for microbial and metabolite balances by different characteristics. For samples with disease class either “A” or “C,” balances shown are for **(A)** 16S bacterial, **(B)** metabolite, and **(C)** metagenomic data. Balances for samples with disease class “A” associated with pocket depth sum are shown for **(D)** 16S bacterial, **(E)** metabolite, and **(F)** metagenomic datasets. Balances for disease class “C” samples associated with pocket depth sum are shown for **(G)** 16S bacterial, **(H)** metabolite, and **(I)** metagenomic datasets.

Supplementary Figure 10 Network degree distributions. For the PT study for networks with OTUs and cytokines, degree distribution of inferred network from patients who had **(A)** gingivitis or **(B)** moderate disease, **(C)**, shallow or **(D)** deep pockets. For the SHT study, degree distribution of inferred network from patients who had class (E) “A” or (F) “C” disease.
