# Supplementary figures and images for: Compositional Data Analysis of Periodontal Disease Microbial Communities
Source: Front Microbiol. 2021 May 17;12:617949. doi: 10.3389/fmicb.2021.617949 (PMC8165185; doi:10.3389/fmicb.2021.617949)

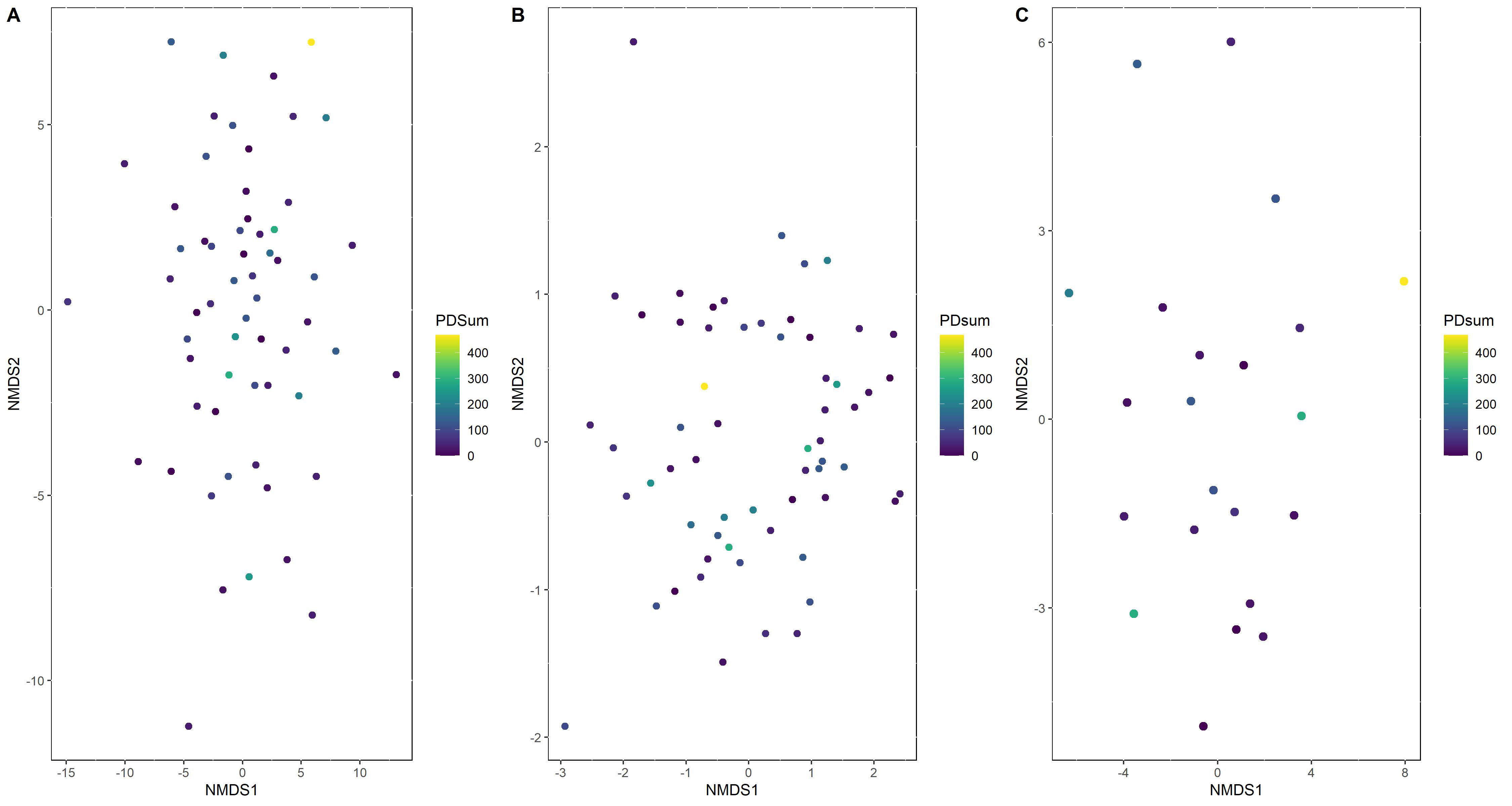

Supplement: Supplementary Figure 1 — NMDS ordination plots showing clustering of PT samples by pocket depth. Columns correspond to dataset type; 16S, Cytokine, and Metagenomic datasets are columns one, two, and three, respectively (n = 60, 104, and 22). [file Data_Sheet_2.ZIP › Supplemental Figures and Tables/Supplemental_Figure_1.tif]

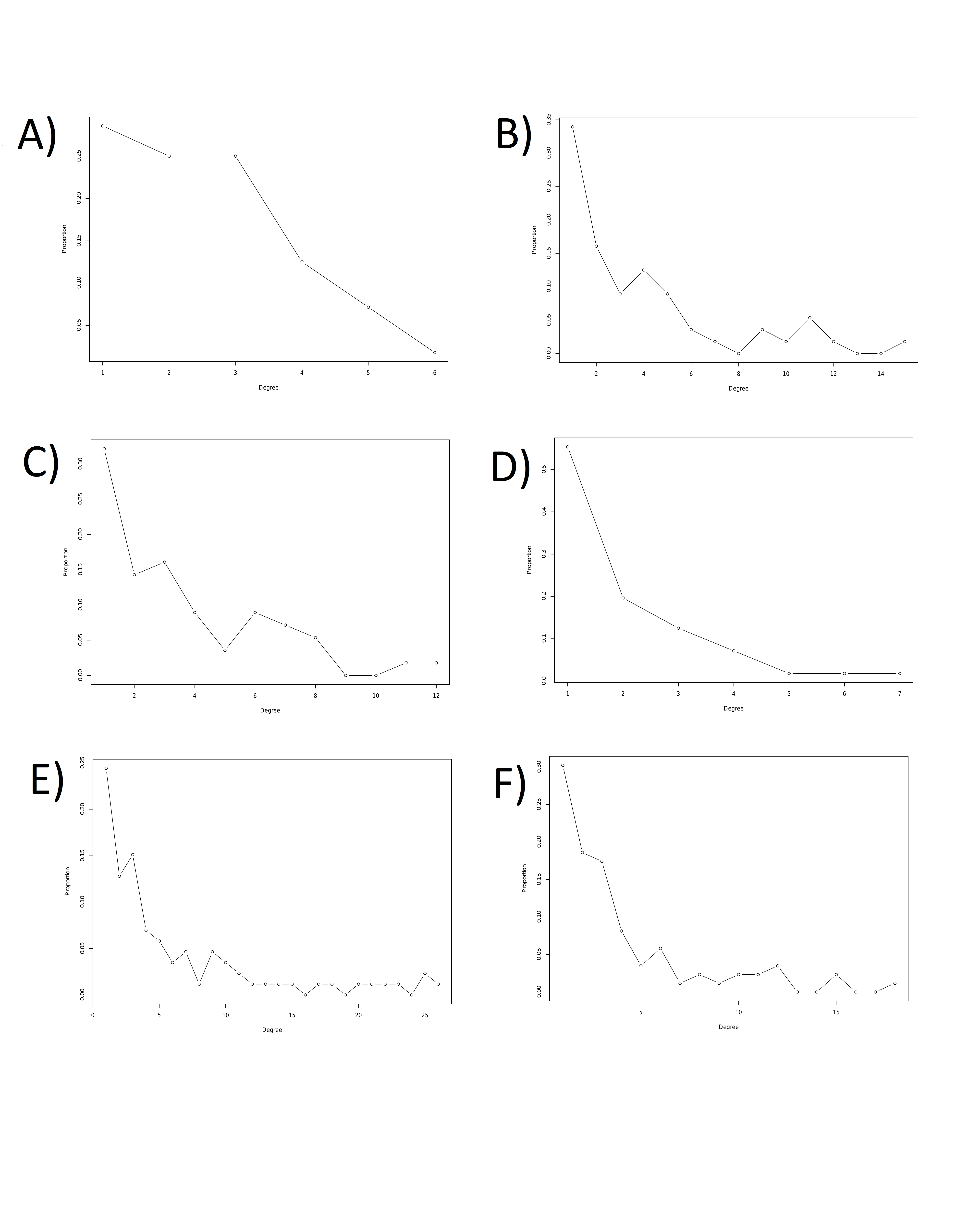

Supplement: Supplementary Figure 1 — NMDS ordination plots showing clustering of PT samples by pocket depth. Columns correspond to dataset type; 16S, Cytokine, and Metagenomic datasets are columns one, two, and three, respectively (n = 60, 104, and 22). [file Data_Sheet_2.ZIP › Supplemental Figures and Tables/Supplemental_Figure_10.png]

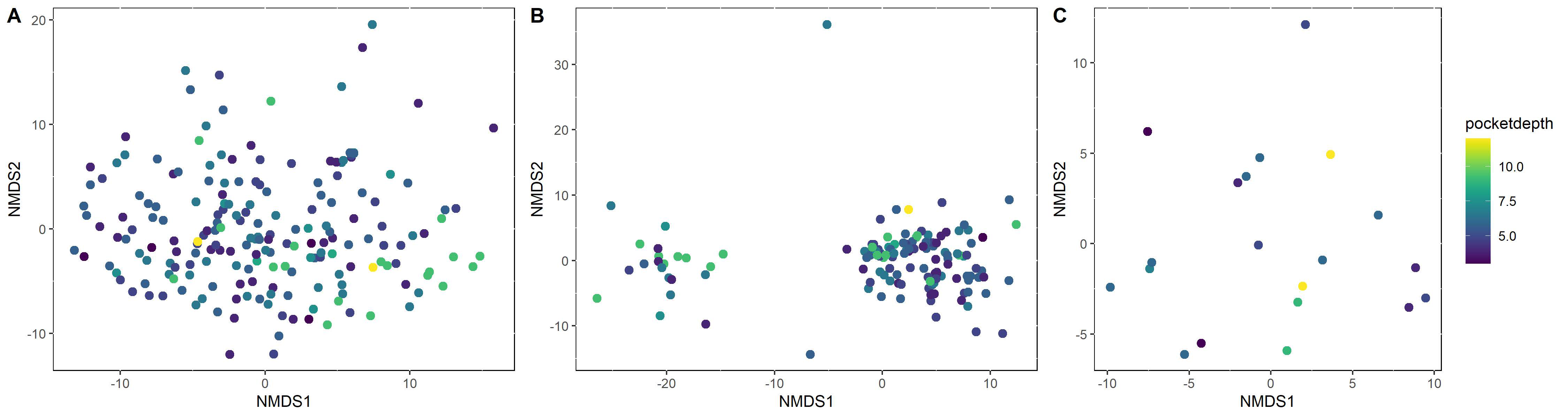

Supplement: Supplementary Figure 1 — NMDS ordination plots showing clustering of PT samples by pocket depth. Columns correspond to dataset type; 16S, Cytokine, and Metagenomic datasets are columns one, two, and three, respectively (n = 60, 104, and 22). [file Data_Sheet_2.ZIP › Supplemental Figures and Tables/Supplemental_Figure_2.tif]

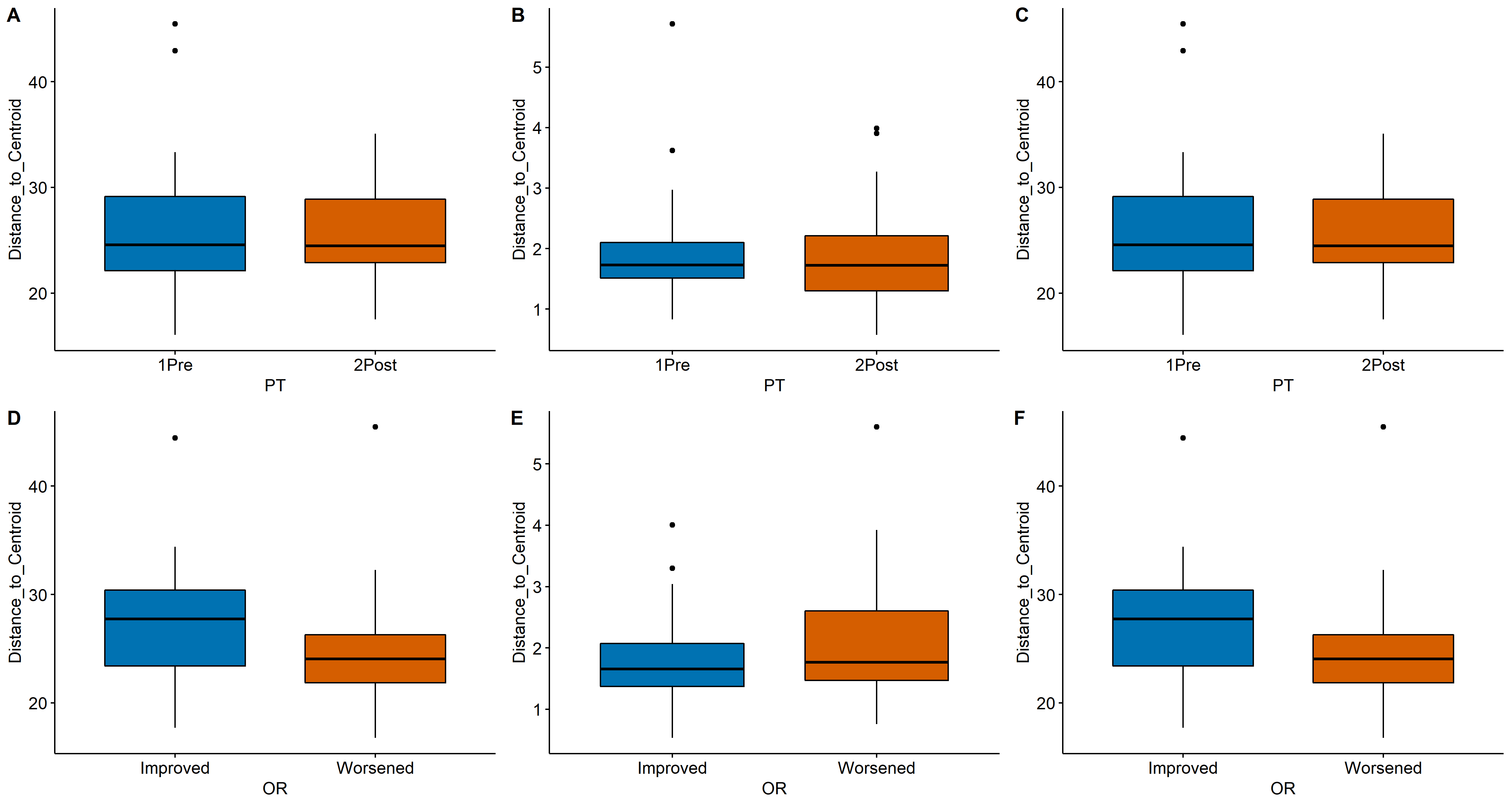

Supplement: Supplementary Figure 1 — NMDS ordination plots showing clustering of PT samples by pocket depth. Columns correspond to dataset type; 16S, Cytokine, and Metagenomic datasets are columns one, two, and three, respectively (n = 60, 104, and 22). [file Data_Sheet_2.ZIP › Supplemental Figures and Tables/Supplemental_Figure_3.tiff]

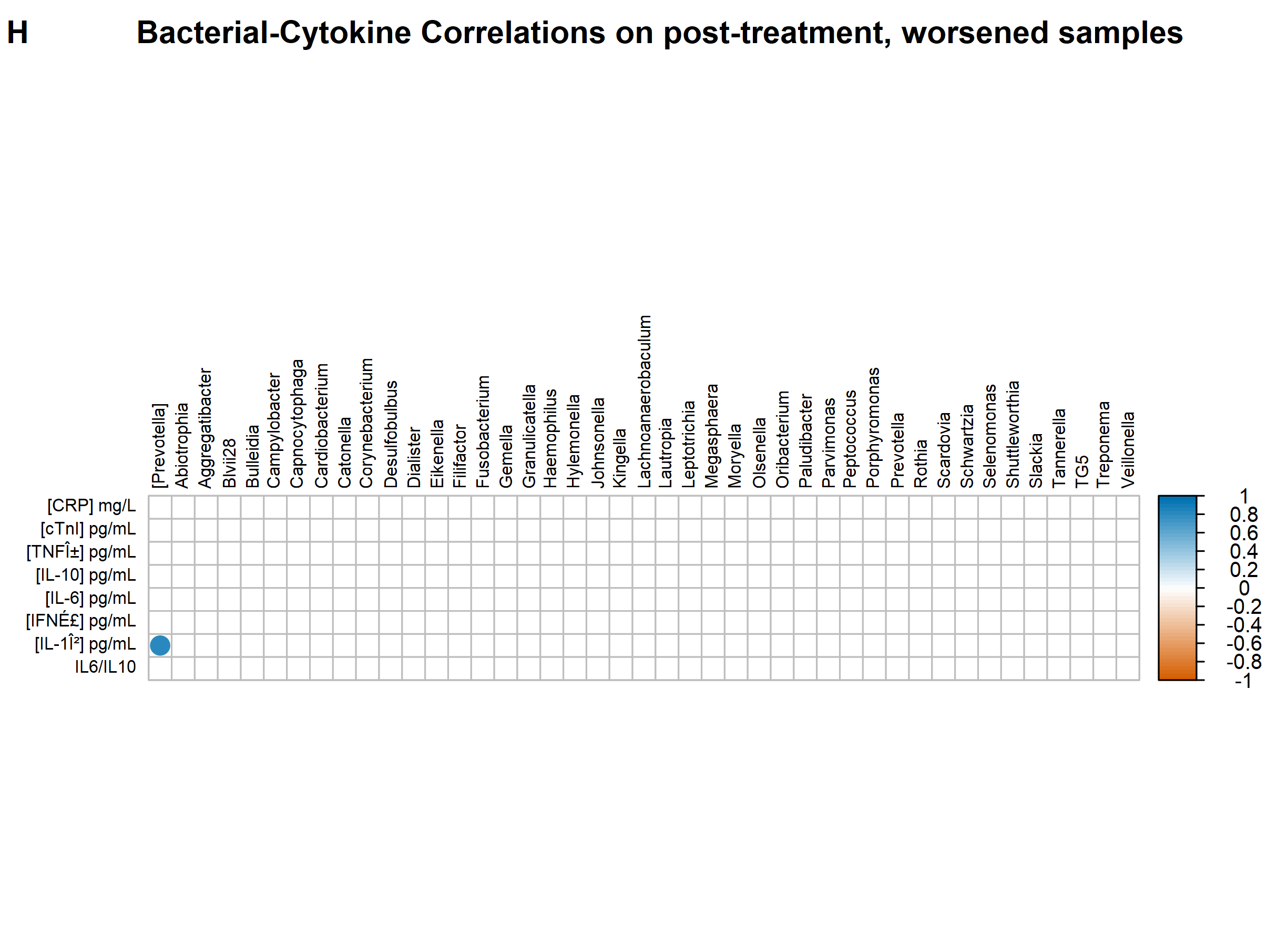

Supplement: Supplementary Figure 1 — NMDS ordination plots showing clustering of PT samples by pocket depth. Columns correspond to dataset type; 16S, Cytokine, and Metagenomic datasets are columns one, two, and three, respectively (n = 60, 104, and 22). [file Data_Sheet_2.ZIP › Supplemental Figures and Tables/Supplemental_Figure_4.tiff]

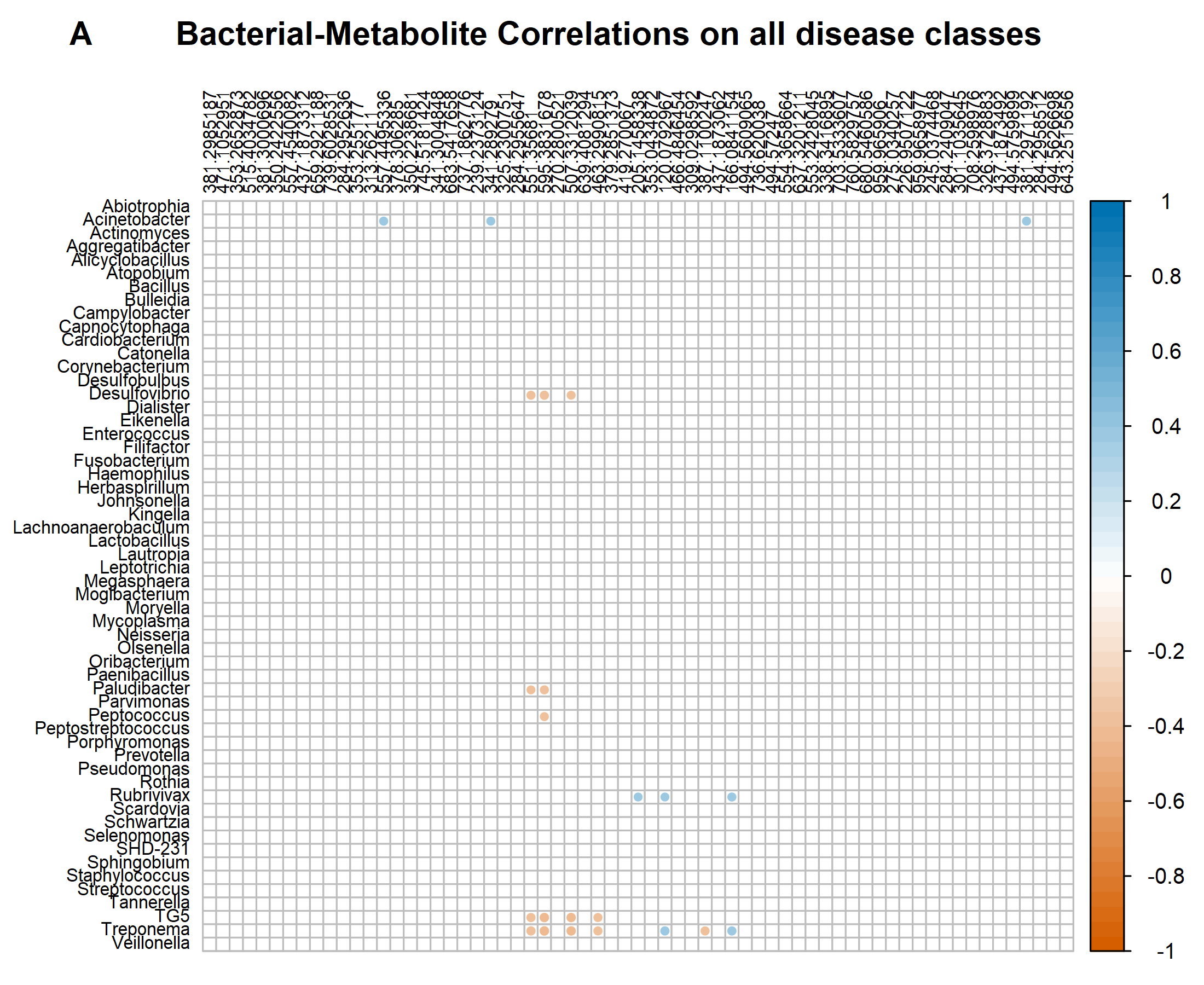

Supplement: Supplementary Figure 1 — NMDS ordination plots showing clustering of PT samples by pocket depth. Columns correspond to dataset type; 16S, Cytokine, and Metagenomic datasets are columns one, two, and three, respectively (n = 60, 104, and 22). [file Data_Sheet_2.ZIP › Supplemental Figures and Tables/Supplemental_Figure_5A.tiff]

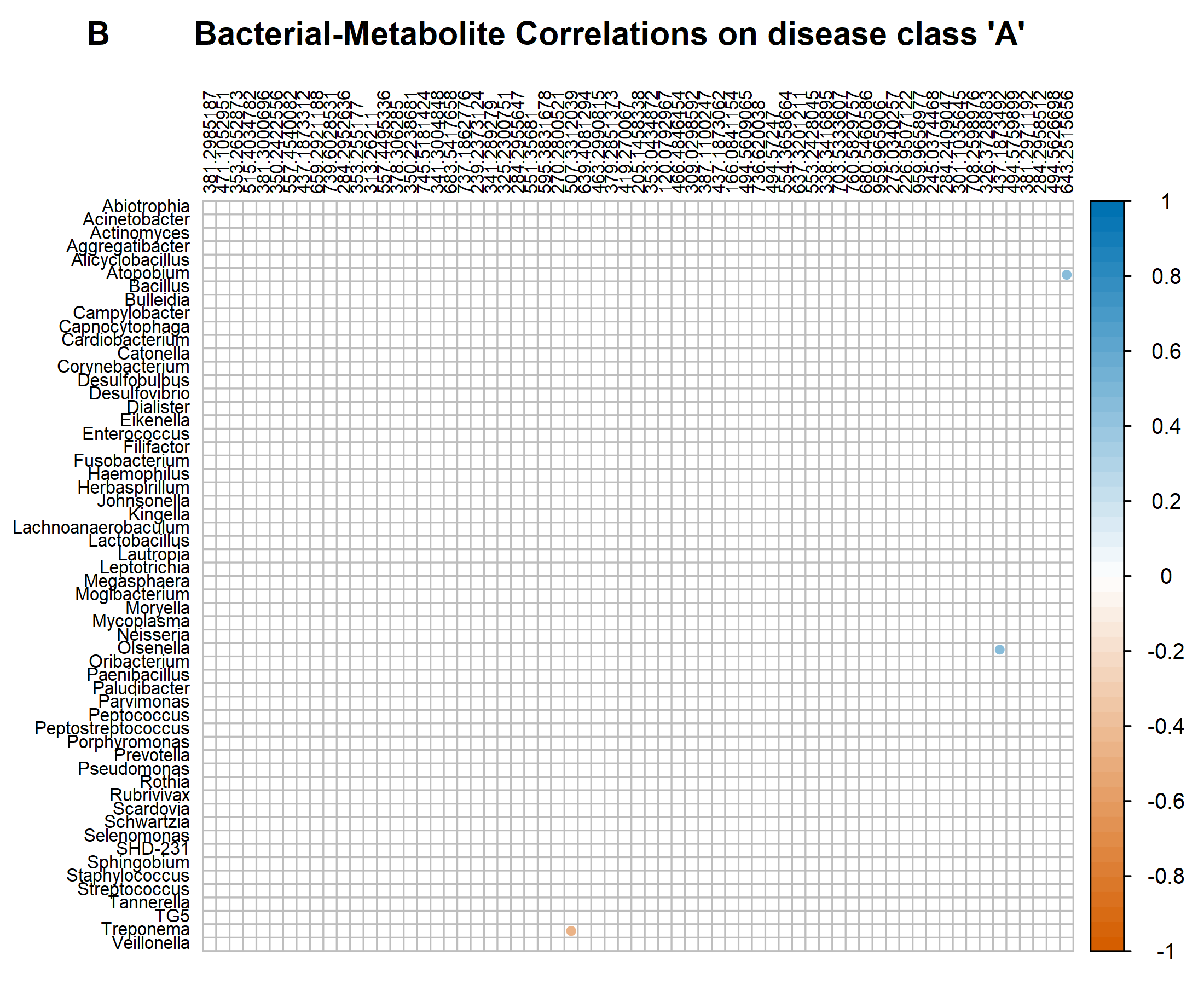

Supplement: Supplementary Figure 1 — NMDS ordination plots showing clustering of PT samples by pocket depth. Columns correspond to dataset type; 16S, Cytokine, and Metagenomic datasets are columns one, two, and three, respectively (n = 60, 104, and 22). [file Data_Sheet_2.ZIP › Supplemental Figures and Tables/Supplemental_Figure_5B.tiff]

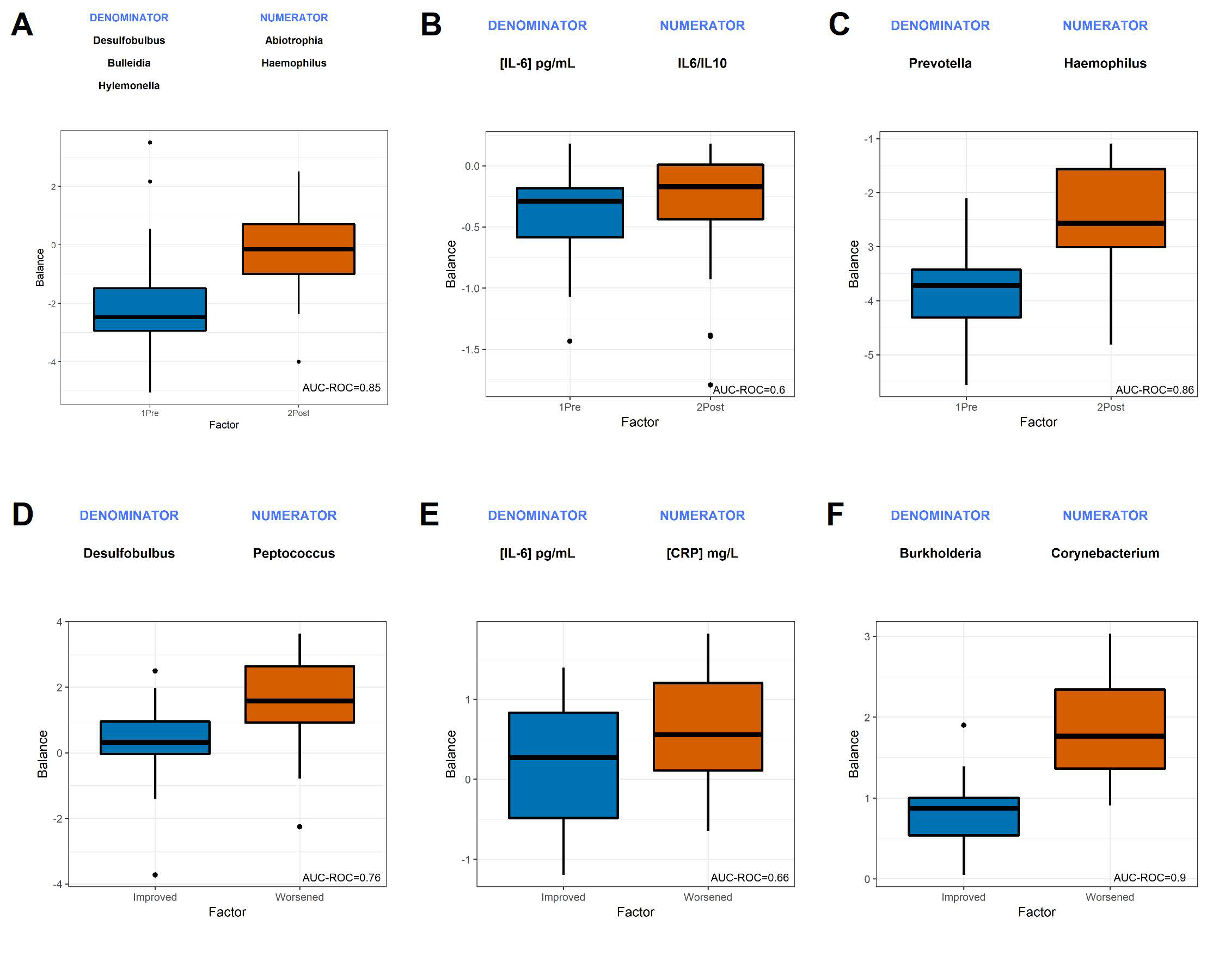

Supplement: Supplementary Figure 1 — NMDS ordination plots showing clustering of PT samples by pocket depth. Columns correspond to dataset type; 16S, Cytokine, and Metagenomic datasets are columns one, two, and three, respectively (n = 60, 104, and 22). [file Data_Sheet_2.ZIP › Supplemental Figures and Tables/Supplemental_Figure_6.tif]

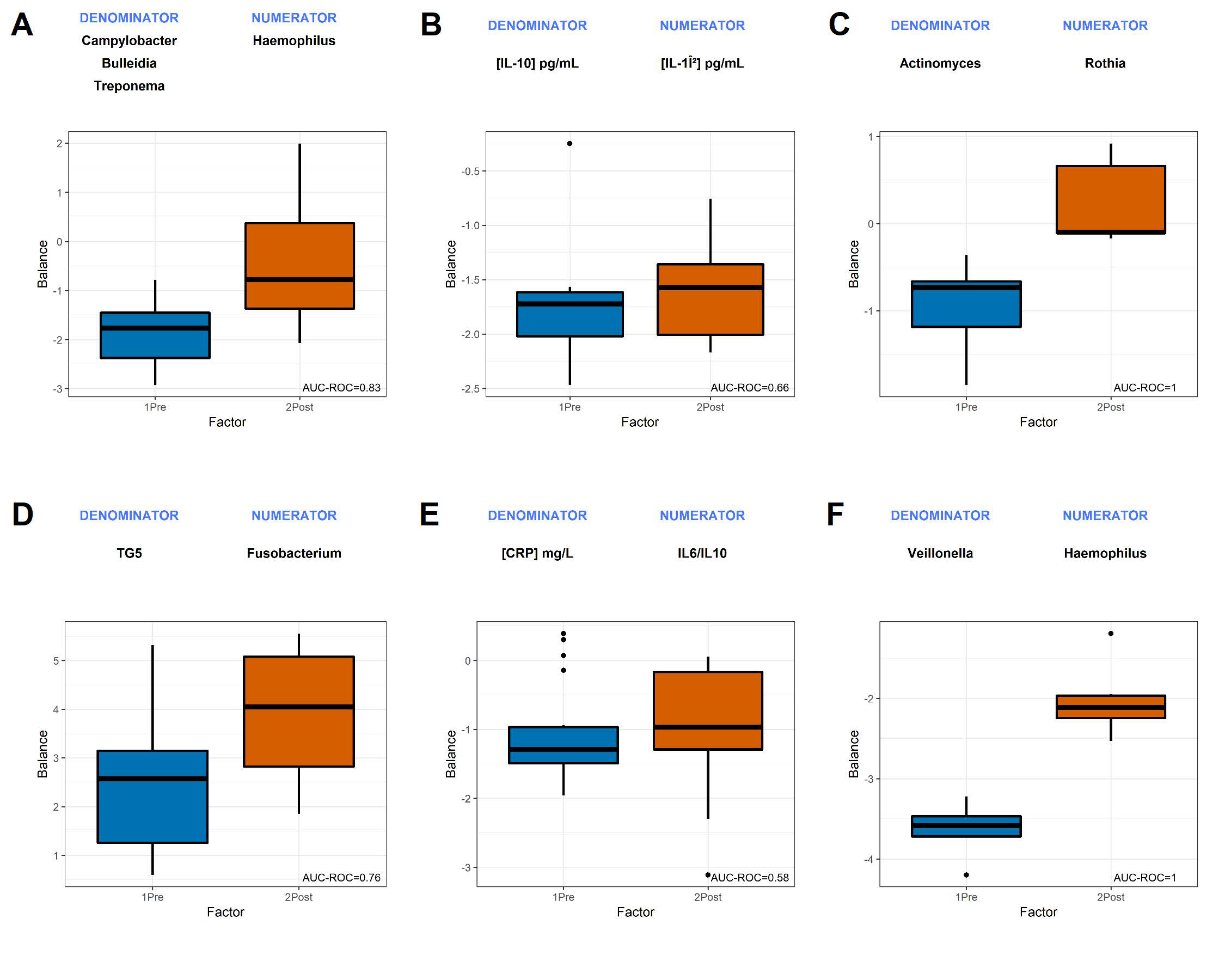

Supplement: Supplementary Figure 1 — NMDS ordination plots showing clustering of PT samples by pocket depth. Columns correspond to dataset type; 16S, Cytokine, and Metagenomic datasets are columns one, two, and three, respectively (n = 60, 104, and 22). [file Data_Sheet_2.ZIP › Supplemental Figures and Tables/Supplemental_Figure_7.tif]

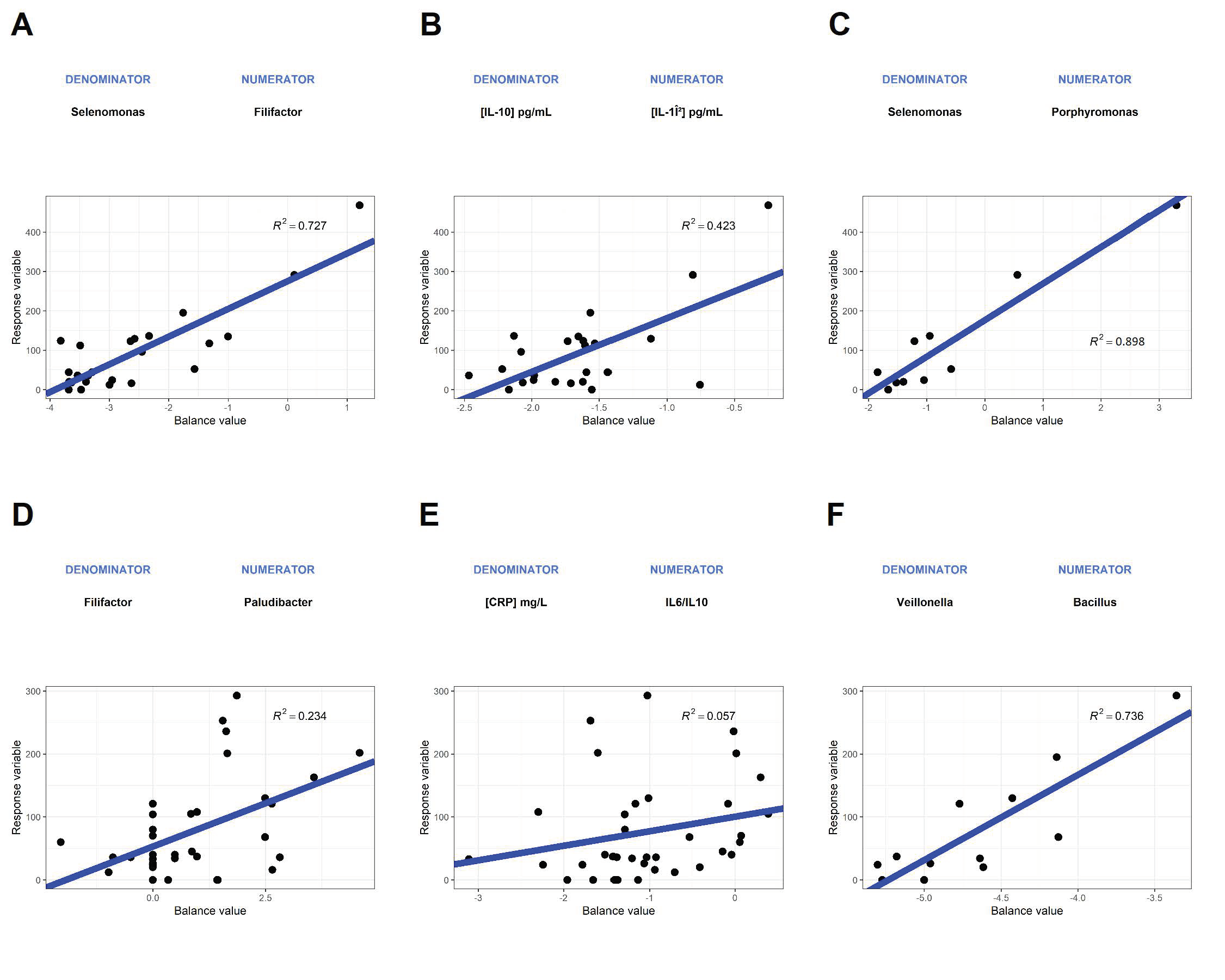

Supplement: Supplementary Figure 1 — NMDS ordination plots showing clustering of PT samples by pocket depth. Columns correspond to dataset type; 16S, Cytokine, and Metagenomic datasets are columns one, two, and three, respectively (n = 60, 104, and 22). [file Data_Sheet_2.ZIP › Supplemental Figures and Tables/Supplemental_Figure_8.tif]

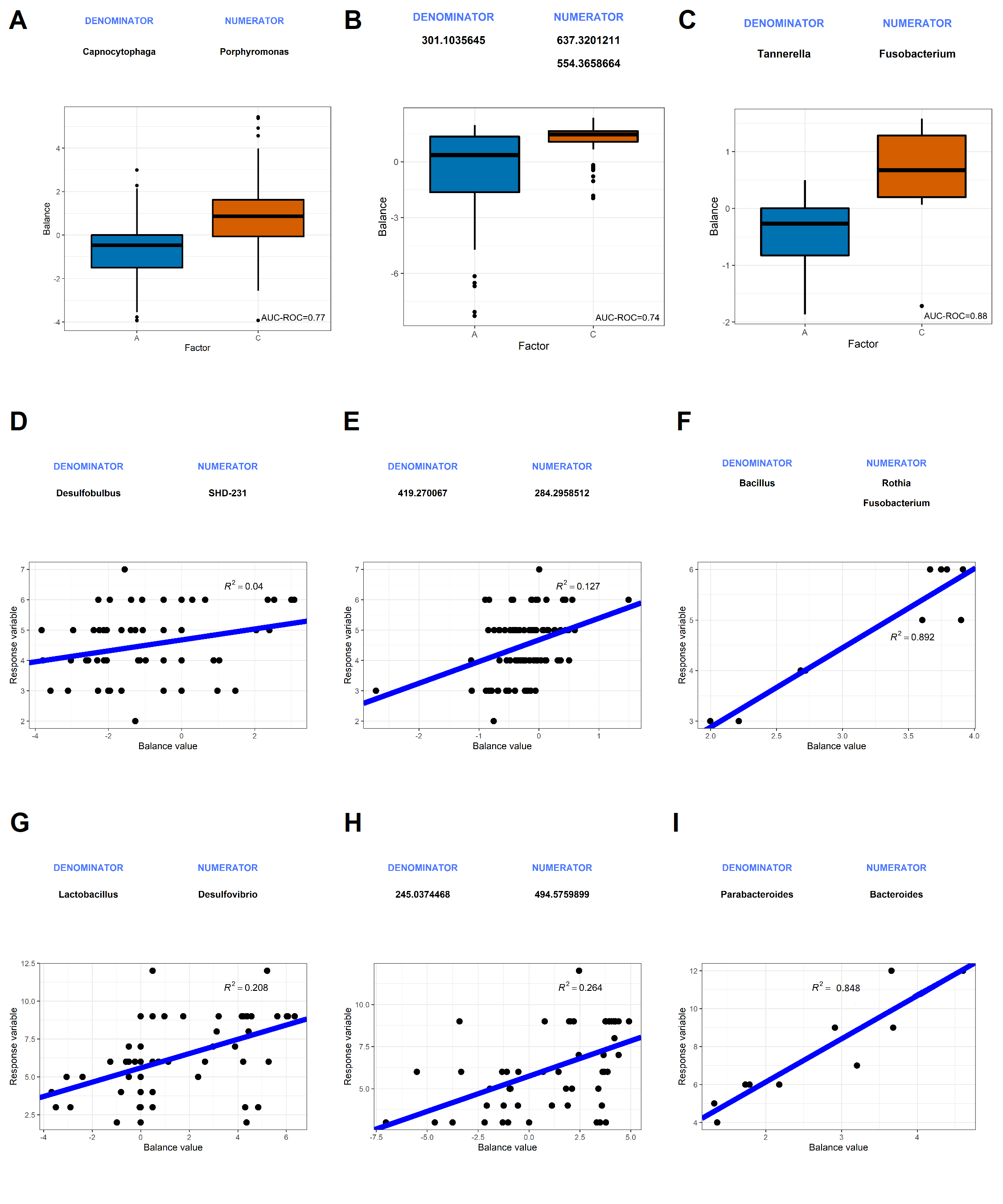

Supplement: Supplementary Figure 1 — NMDS ordination plots showing clustering of PT samples by pocket depth. Columns correspond to dataset type; 16S, Cytokine, and Metagenomic datasets are columns one, two, and three, respectively (n = 60, 104, and 22). [file Data_Sheet_2.ZIP › Supplemental Figures and Tables/Supplemental_Figure_9.tif]
