# Supplementary material for: Compositional Data Analysis of Periodontal Disease Microbial Communities
Source: Front Microbiol. 2021 May 17;12:617949. doi: 10.3389/fmicb.2021.617949 (PMC8165185; doi:10.3389/fmicb.2021.617949)
Supplement: Supplementary file 3 [file Data_Sheet_3.ZIP › jupyternotebook_and_files/PT_SHT_selbal_Rmarkdown.nb.html]

R selbal Notebook


Code 

- Show All Code
- Hide All Code
- Download Rmd

# R selbal Notebook


```
#import packages
library(propr)
library(magrittr)
library(zCompositions)
```


```
Loading required package: MASS
Loading required package: NADA
Loading required package: survival

Attaching package: 㤼㸱NADA㤼㸲

The following object is masked from 㤼㸱package:stats㤼㸲:

    cor

Loading required package: truncnorm
```


```
library(magrittr)
library(dplyr)
```


```
Attaching package: 㤼㸱dplyr㤼㸲

The following object is masked from 㤼㸱package:MASS㤼㸲:

    select

The following objects are masked from 㤼㸱package:stats㤼㸲:

    filter, lag

The following objects are masked from 㤼㸱package:base㤼㸲:

    intersect, setdiff, setequal, union
```


```
library(ggplot2)
```


```
package 㤼㸱ggplot2㤼㸲 was built under R version 4.0.2
```


```
library(tidyverse)
```


```
Registered S3 methods overwritten by 'dbplyr':
  method         from
  print.tbl_lazy     
  print.tbl_sql      
-- Attaching packages --------------------------------------- tidyverse 1.3.0 --
v tibble  3.0.1     v purrr   0.3.4
v tidyr   1.1.0     v stringr 1.4.0
v readr   1.3.1     v forcats 0.5.0
-- Conflicts ------------------------------------------ tidyverse_conflicts() --
x tidyr::extract()   masks magrittr::extract()
x dplyr::filter()    masks stats::filter()
x dplyr::lag()       masks stats::lag()
x dplyr::select()    masks MASS::select()
x purrr::set_names() masks magrittr::set_names()
x purrr::simplify()  masks propr::simplify()
```


```
library(readr)
library(mixOmics)
```


```
Loading required package: lattice

Attaching package: 㤼㸱lattice㤼㸲

The following object is masked from 㤼㸱package:propr㤼㸲:

    parallel


Loaded mixOmics 6.12.1
Thank you for using mixOmics!
Tutorials: http://mixomics.org
Bookdown vignette: https://mixomicsteam.github.io/Bookdown
Questions, issues: Follow the prompts at http://mixomics.org/contact-us
Cite us:  citation('mixOmics')


Attaching package: 㤼㸱mixOmics㤼㸲

The following object is masked from 㤼㸱package:purrr㤼㸲:

    map

The following object is masked from 㤼㸱package:propr㤼㸲:

    pca
```


```
library(selbal)
library(vegan)
```


```
Loading required package: permute
This is vegan 2.5-6
```


```
library(scales)
```


```
Attaching package: 㤼㸱scales㤼㸲

The following object is masked from 㤼㸱package:purrr㤼㸲:

    discard

The following object is masked from 㤼㸱package:readr㤼㸲:

    col_factor
```


```
library(ggthemes)
```


```
package 㤼㸱ggthemes㤼㸲 was built under R version 4.0.2
```


```
require(gtools)
```


```
Loading required package: gtools

Attaching package: 㤼㸱gtools㤼㸲

The following object is masked from 㤼㸱package:permute㤼㸲:

    permute
```


```
library(viridis)
```


```
package 㤼㸱viridis㤼㸲 was built under R version 4.0.2Loading required package: viridisLite

Attaching package: 㤼㸱viridis㤼㸲

The following object is masked from 㤼㸱package:scales㤼㸲:

    viridis_pal
```


```
library(ggpubr)
```


```
package 㤼㸱ggpubr㤼㸲 was built under R version 4.0.2
```


```
library(corrplot)
```


```
package 㤼㸱corrplot㤼㸲 was built under R version 4.0.2corrplot 0.84 loaded
```


```
library(psych)
```


```
package 㤼㸱psych㤼㸲 was built under R version 4.0.2
Attaching package: 㤼㸱psych㤼㸲

The following object is masked from 㤼㸱package:gtools㤼㸲:

    logit

The following objects are masked from 㤼㸱package:scales㤼㸲:

    alpha, rescale

The following object is masked from 㤼㸱package:mixOmics㤼㸲:

    pca

The following objects are masked from 㤼㸱package:ggplot2㤼㸲:

    %+%, alpha

The following object is masked from 㤼㸱package:propr㤼㸲:

    pca
```


```
library(magick)
```


```
package 㤼㸱magick㤼㸲 was built under R version 4.0.2Linking to ImageMagick 6.9.9.14
Enabled features: cairo, freetype, fftw, ghostscript, lcms, pango, rsvg, webp
Disabled features: fontconfig, x11
```


```
library(pdftools)
```


```
package 㤼㸱pdftools㤼㸲 was built under R version 4.0.2Using poppler version 0.73.0
```


```
library(png)
library(grid)
library(gridExtra)
```


```
Attaching package: 㤼㸱gridExtra㤼㸲

The following object is masked from 㤼㸱package:dplyr㤼㸲:

    combine
```


This is an R Markdown Notebook. When you execute code within the notebook, the results appear beneath the code.

Try executing this chunk by clicking the *Run* button within the chunk or by placing your cursor inside it and pressing *Ctrl+Shift+Enter*.


```
#PT data read in
#tables
PT_bact_genus_sum_untx_selbal <- read.table('./files/2020-09-18_PT_16S_OTU_genus_Tabl.csv',sep=',',row.names = 1,header = 1,check.names=FALSE)
PT_cyto_untx_t <- read.table('./files/2020-09-18_PT_cyto_table.csv',sep=',',header =1,row.names = 1,check.names = F,na.strings = 'ND')
PT_cyto_untx <- t(PT_cyto_untx_t)
PT_metagen_genus_10_reduce_slim <- read.table('./files/2020-09-18_PT_metagen_genus_table.csv',sep=',',row.names = 1,header = 1,check.names=FALSE)
```


```
#PT read in and filter maps
#import sample maps
PT_bact_map <- read.table('./files/Schwarzberg_et_al_mappingfile.txt',sep='\t',header =1,row.names = 1)
PT_bact_map_ordered <- PT_bact_map[order(PT_bact_map$Sample),]
PT_bact_map_large <- read.csv('./files/all76pt_map.txt.csv', row.names=1,header=1)
PT_cyto_map <- as.data.frame(read.csv('./files/PDValues_LDF_CytokineResults.csv'))
#dim(cyto_df)
#duplicate map to relect cyto kine samples in table
PT_cyto_map_dupl <- PT_cyto_map[rep(row.names(PT_cyto_map), PT_cyto_map$Case_status+1),] %>%
  arrange(PID) 
PT_cyto_map_dupl$Case_status[duplicated(PT_cyto_map_dupl[1:2])] <- PT_cyto_map_dupl$Case_status[duplicated(PT_cyto_map_dupl[1:2])] +1
PT_metagen_map<- read.table('./files/mapping_file_PT.txt',sep='\t',header =1,row.names = 1)
PT_metagen_map_ordered <- PT_metagen_map[order(row.names(PT_metagen_map)),order(colnames(PT_metagen_map))]
PT_metagen_map_ordered$PerioTreatment <- gsub('1|2','',PT_metagen_map_ordered$PerioTreatment)

#filter metagen
PT_metagen_df_filtered <- PT_metagen_genus_10_reduce_slim[,colnames(PT_metagen_genus_10_reduce_slim) %in%row.names(PT_metagen_map_ordered)] 

#Get interesting PD from cytokine map
PT_bact_map_PD <- PT_cyto_map_dupl[1:94,] 

###which samples to keep; only analyzing improved and worsened; 
PT_cyto_map_improved_worsened <- PT_cyto_map_dupl[grep('[1-9]',PT_cyto_map_dupl$PDSumdiff),]
PT_cyto_map_improved_worsened$PocketResponse <- 'Improved'
PT_cyto_map_improved_worsened$PocketResponse[PT_cyto_map_improved_worsened$PDSumdiff < 0] <- 'Worsened'
PT_cyto_map_improved_worsened$PDSum <- as.vector(rbind(PT_cyto_map_improved_worsened$PrePDsum[seq(1,nrow(PT_cyto_map_improved_worsened),2)],PT_cyto_map_improved_worsened$PostPDsum[seq(1,nrow(PT_cyto_map_improved_worsened),2)]))


PT_bact_map_improved_worsened <- PT_bact_map_large[grep( 'Improved|Worsened',PT_bact_map_large$OverallResponse),]
PT_bact_map_improved_worsened$metagenDescrip <- paste(rownames(PT_bact_map_improved_worsened),'.kraken',sep = '')
#bact_map_PD$PID %in% as.numeric(grep( 'Improved|Worsened',bact_map_large$OverallResponse))

PT_bact_map_PD_there <- PT_bact_map_PD[bact_map_PD$PID%in%as.numeric(gsub( 'A|B|"0','',row.names(PT_bact_map_improved_worsened))),]
```


```
###Selbal
#read in data
#import all clr OTU tables and metabolite table
bact_df <- PT_bact_genus_sum_untx_selbal 
bact_df_ordered <- bact_df[order(row.names(bact_df)),order(colnames(bact_df))]
cyto_df <- t(PT_cyto_untx_t)
metagen_df <- PT_metagen_genus_10_reduce_slim 
metagen_df_ordered <- metagen_df[order(row.names(metagen_df)), order(colnames(metagen_df))]

#filter metagen
metagen_df_filtered <- metagen_df_ordered[,colnames(metagen_df_ordered)%in%row.names(metagen_map_ordered)] 

bact_genus_sum <- bact_df_ordered[,order(colnames(bact_df_ordered))]###add healthy or not to colnames
```


```
#make Pre Post dicotomous variable 
bact_map_ordered <- PT_bact_map_improved_worsened#[order(bact_map$Sample),] 
cyto_map_to_remove <- PT_cyto_map[(PT_cyto_map$Case_status ==0),]
bact_map_ordered_PrePost <- PT_bact_map_ordered[!(bact_map_ordered$PerioTreatment=="Healthy"),]
bact_map_ordered_PrePost$Treatment <- ifelse(bact_map_ordered_PrePost$PerioTreatment=='Pre','Pre','Post') 
cyto_map_PrePost <- PT_cyto_map_dupl[!cyto_map_dupl$Case_status==0,]
cyto_map_Pre <- PT_cyto_map[cyto_map$Case_status==1,]
cyto_map_Post <- PT_cyto_map[cyto_map$Case_status==1,]
bact_map_Pre <- bact_map_ordered_PrePost[bact_map_ordered_PrePost$PerioTreatment=='Pre',]
bact_map_Post <-bact_map_ordered_PrePost[bact_map_ordered_PrePost$PerioTreatment=='Post',]
```


```
#get only Pre Post 
bact_genus_sum_PrePost <- bact_genus_sum[ , which(colnames(bact_genus_sum) %in% row.names(bact_map_improved_worsened))]
#bact_genus_sum_PrePost
#bact_map_ordered_PrePost
bact_genus_sum_Pre <- bact_genus_sum[, which(colnames(bact_genus_sum) %in% row.names(bact_map_Pre))]
bact_genus_sum_Pre<- bact_genus_sum_Pre[, order(colnames(bact_genus_sum_Pre))]

bact_genus_sum_Post <- bact_genus_sum[, which(colnames(bact_genus_sum) %in% row.names(bact_map_Post))]
bact_genus_sum_Post <- bact_genus_sum_Post[, order(colnames(bact_genus_sum_Post))]
```


```
bact_genus_t <- t(bact_genus_sum_PrePost)
cyto_t <- t(cyto_untx)
metagen_genus_t <- t(metagen_df_filtered)

bact_pre <- bact_genus_t[grep('A', row.names(bact_genus_t)),]
cyto_pre <- cyto_t[grep('A', row.names(cyto_t)),]
metagen_pre <- metagen_genus_t[grep('A', row.names(metagen_genus_t)),]
# bact_cyto_pre <- rmrn_bact_cyto[grep('A', row.names(rmrn_bact_cyto)),]
# bact_metagen_pre <- rmrn_bact_metagen[grep('A', row.names(rmrn_bact_cyto)),]
bact_post <- bact_genus_t[grep('B', row.names(bact_genus_t)),]
cyto_post <- cyto_t[grep('B', row.names(cyto_t)),]
metagen_post <- metagen_genus_t[grep('B', row.names(metagen_genus_t)),]
# bact_cyto_post <- rmrn_bact_cyto[grep('B', row.names(rmrn_bact_cyto)),]
# bact_metagen_post <- rmrn_bact_metagen[grep('B', row.names(rmrn_bact_cyto)),]
```


```
bact_improved <- bact_genus_t[rownames(bact_genus_t)%in%rownames(bact_map_improved_worsened[bact_map_improved_worsened$OverallResponse =='Improved',]),]
bact_worsened <- bact_genus_t[rownames(bact_genus_t)%in%rownames(bact_map_improved_worsened[bact_map_improved_worsened$OverallResponse =='Worsened',]),]
bact_pre_improved <- bact_pre[rownames(bact_map_improved_worsened[bact_map_improved_worsened$OverallResponse =='Improved' &bact_map_improved_worsened$PerioTreatment=='Pre',]),]
# [rownames(bact_map_improved_worsened)[grep('A',rownames(bact_map_improved_worsened[grep('Improved',bact_map_improved_worsened$OverallResponse),]))],]
bact_pre_worsened <- bact_pre[rownames(bact_map_improved_worsened[bact_map_improved_worsened$OverallResponse =='Worsened' &bact_map_improved_worsened$PerioTreatment=='Pre',]),]
cyto_improved <- cyto_t[rownames(bact_map_improved_worsened[bact_map_improved_worsened$OverallResponse =='Improved',]),]
cyto_worsened <- cyto_t[rownames(bact_map_improved_worsened[bact_map_improved_worsened$OverallResponse =='Worsened',]),]
cyto_pre_improved <- cyto_t[rownames(bact_map_improved_worsened[bact_map_improved_worsened$OverallResponse =='Improved'&bact_map_improved_worsened$PerioTreatment=='Pre',]),]
cyto_pre_worsened <- cyto_t[rownames(bact_map_improved_worsened[bact_map_improved_worsened$OverallResponse =='Worsened' &bact_map_improved_worsened$PerioTreatment=='Pre',]),]
metagen_improved <-metagen_genus_t[rownames(metagen_genus_t) %in% rownames(metagen_map_ordered[metagen_map_ordered$OverallResponse =='Improved' ,]),]
metagen_worsened <- metagen_genus_t[rownames(metagen_genus_t) %in% rownames(metagen_map_ordered[metagen_map_ordered$OverallResponse =='Worsened' ,]),]
metagen_pre_improved <-metagen_genus_t[rownames(metagen_genus_t) %in% rownames(metagen_map_ordered[metagen_map_ordered$OverallResponse =='Improved' & metagen_map_ordered$PerioTreatment=='Pre',]),]
metagen_pre_worsened <- metagen_genus_t[rownames(metagen_genus_t) %in% rownames(metagen_map_ordered[metagen_map_ordered$OverallResponse =='Worsened' & metagen_map_ordered$PerioTreatment=='Pre',]),]
bact_post_improved <- bact_post[rownames(bact_map_improved_worsened[bact_map_improved_worsened$OverallResponse =='Improved' &bact_map_improved_worsened$PerioTreatment=='Post',]),]
bact_post_worsened <- bact_post[rownames(bact_map_improved_worsened[bact_map_improved_worsened$OverallResponse =='Worsened' &bact_map_improved_worsened$PerioTreatment=='Post',]),]
cyto_post_improved <- cyto_t[rownames(bact_map_improved_worsened[bact_map_improved_worsened$OverallResponse =='Improved' &bact_map_improved_worsened$PerioTreatment=='Post',]),]
cyto_post_worsened <- cyto_t[rownames(bact_map_improved_worsened[bact_map_improved_worsened$OverallResponse =='Worsened' &bact_map_improved_worsened$PerioTreatment=='Post',]),]
metagen_post_improved <- metagen_genus_t[rownames(metagen_genus_t) %in% rownames(metagen_map_ordered[metagen_map_ordered$OverallResponse =='Improved' &metagen_map_ordered$PerioTreatment=='Post',]),]
metagen_post_worsened <- metagen_genus_t[rownames(metagen_genus_t) %in% rownames(metagen_map_ordered[metagen_map_ordered$OverallResponse =='Worsened' &metagen_map_ordered$PerioTreatment=='Post',]),]
```


```
#######Pre vs Post
# #selbal needs vector with response variable. Make vectors: (as factor)
# #can only do dichotomous variables- Pre-Post and continuous- pocketdepth
# 
# #selbal needs two inputs- matrix with samples as rows and taxa as column
#setup 
bact_genus_sum <- bact_genus_sum_PrePost 
bact_genus_sum_t <- t(bact_genus_sum[,order(colnames(bact_genus_sum))])###add healthy or not to colnames
bact_genus_sum_t <- as.data.frame(bact_genus_sum_t)#[ -grep("TP0", row.names(bact_genus_sum_t)),])
bact_map_improved_worsened
```


```
bact_map <- bact_map_improved_worsened#[ -grep("TP0", row.names(bact_map)),]
bact_treatment_var <- as.factor(bact_map$PerioTreatment)
bact_treatment_var <- as.factor(ifelse(bact_treatment_var == 'Post','2Post','1Pre'))
require(gtools)
#bact_genus_sum_no0<-bact_genus_sum_no0[mixedsort( rownames( bact_map_no0 ) ),]
nrow(bact_genus_sum)
```


```
[1] 43
```


```
length(bact_treatment_var)
```


```
[1] 60
```


```
bact_cv_treatment <- selbal.cv(x=bact_genus_sum_t, y=bact_treatment_var,n.fold=3, zero.rep="bayes",seed = 1,col = c("#0072B2","#D55E00"))
```


```
############################################################### 
 STARTING selbal.cv FUNCTION 
###############################################################

#-------------------------------------------------------------# 
# ZERO REPLACEMENT . . .


, . . . FINISHED. 
#-------------------------------------------------------------#

#-------------------------------------------------------------# 
# Starting the cross - validation procedure . . .
```


```
already exporting variable(s): logit.acc
```


```
 . . . finished. 
#-------------------------------------------------------------# 
###############################################################

 The optimal number of variables is: 5
```


```
Setting levels: control = 1Pre, case = 2Post
Setting direction: controls < cases
Setting levels: control = 0, case = 1
Setting direction: controls < cases
```


```
############################################################### 
 . . . FINISHED. 
###############################################################
```


```
bact_cv_treatment$accuracy.nvar
```


```
#barplot representing the frequency of the variables selected 
#in some step of the CV process 
bact_cv_treatment$var.barplot
grid.draw(bact_cv_treatment$global.plot)
```


```
plot.tab(bact_cv_treatment$cv.tab)
bact_cv_treatment$glm
```


```
Call:  glm(formula = numy ~ ., family = f.class, data = U)

Coefficients:
(Intercept)           V1  
     0.9095       0.7427  

Degrees of Freedom: 59 Total (i.e. Null);  58 Residual
Null Deviance:      83.18 
Residual Deviance: 65.04    AIC: 69.04
```


```
bact_cv_treatment$global.balance
```


```
#selbal make pretty
png('./files/PT_selbal/2020-09-21_cb_selbal_bact_treatment.png', width=4800, height=2900, res=315)
grid.draw(bact_cv_treatment$global.plot)
```


```
dev.off()
```


```
png 
  2
```


```
png('./files/PT_selbal/2020-09-21_b_cb_selbal_bact_treatment.png', width=2500, height=2500, res=315)
```


```
grid.draw(bact_cv_treatment$global.plot)
dev.off()
```


```
png 
  2
```


```
# #selbal needs vector with response variable. Make vectors: (as factor)
# #can only do dichotomous variables- wet-dry and continuous- time
# depth_var_bact <- as.numeric(((bact_map_improved_worsened$AvgPocketDepth)))#*.1)
# 
# #selbal needs two inputs- matrix with samples as rows and taxa as column

########now do PD/PD sum
bact_map_improved_worsened$PDsum
```


```
 [1]  16  12 124  20 136  18   0  20  45  60  20   0 112  96  36   0  52  24 195 129  24
[22]  40   0  12   0 130 123  44  37 121 202 253 135 117 468 291  16  36   0  24 105 201
[43] 163 236  34 293   0  80  36 121  36  44  40  33  70 104  36 108  26  68
```


```
pdsum_var_bact <- as.numeric(((bact_map_improved_worsened$PDsum)))#*.1)
ncol(bact_genus_sum_t)
```


```
[1] 48
```


```
nrow(bact_genus_sum_t)
```


```
[1] 60
```


```
length(pdsum_var_bact)
```


```
[1] 60
```


```
cv_bact_pdsum <- selbal.cv(x=bact_genus_sum_t, y=pdsum_var_bact, zero.rep = 'bayes',seed = 1,col = c("#0072B2","#D55E00"))
```


```
############################################################### 
 STARTING selbal.cv FUNCTION 
###############################################################

#-------------------------------------------------------------# 
# ZERO REPLACEMENT . . .


, . . . FINISHED. 
#-------------------------------------------------------------#

#-------------------------------------------------------------# 
# Starting the cross - validation procedure . . .
```


```
already exporting variable(s): logit.acc
```


```
 . . . finished. 
#-------------------------------------------------------------# 
###############################################################

 The optimal number of variables is: 2 


############################################################### 
 . . . FINISHED. 
###############################################################
```


```
cv_bact_pdsum$accuracy.nvar
```


```
cv_bact_pdsum$var.barplot
cv_bact_pdsum$glm
```


```
Call:  glm(formula = numy ~ ., family = f.class, data = U)

Coefficients:
(Intercept)           V1  
     152.24        27.58  

Degrees of Freedom: 59 Total (i.e. Null);  58 Residual
Null Deviance:      479900 
Residual Deviance: 397300   AIC: 704.2
```


```
grid.draw(cv_bact_pdsum$global.plot)
```


```
plot.tab(cv_bact_pdsum$cv.tab)

#make pretty selbal  cv_bact_pdsum
png('./files/PT_selbal/2020-09-20_cb_selbal_cv_bact_pdsum.png', width=2500, height=2500, res=400)
```


```
grid.draw(cv_bact_pdsum$global.plot)
dev.off()
```


```
png 
  2
```


```
#selbal posket improv worsened
bact_improv_wor_var <- as.factor(bact_map_improved_worsened$OverallResponse)
cv_bact_improv_wor <- selbal.cv(x=bact_genus_sum_t,y=bact_improv_wor_var,zero.rep = "bayes",seed = 1,col = c("#0072B2","#D55E00"))
```


```
############################################################### 
 STARTING selbal.cv FUNCTION 
###############################################################

#-------------------------------------------------------------# 
# ZERO REPLACEMENT . . .


, . . . FINISHED. 
#-------------------------------------------------------------#

#-------------------------------------------------------------# 
# Starting the cross - validation procedure . . .
```


```
already exporting variable(s): logit.acc
```


```
 . . . finished. 
#-------------------------------------------------------------# 
###############################################################

 The optimal number of variables is: 4
```


```
Setting levels: control = Improved, case = Worsened
Setting direction: controls < cases
Setting levels: control = 0, case = 1
Setting direction: controls < cases
```


```
############################################################### 
 . . . FINISHED. 
###############################################################
```


```
cv_bact_improv_wor$accuracy.nvar
```


```
cv_bact_improv_wor$var.barplot
cv_bact_improv_wor$glm
```


```
Call:  glm(formula = numy ~ ., family = f.class, data = U)

Coefficients:
(Intercept)           V1  
     0.4475       0.9661  

Degrees of Freedom: 59 Total (i.e. Null);  58 Residual
Null Deviance:      80.76 
Residual Deviance: 67.93    AIC: 71.93
```


```
grid.draw(cv_bact_improv_wor$global.plot)
```


```
plot.tab(cv_bact_improv_wor$cv.tab)

#make pretty selbal  cv_bact_pdsum
png('./files/PT_selbal/2020-09-20_cb_selbal_cv_bact_improv_wor.png', width=5000, height=2900, res=400)
```


```
grid.draw(cv_bact_improv_wor$global.plot)
dev.off()
```


```
png 
  2
```


```
png('./files/PT_selbal/2020-09-20_cb_selbal_cv_bact_improv_wor.png', width=2500, height=2500, res=400)
grid.draw(cv_bact_improv_wor$global.plot)
```


```
dev.off()
```


```
png 
  2
```


```
## Improved prevspost
var <- as.factor(bact_map_improved_worsened['Improved'==bact_map_improved_worsened$OverallResponse,]$PerioTreatment)
var <- as.factor(ifelse(var == 'Post','2Post','1Pre'))
sebal_t <- (bact_improved)

#selbal 
cv_bact_improved <- selbal.cv(x=sebal_t, y=var, zero.rep="one",seed = 1,col = c("#0072B2","#D55E00"))
```


```
############################################################### 
 STARTING selbal.cv FUNCTION 
###############################################################

#-------------------------------------------------------------# 
# ZERO REPLACEMENT . . .


, . . . FINISHED. 
#-------------------------------------------------------------#

#-------------------------------------------------------------# 
# Starting the cross - validation procedure . . .
```


```
already exporting variable(s): logit.acc
```


```
 . . . finished. 
#-------------------------------------------------------------# 
###############################################################

 The optimal number of variables is: 4
```


```
glm.fit: fitted probabilities numerically 0 or 1 occurredSetting levels: control = 1Pre, case = 2Post
Setting direction: controls < cases
Setting levels: control = 0, case = 1
Setting direction: controls < cases
```


```
############################################################### 
 . . . FINISHED. 
###############################################################
```


```
cv_bact_improved$accuracy.nvar
```


```
cv_bact_improved$var.barplot
grid.draw(cv_bact_improved$global.plot)
```


```
plot.tab(cv_bact_improved$cv.tab)
cv_bact_improved$glm
```


```
Call:  glm(formula = numy ~ ., family = f.class, data = U)

Coefficients:
(Intercept)           V1  
      2.494        1.913  

Degrees of Freedom: 23 Total (i.e. Null);  22 Residual
Null Deviance:      33.27 
Residual Deviance: 22.18    AIC: 26.18
```


```
#make pretty selbal  cv_bact
png('./files/PT_selbal/2020-09-20_cb_selbal_cv_bact_improved_prepost.png', width=5000, height=2900, res=400)
```


```
grid.draw(cv_bact_improved$global.plot)
dev.off()
```


```
png 
  2
```


```
png('./files/PT_selbal/2020-09-20_cb_selbal_cv_bact_improved_prepost.png', width=2500, height=2500, res=400)
grid.draw(cv_bact_improved$global.plot)
```


```
dev.off()
```


```
png 
  2
```


```
## Worsened prevspost
var <- as.factor(bact_map_improved_worsened['Worsened'==bact_map_improved_worsened$OverallResponse,]$PerioTreatment)
var <- as.factor(ifelse(var == 'Post','2Post','1Pre'))
sebal_t <- (bact_worsened)

#selbal time wet
cv_bact_worsened <- selbal.cv(x=sebal_t, y=var, zero.rep="bayes",seed = 1,col = c("#0072B2","#D55E00"))
```


```
############################################################### 
 STARTING selbal.cv FUNCTION 
###############################################################

#-------------------------------------------------------------# 
# ZERO REPLACEMENT . . .


, . . . FINISHED. 
#-------------------------------------------------------------#

#-------------------------------------------------------------# 
# Starting the cross - validation procedure . . .
```


```
already exporting variable(s): logit.acc
```


```
 . . . finished. 
#-------------------------------------------------------------# 
###############################################################

 The optimal number of variables is: 2
```


```
Setting levels: control = 1Pre, case = 2Post
Setting direction: controls < cases
Setting levels: control = 0, case = 1
Setting direction: controls < cases
```


```
############################################################### 
 . . . FINISHED. 
###############################################################
```


```
cv_bact_worsened$accuracy.nvar
```


```
cv_bact_worsened$var.barplot
grid.draw(cv_bact_worsened$global.plot)
```


```
plot.tab(cv_bact_worsened$cv.tab)
cv_bact_worsened$glm
```


```
Call:  glm(formula = numy ~ ., family = f.class, data = U)

Coefficients:
(Intercept)           V1  
    -2.1902       0.6892  

Degrees of Freedom: 35 Total (i.e. Null);  34 Residual
Null Deviance:      49.91 
Residual Deviance: 42.29    AIC: 46.29
```


```
#make pretty selbal  cv_bact_depth
png('./files/PT_selbal/2020-09-20_cb_selbal_cv_bact_worsened_prepost.png', width=5000, height=2900, res=400)
```


```
grid.draw(cv_bact_worsened$global.plot)
dev.off()
```


```
png 
  2
```


```
png('./files/PT_selbal/2020-09-20_b_cb_selbal_cv_bact_worsened_prepost.png', width=2500, height=2500, res=400)
grid.draw(cv_bact_worsened$global.plot)
```


```
dev.off()
```


```
png 
  2
```


```
## Improved sum diff or sum pd ?
bact_map_improved_worsened['Improved'==bact_map_improved_worsened$OverallResponse,]$PDsum
```


```
 [1]  16  12 124  20 136  18  20   0 112  96  36   0  52  24 195 129 123  44 135 117 468
[22] 291  36  44
```


```
samples <- row.names(bact_map_improved_worsened['Improved'==bact_map$OverallResponse,])
var <- as.numeric((bact_map_improved_worsened[samples,]$PDsum))
sebal_t <- (bact_improved)
# sebal__t <- sebal_t[,colSums(sebal_t == 0) <= 0.9 *nrow(sebal_t)]

#selbal time wet
cv_bact_improved_pdsum <- selbal.cv(x=sebal_t, y=var, zero.rep="one",seed = 1,col = c("#0072B2","#D55E00"))
```


```
############################################################### 
 STARTING selbal.cv FUNCTION 
###############################################################

#-------------------------------------------------------------# 
# ZERO REPLACEMENT . . .


, . . . FINISHED. 
#-------------------------------------------------------------#

#-------------------------------------------------------------# 
# Starting the cross - validation procedure . . .
```


```
already exporting variable(s): logit.acc
```


```
 . . . finished. 
#-------------------------------------------------------------# 
###############################################################

 The optimal number of variables is: 2 


############################################################### 
 . . . FINISHED. 
###############################################################
```


```
cv_bact_improved_pdsum$accuracy.nvar
```


```
cv_bact_improved_pdsum$var.barplot
grid.draw(cv_bact_improved_pdsum$global.plot)
```


```
plot.tab(cv_bact_improved_pdsum$cv.tab)
cv_bact_improved_pdsum$glm
```


```
Call:  glm(formula = numy ~ ., family = f.class, data = U)

Coefficients:
(Intercept)           V1  
      275.3         70.4  

Degrees of Freedom: 23 Total (i.e. Null);  22 Residual
Null Deviance:      261800 
Residual Deviance: 71570    AIC: 266.1
```


```
#make pretty selbal  cv_bact_depth
png('./files/PT_selbal/2020-09-20_cb_selbal_cv_bact_improved_PDsum.png', width=2500, height=2500, res=400)
```


```
grid.draw(cv_bact_improved_pdsum$global.plot)
dev.off()
```


```
png 
  2
```


```
## worsened sum diff or sum pd ?
bact_map_improved_worsened['Worsened'==bact_map_improved_worsened$OverallResponse,]$PDsum
```


```
 [1]   0  20  45  60  24  40   0  12   0 130  37 121 202 253  16  36   0  24 105 201 163
[22] 236  34 293   0  80  36 121  40  33  70 104  36 108  26  68
```


```
samples <- row.names(bact_map_improved_worsened['Worsened'==bact_map$OverallResponse,])
var <- as.numeric((bact_map_improved_worsened[samples,]$PDsum))
sebal_t <- (bact_worsened)

#selbal time wet
cv_bact_worsened_pdsum <- selbal.cv(x=sebal_t, y=var, zero.rep="one",seed = 1,col = c("#0072B2","#D55E00"))
```


```
############################################################### 
 STARTING selbal.cv FUNCTION 
###############################################################

#-------------------------------------------------------------# 
# ZERO REPLACEMENT . . .


, . . . FINISHED. 
#-------------------------------------------------------------#

#-------------------------------------------------------------# 
# Starting the cross - validation procedure . . .
```


```
already exporting variable(s): logit.acc
```


```
 . . . finished. 
#-------------------------------------------------------------# 
###############################################################

 The optimal number of variables is: 2 


############################################################### 
 . . . FINISHED. 
###############################################################
```


```
cv_bact_worsened_pdsum$accuracy.nvar
```


```
cv_bact_worsened_pdsum$var.barplot
grid.draw(cv_bact_worsened_pdsum$global.plot)
```


```
plot.tab(cv_bact_worsened_pdsum$cv.tab)
cv_bact_worsened_pdsum$glm
```


```
Call:  glm(formula = numy ~ ., family = f.class, data = U)

Coefficients:
(Intercept)           V1  
      53.05        27.48  

Degrees of Freedom: 35 Total (i.e. Null);  34 Residual
Null Deviance:      214100 
Residual Deviance: 164000   AIC: 411.4
```


```
#make pretty selbal  cv_bact_depth
png('./files/PT_selbal/2020-09-20_cb_selbal_cv_bact_worsened_PDsum.png', width=2500, height=2500, res=400)
```


```
grid.draw(cv_bact_worsened_pdsum$global.plot)
dev.off()
```


```
png 
  2
```


```
#make Pre Post dicotomous variable 
cyto_untx_t_prepost <- cyto_t[as.numeric(gsub('A|B','',rownames(cyto_t)))%in%cyto_map_improved_worsened$PID,]

##regular Prepost 
prepost_var <- as.factor(cyto_map_improved_worsened$Case_status)
prepost_var <- as.factor(ifelse(prepost_var == 1, '1Pre', '2Post'))

#selbal time wet
cv_cyto_prepost <- selbal.cv(x=cyto_untx_t_prepost, y=prepost_var, zero.rep="bayes",seed = 1,col = c("#0072B2","#D55E00"))
```


```
############################################################### 
 STARTING selbal.cv FUNCTION 
###############################################################

#-------------------------------------------------------------# 
# ZERO REPLACEMENT . . .


, . . . FINISHED. 
#-------------------------------------------------------------#

#-------------------------------------------------------------# 
# Starting the cross - validation procedure . . .
```


```
already exporting variable(s): logit.acc
```


```
 . . . finished. 
#-------------------------------------------------------------# 
###############################################################

 The optimal number of variables is: 3
```


```
Setting levels: control = 1Pre, case = 2Post
Setting direction: controls < cases
Setting levels: control = 0, case = 1
Setting direction: controls < cases
```


```
############################################################### 
 . . . FINISHED. 
###############################################################
```


```
cv_cyto_prepost$accuracy.nvar
```


```
cv_cyto_prepost$var.barplot
grid.draw(cv_cyto_prepost$global.plot)
```


```
plot.tab(cv_cyto_prepost$cv.tab)
cv_cyto_prepost$glm
```


```
Call:  glm(formula = numy ~ ., family = f.class, data = U)

Coefficients:
(Intercept)           V1  
     0.1908       0.6121  

Degrees of Freedom: 103 Total (i.e. Null);  102 Residual
Null Deviance:      144.2 
Residual Deviance: 142.9    AIC: 146.9
```


```
#make pretty selbal  cv_bact_depth
png('./files/PT_selbal/2020-09-20_cb_selbal_cv_cyto_prepost.png', width=5000, height=2900, res=400)
```


```
grid.draw(cv_cyto_prepost$global.plot)
dev.off()
```


```
png 
  2
```


```
png('./files/PT_selbal/2020-09-20_b_cb_selbal_cv_cyto_prepost.png', width=2500, height=2500, res=400)
grid.draw(cv_cyto_prepost$global.plot)
```


```
dev.off()
```


```
png 
  2
```


```
#make Pre Post dicotomous variable 
cyto_map_PrePost$PID
```


```
  [1]  1  1  6  6  7  7  8  8  9  9 12 12 13 13 15 15 16 16 17 17 19 19 20 20 22 22 23 23
 [29] 24 24 25 25 32 32 33 33 35 35 38 38 39 39 40 40 43 43 45 45 49 49 50 50 51 51 52 52
 [57] 55 55 56 56 57 57 58 58 60 60 61 61 62 62 63 63 64 64 65 65 67 67 68 68 69 69 70 70
 [85] 71 71 72 72 73 73 75 75 77 77 78 78 81 81 82 82 84 84 85 85 87 87 89 89
```


```
cyto_map_improved_worsened
```


```
cyto_untx_t[as.numeric(gsub('A|B','',rownames(cyto_untx_t)))%in%cyto_map_improved_worsened$PID,]
```


```
cyto_t_prepost <- cyto_t[rownames(cyto_t)%in%rownames(bact_map_improved_worsened),]

##regular Prepost 

#prepost_var <- as.numeric(cyto_map_improved_worsened$PDSumdiff)
var <- as.numeric(bact_map_improved_worsened[rownames(cyto_t_prepost),]$PDsum)


#selbal time wet
cv_cyto_PD <- selbal.cv(x=cyto_t_prepost, y=var, zero.rep="bayes",seed = 1,col = c("#0072B2","#D55E00"))
```


```
############################################################### 
 STARTING selbal.cv FUNCTION 
###############################################################

#-------------------------------------------------------------# 
# ZERO REPLACEMENT . . .


, . . . FINISHED. 
#-------------------------------------------------------------#

#-------------------------------------------------------------# 
# Starting the cross - validation procedure . . .
```


```
already exporting variable(s): logit.acc
```


```
 . . . finished. 
#-------------------------------------------------------------# 
###############################################################

 The optimal number of variables is: 2 


############################################################### 
 . . . FINISHED. 
###############################################################
```


```
cv_cyto_PD$accuracy.nvar
```


```
cv_cyto_PD$var.barplot
grid.draw(cv_cyto_PD$global.plot)
```


```
plot.tab(cv_cyto_PD$cv.tab)
cv_cyto_PD$glm
```


```
Call:  glm(formula = numy ~ ., family = f.class, data = U)

Coefficients:
(Intercept)           V1  
      97.21        27.47  

Degrees of Freedom: 59 Total (i.e. Null);  58 Residual
Null Deviance:      479900 
Residual Deviance: 458200   AIC: 712.7
```


```
#make pretty selbal  cv_bact_depth
png('./files/PT_selbal/2020-09-20_cb_selbal_cv_cyto_PD.png', width=2500, height=2500, res=400)
```


```
grid.draw(cv_cyto_PD$global.plot)
dev.off()
```


```
png 
  2
```


```
##regular impr/wors 
cyto_t_prepost <- cyto_t[rownames(cyto_t)%in%rownames(bact_map_improved_worsened),]
#prepost_var <- as.numeric(cyto_map_improved_worsened$PDSumdiff)
var <- as.factor(bact_map_improved_worsened[rownames(cyto_t_prepost),]$OverallResponse)


#selbal time wet
cv_cyto_OR <- selbal.cv(x=cyto_t_prepost, y=var, zero.rep="bayes",seed = 1,col = c("#0072B2","#D55E00"))
```


```
############################################################### 
 STARTING selbal.cv FUNCTION 
###############################################################

#-------------------------------------------------------------# 
# ZERO REPLACEMENT . . .


, . . . FINISHED. 
#-------------------------------------------------------------#

#-------------------------------------------------------------# 
# Starting the cross - validation procedure . . .
```


```
already exporting variable(s): logit.acc
```


```
 . . . finished. 
#-------------------------------------------------------------# 
###############################################################

 The optimal number of variables is: 2
```


```
Setting levels: control = Improved, case = Worsened
Setting direction: controls < cases
Setting levels: control = 0, case = 1
Setting direction: controls < cases
```


```
############################################################### 
 . . . FINISHED. 
###############################################################
```


```
cv_cyto_OR$accuracy.nvar
```


```
cv_cyto_OR$var.barplot
grid.draw(cv_cyto_OR$global.plot)
```


```
plot.tab(cv_cyto_OR$cv.tab)
cv_cyto_OR$glm
```


```
Call:  glm(formula = numy ~ ., family = f.class, data = U)

Coefficients:
(Intercept)           V1  
    0.04658      0.83525  

Degrees of Freedom: 59 Total (i.e. Null);  58 Residual
Null Deviance:      80.76 
Residual Deviance: 75.65    AIC: 79.65
```


```
#make pretty selbal  cv cyto OR
png('./files/PT_selbal/2020-09-20_cb_selbal_cv_cyto_OR.png', width=5000, height=2900, res=400)
```


```
grid.draw(cv_cyto_OR$global.plot)
dev.off()
```


```
png 
  2
```


```
png('./files/PT_selbal/2020-09-20_b_cb_selbal_cv_cyto_OR.png', width=2500, height=2500, res=400)
grid.draw(cv_cyto_OR$global.plot)
```


```
dev.off()
```


```
png 
  2
```


```
##better pre post 

cyto_map_improved_worsened
```


```
cyto_untx_t[as.numeric(gsub('A|B','',rownames(cyto_untx_t)))%in%cyto_map_improved_worsened$PID,]
```


```
cyto_untx_t_prepost <- cyto_untx_t[as.numeric(gsub('A|B','',rownames(cyto_untx_t)))%in%cyto_map_improved_worsened$PID,]

index_improved <- as.numeric(gsub('A|B|"0','',rownames(cyto_improved)))
prepost_var <- as.factor(cyto_map_improved_worsened[cyto_map_improved_worsened$PID %in% index_improved,]$Case_status)
prepost_var <- as.factor(ifelse(prepost_var == 1, '1Pre', '2Post'))

nrow(cyto_improved)
```


```
[1] 24
```


```
ncol(cyto_improved)
```


```
[1] 8
```


```
length(prepost_var)
```


```
[1] 24
```


```
#selbal better pre post 
cv_cyto_improved_prepost <- selbal.cv(x=cyto_improved, y=prepost_var, zero.rep="bayes",seed = 1,col = c("#0072B2","#D55E00"))
```


```
############################################################### 
 STARTING selbal.cv FUNCTION 
###############################################################

#-------------------------------------------------------------# 
# ZERO REPLACEMENT . . .


, . . . FINISHED. 
#-------------------------------------------------------------#

#-------------------------------------------------------------# 
# Starting the cross - validation procedure . . .
```


```
already exporting variable(s): logit.acc
```


```
 . . . finished. 
#-------------------------------------------------------------# 
###############################################################

 The optimal number of variables is: 2
```


```
Setting levels: control = 1Pre, case = 2Post
Setting direction: controls < cases
Setting levels: control = 0, case = 1
Setting direction: controls < cases
```


```
############################################################### 
 . . . FINISHED. 
###############################################################
```


```
cv_cyto_improved_prepost$accuracy.nvar
```


```
cv_cyto_improved_prepost$var.barplot
grid.draw(cv_cyto_improved_prepost$global.plot)
```


```
plot.tab(cv_cyto_improved_prepost$cv.tab)
cv_cyto_improved_prepost$glm
```


```
Call:  glm(formula = numy ~ ., family = f.class, data = U)

Coefficients:
(Intercept)           V1  
     1.1733       0.7111  

Degrees of Freedom: 23 Total (i.e. Null);  22 Residual
Null Deviance:      33.27 
Residual Deviance: 32.56    AIC: 36.56
```


```
#make pretty selbal  better pre post 
png('./files/PT_selbal/2020-09-20_cb_selbal_cv_cyto_improved_prepost.png', width=5000, height=2900, res=400)
```


```
grid.draw(cv_cyto_improved_prepost$global.plot)
dev.off()
```


```
png 
  2
```


```
png('./files/PT_selbal/2020-09-20_b_cb_selbal_cv_cyto_improved_prepost.png', width=2500, height=2500, res=400)
grid.draw(cv_cyto_improved_prepost$global.plot)
```


```
dev.off()
```


```
png 
  2
```


```
##worse pre post

index_worsened <- as.numeric(gsub('A|B|"0','',rownames(cyto_worsened)))
prepost_var <- as.factor(cyto_map_improved_worsened[cyto_map_improved_worsened$PID %in% index_worsened,]$Case_status)
prepost_var <- as.factor(ifelse(prepost_var == 1, '1Pre', '2Post'))
PDpre_var <- as.numeric(cyto_map_improved_worsened[cyto_map_improved_worsened$PID %in% index_worsened,]$PrePDsum)
PDpost_var <- as.numeric(cyto_map_improved_worsened[cyto_map_improved_worsened$PID %in% index_worsened,]$PostPDsum)


#selbal time wet
cv_cyto_worsened_prepost <- selbal.cv(x=cyto_worsened, y=prepost_var, zero.rep="bayes",seed = 1,col = c("#0072B2","#D55E00"))
```


```
############################################################### 
 STARTING selbal.cv FUNCTION 
###############################################################

#-------------------------------------------------------------# 
# ZERO REPLACEMENT . . .


, . . . FINISHED. 
#-------------------------------------------------------------#

#-------------------------------------------------------------# 
# Starting the cross - validation procedure . . .
```


```
already exporting variable(s): logit.acc
```


```
 . . . finished. 
#-------------------------------------------------------------# 
###############################################################

 The optimal number of variables is: 2
```


```
Setting levels: control = 1Pre, case = 2Post
Setting direction: controls < cases
Setting levels: control = 0, case = 1
Setting direction: controls < cases
```


```
############################################################### 
 . . . FINISHED. 
###############################################################
```


```
cv_cyto_worsened_prepost$accuracy.nvar
```


```
cv_cyto_worsened_prepost$var.barplot
grid.draw(cv_cyto_worsened_prepost$global.plot)
```


```
plot.tab(cv_cyto_worsened_prepost$cv.tab)
cv_cyto_worsened_prepost$glm
```


```
Call:  glm(formula = numy ~ ., family = f.class, data = U)

Coefficients:
(Intercept)           V1  
    0.09873      0.09711  

Degrees of Freedom: 35 Total (i.e. Null);  34 Residual
Null Deviance:      49.91 
Residual Deviance: 49.85    AIC: 53.85
```


```
#make pretty selbal  cv_bact_depth
png('./files/PT_selbal/2020-09-20_cb_selbal_cv_cyto_worsened_prepost.png', width=5000, height=2900, res=400)
```


```
grid.draw(cv_cyto_worsened_prepost$global.plot)
dev.off()
```


```
png 
  2
```


```
png('./files/PT_selbal/2020-09-20_cb_selbal_b_cv_cyto_worsened_prepost.png', width=2500, height=2500, res=400)
grid.draw(cv_cyto_worsened_prepost$global.plot)
```


```
dev.off()
```


```
png 
  2
```


```
##impr PD
index_improved <- as.numeric(gsub('A|B|"0','',rownames(cyto_improved)))
PD_var <- as.numeric(cyto_map_improved_worsened[cyto_map_improved_worsened$PID %in% index_improved,]$PDSum)
#selbal time wet
cv_cyto_improved_PD <- selbal.cv(x=cyto_improved, y=PD_var, zero.rep="bayes",seed = 1,col = c("#0072B2","#D55E00"))
```


```
############################################################### 
 STARTING selbal.cv FUNCTION 
###############################################################

#-------------------------------------------------------------# 
# ZERO REPLACEMENT . . .


, . . . FINISHED. 
#-------------------------------------------------------------#

#-------------------------------------------------------------# 
# Starting the cross - validation procedure . . .
```


```
already exporting variable(s): logit.acc
```


```
 . . . finished. 
#-------------------------------------------------------------# 
###############################################################

 The optimal number of variables is: 2 


############################################################### 
 . . . FINISHED. 
###############################################################
```


```
cv_cyto_improved_PD$accuracy.nvar
```


```
cv_cyto_improved_PD$var.barplot
grid.draw(cv_cyto_improved_PD$global.plot)
```


```
plot.tab(cv_cyto_improved_PD$cv.tab)
cv_cyto_improved_PD$glm
```


```
Call:  glm(formula = numy ~ ., family = f.class, data = U)

Coefficients:
(Intercept)           V1  
      318.1        136.4  

Degrees of Freedom: 23 Total (i.e. Null);  22 Residual
Null Deviance:      261800 
Residual Deviance: 150900   AIC: 284
```


```
#make pretty selbal  cv_depth
png('./files/PT_selbal/2020-09-20_cb_selbal_cv_cyto_improved_PD.png', width=2500, height=2500, res=400)
```


```
grid.draw(cv_cyto_improved_PD$global.plot)
dev.off()
```


```
png 
  2
```


```
##worse PD
index_worsened <- as.numeric(gsub('A|B|"0','',rownames(cyto_worsened)))
PD_var <- as.numeric(cyto_map_improved_worsened[cyto_map_improved_worsened$PID %in% index_worsened,]$PDSum)
#selbal time wet
cv_cyto_worsened_PD <- selbal.cv(x=cyto_worsened, y=PD_var, zero.rep="bayes",seed = 1,col = c("#0072B2","#D55E00"))
```


```
############################################################### 
 STARTING selbal.cv FUNCTION 
###############################################################

#-------------------------------------------------------------# 
# ZERO REPLACEMENT . . .


, . . . FINISHED. 
#-------------------------------------------------------------#

#-------------------------------------------------------------# 
# Starting the cross - validation procedure . . .
```


```
already exporting variable(s): logit.acc
```


```
 . . . finished. 
#-------------------------------------------------------------# 
###############################################################

 The optimal number of variables is: 2 


############################################################### 
 . . . FINISHED. 
###############################################################
```


```
cv_cyto_worsened_PD$accuracy.nvar
```


```
cv_cyto_worsened_PD$var.barplot
grid.draw(cv_cyto_worsened_PD$global.plot)
```


```
plot.tab(cv_cyto_worsened_PD$cv.tab)
cv_cyto_worsened_PD$glm
```


```
Call:  glm(formula = numy ~ ., family = f.class, data = U)

Coefficients:
(Intercept)           V1  
     100.55        23.11  

Degrees of Freedom: 35 Total (i.e. Null);  34 Residual
Null Deviance:      214100 
Residual Deviance: 202000   AIC: 418.9
```


```
#make pretty selbal  cv_depth
png('./files/PT_selbal/2020-09-20_cb_selbal_cv_cyto_worsened_PD.png', width=2500, height=2500, res=400)
```


```
grid.draw(cv_cyto_worsened_PD$global.plot)
dev.off()
```


```
png 
  2
```


```
#metagen prepost

var <- as.factor(metagen_map_ordered$PerioTreatment)
var <- as.factor(ifelse(var == 'Pre', '1Pre', '2Post'))
selbal_t <- metagen_genus_t
rownames(selbal_t) <- gsub('.kraken','',rownames(selbal_t))
nrow(selbal_t)
```


```
[1] 22
```


```
length(var)
```


```
[1] 22
```


```
#selbal time wet
cv_metagen_prepost <- selbal.cv(x=selbal_t, y=var, zero.rep="bayes",seed = 1,col = c("#0072B2","#D55E00"))
```


```
############################################################### 
 STARTING selbal.cv FUNCTION 
###############################################################

#-------------------------------------------------------------# 
# ZERO REPLACEMENT . . .


, . . . FINISHED. 
#-------------------------------------------------------------#

#-------------------------------------------------------------# 
# Starting the cross - validation procedure . . .
```


```
already exporting variable(s): logit.acc
```


```
 . . . finished. 
#-------------------------------------------------------------# 
###############################################################

 The optimal number of variables is: 2
```


```
Setting levels: control = 1Pre, case = 2Post
Setting direction: controls < cases
Setting levels: control = 0, case = 1
Setting direction: controls < cases
```


```
############################################################### 
 . . . FINISHED. 
###############################################################
```


```
cv_metagen_prepost$accuracy.nvar
```


```
cv_metagen_prepost$var.barplot
grid.draw(cv_metagen_prepost$global.plot)
```


```
plot.tab(cv_metagen_prepost$cv.tab)
cv_metagen_prepost$glm
```


```
Call:  glm(formula = numy ~ ., family = f.class, data = U)

Coefficients:
(Intercept)           V1  
      4.584        1.460  

Degrees of Freedom: 21 Total (i.e. Null);  20 Residual
Null Deviance:      30.5 
Residual Deviance: 21.67    AIC: 25.67
```


```
#make pretty selbal  cv_bact_depth
png('./files/PT_selbal/2020-09-20_cb_selbal_cv_metagen_prepost.png', width=5000, height=2900, res=400)
```


```
grid.draw(cv_metagen_prepost$global.plot)
dev.off()
```


```
png 
  2
```


```
png('./files/PT_selbal/2020-09-20_cb_selbal_cv_metagen_prepost.png', width=2500, height=2500, res=400)
grid.draw(cv_metagen_prepost$global.plot)
```


```
dev.off()
```


```
png 
  2
```


```
#metagen OR

var <- as.factor(metagen_map_ordered$OverallResponse)
#var <- as.factor(ifelse(var == 'Pre', '1Pre', '2Post'))
selbal_t <- metagen_genus_t
rownames(selbal_t) <- gsub('.kraken','',rownames(selbal_t))
nrow(selbal_t)
```


```
[1] 22
```


```
length(var)
```


```
[1] 22
```


```
#selbal time wet
cv_metagen_OR <- selbal.cv(x=selbal_t, y=var, zero.rep="bayes",seed = 1,col = c("#0072B2","#D55E00"))
```


```
############################################################### 
 STARTING selbal.cv FUNCTION 
###############################################################

#-------------------------------------------------------------# 
# ZERO REPLACEMENT . . .


, . . . FINISHED. 
#-------------------------------------------------------------#

#-------------------------------------------------------------# 
# Starting the cross - validation procedure . . .
```


```
already exporting variable(s): logit.acc
```


```
 . . . finished. 
#-------------------------------------------------------------# 
###############################################################

 The optimal number of variables is: 2
```


```
Setting levels: control = Improved, case = Worsened
Setting direction: controls < cases
Setting levels: control = 0, case = 1
Setting direction: controls < cases
```


```
############################################################### 
 . . . FINISHED. 
###############################################################
```


```
cv_metagen_OR$accuracy.nvar
```


```
cv_metagen_OR$barplot
```


```
NULL
```


```
grid.draw(cv_metagen_OR$global.plot)
plot.tab(cv_metagen_OR$cv.tab)
```


```
cv_metagen_OR$glm
```


```
Call:  glm(formula = numy ~ ., family = f.class, data = U)

Coefficients:
(Intercept)           V1  
     -4.071        3.273  

Degrees of Freedom: 21 Total (i.e. Null);  20 Residual
Null Deviance:      30.32 
Residual Deviance: 17.8     AIC: 21.8
```


```
#make pretty selbal  cv_
png('./files/PT_selbal/2020-09-20_cb_selbal_cv_metagen_OR.png', width=5000, height=2900, res=400)
grid.draw(cv_metagen_OR$global.plot)
```


```
dev.off()
```


```
png 
  2
```


```
png('./files/PT_selbal/2020-09-20_b_cb_selbal_cv_metagen_OR.png', width=2500, height=2500, res=400)
```


```
grid.draw(cv_metagen_OR$global.plot)
dev.off()
```


```
png 
  2
```


```
#metagen pdsum
var <- as.numeric(bact_map_improved_worsened[gsub('.kraken','',rownames(metagen_genus_t)),]$PDsum)
#var <- as.factor(ifelse(var == 'Pre', '1Pre', '2Post'))
selbal_t <- metagen_genus_t
rownames(selbal_t) <- gsub('.kraken','',rownames(selbal_t))
nrow(selbal_t)
```


```
[1] 22
```


```
length(var)
```


```
[1] 22
```


```
#selbal time wet
cv_metagen_PD <- selbal.cv(x=selbal_t, y=var, zero.rep="bayes",seed = 1,col = c("#0072B2","#D55E00"))
```


```
############################################################### 
 STARTING selbal.cv FUNCTION 
###############################################################

#-------------------------------------------------------------# 
# ZERO REPLACEMENT . . .


, . . . FINISHED. 
#-------------------------------------------------------------#

#-------------------------------------------------------------# 
# Starting the cross - validation procedure . . .
```


```
already exporting variable(s): logit.acc
```


```
 . . . finished. 
#-------------------------------------------------------------# 
###############################################################

 The optimal number of variables is: 2 


############################################################### 
 . . . FINISHED. 
###############################################################
```


```
cv_metagen_PD$accuracy.nvar
```


```
cv_metagen_PD$var.barplot
grid.draw(cv_metagen_PD$global.plot)
```


```
plot.tab(cv_metagen_PD$cv.tab)
cv_metagen_PD$glm
```


```
Call:  glm(formula = numy ~ ., family = f.class, data = U)

Coefficients:
(Intercept)           V1  
     -99.95        97.20  

Degrees of Freedom: 21 Total (i.e. Null);  20 Residual
Null Deviance:      302400 
Residual Deviance: 117400   AIC: 257.2
```


```
#make pretty selbal  cv_
png('./files/PT_selbal/2020-09-20_cb_selbal_cv_metagen_PD.png', width=3500, height=3500, res=400)
```


```
grid.draw(cv_metagen_PD$global.plot)
dev.off()
```


```
png 
  2
```


```
#metagen improved prepost
var <- as.factor(metagen_map_ordered[metagen_map_ordered$OverallResponse=='Improved',]$PerioTreatment)
var <- as.factor(ifelse(var == 'Pre', '1Pre', '2Post'))
selbal_t <- metagen_improved
rownames(selbal_t) <- gsub('.kraken','',rownames(selbal_t))
nrow(selbal_t)
```


```
[1] 10
```


```
length(var)
```


```
[1] 10
```


```
#selbal time wet
cv_metagen_imp_prepost <- selbal.cv(x=selbal_t,n.fold = 3, y=var,seed=1, zero.rep="one",col = c("#0072B2","#D55E00"))
```


```
############################################################### 
 STARTING selbal.cv FUNCTION 
###############################################################

#-------------------------------------------------------------# 
# ZERO REPLACEMENT . . .


, . . . FINISHED. 
#-------------------------------------------------------------#

#-------------------------------------------------------------# 
# Starting the cross - validation procedure . . .
```


```
already exporting variable(s): logit.acc
```


```
 . . . finished. 
#-------------------------------------------------------------# 
###############################################################

 The optimal number of variables is: 2
```


```
glm.fit: algorithm did not convergeglm.fit: fitted probabilities numerically 0 or 1 occurredglm.fit: algorithm did not convergeglm.fit: fitted probabilities numerically 0 or 1 occurredglm.fit: fitted probabilities numerically 0 or 1 occurredglm.fit: algorithm did not convergeglm.fit: fitted probabilities numerically 0 or 1 occurredglm.fit: algorithm did not convergeglm.fit: fitted probabilities numerically 0 or 1 occurredglm.fit: fitted probabilities numerically 0 or 1 occurredglm.fit: fitted probabilities numerically 0 or 1 occurredglm.fit: algorithm did not convergeglm.fit: fitted probabilities numerically 0 or 1 occurredglm.fit: algorithm did not convergeglm.fit: fitted probabilities numerically 0 or 1 occurredglm.fit: algorithm did not convergeglm.fit: fitted probabilities numerically 0 or 1 occurredglm.fit: algorithm did not convergeglm.fit: fitted probabilities numerically 0 or 1 occurredglm.fit: algorithm did not convergeglm.fit: fitted probabilities numerically 0 or 1 occurredglm.fit: algorithm did not convergeglm.fit: fitted probabilities numerically 0 or 1 occurredglm.fit: algorithm did not convergeglm.fit: fitted probabilities numerically 0 or 1 occurredglm.fit: algorithm did not convergeglm.fit: fitted probabilities numerically 0 or 1 occurredglm.fit: algorithm did not convergeglm.fit: fitted probabilities numerically 0 or 1 occurredSetting levels: control = 1Pre, case = 2Post
Setting direction: controls < cases
Setting levels: control = 0, case = 1
Setting direction: controls < cases
```


```
############################################################### 
 . . . FINISHED. 
###############################################################
```


```
cv_metagen_imp_prepost$accuracy.nvar
```


```
cv_metagen_imp_prepost$var.barplot
grid.draw(cv_metagen_imp_prepost$global.plot)
```


```
plot.tab(cv_metagen_imp_prepost$cv.tab)
cv_metagen_imp_prepost$glm
```


```
Call:  glm(formula = numy ~ ., family = f.class, data = U)

Coefficients:
(Intercept)           V1  
      60.78       229.43  

Degrees of Freedom: 9 Total (i.e. Null);  8 Residual
Null Deviance:      13.86 
Residual Deviance: 1.317e-09    AIC: 4
```


```
#make pretty selbal  cv_
png('./files/PT_selbal/2020-09-20_cb_selbal_cv_metagen_improved_prepost.png', width=5000, height=2900, res=400)
```


```
grid.draw(cv_metagen_imp_prepost$global.plot)
dev.off()
```


```
png 
  2
```


```
#make pretty selbal  cv_
png('./files/PT_selbal/2020-09-20_b_cb_selbal_cv_metagen_improved_prepost.png', width=2500, height=2500, res=400)
grid.draw(cv_metagen_imp_prepost$global.plot)
```


```
dev.off()
```


```
png 
  2
```


```
#metagen worsened prepost
var <- as.factor(metagen_map_ordered[metagen_map_ordered$OverallResponse=='Worsened',]$PerioTreatment)
var <- as.factor(ifelse(var == 'Pre', '1Pre', '2Post'))
selbal_t <- metagen_worsened
rownames(selbal_t) <- gsub('.kraken','',rownames(selbal_t))
nrow(selbal_t)
```


```
[1] 12
```


```
length(var)
```


```
[1] 12
```


```
#selbal time wet
cv_metagen_wor_prepost <- selbal.cv(x=selbal_t, y=var, zero.rep="one",seed = 1,col = c("#0072B2","#D55E00"))
```


```
############################################################### 
 STARTING selbal.cv FUNCTION 
###############################################################

#-------------------------------------------------------------# 
# ZERO REPLACEMENT . . .


, . . . FINISHED. 
#-------------------------------------------------------------#

#-------------------------------------------------------------# 
# Starting the cross - validation procedure . . .
```


```
already exporting variable(s): logit.acc
```


```
 . . . finished. 
#-------------------------------------------------------------# 
###############################################################

 The optimal number of variables is: 2
```


```
glm.fit: fitted probabilities numerically 0 or 1 occurredglm.fit: fitted probabilities numerically 0 or 1 occurredglm.fit: fitted probabilities numerically 0 or 1 occurredglm.fit: fitted probabilities numerically 0 or 1 occurredSetting levels: control = 1Pre, case = 2Post
Setting direction: controls < cases
Setting levels: control = 0, case = 1
Setting direction: controls < cases
```


```
############################################################### 
 . . . FINISHED. 
###############################################################
```


```
cv_metagen_wor_prepost$accuracy.nvar
```


```
cv_metagen_wor_prepost$var.barplot
grid.draw(cv_metagen_wor_prepost$global.plot)
```


```
plot.tab(cv_metagen_wor_prepost$cv.tab)
cv_metagen_wor_prepost$glm
```


```
Call:  glm(formula = numy ~ ., family = f.class, data = U)

Coefficients:
(Intercept)           V1  
     194.42        67.61  

Degrees of Freedom: 11 Total (i.e. Null);  10 Residual
Null Deviance:      16.64 
Residual Deviance: 3.097e-10    AIC: 4
```


```
#make pretty selbal  cv_
png('./files/PT_selbal/2020-09-20_cb_selbal_cv_metagen_worsened_prepost.png', width=5000, height=2900, res=400)
```


```
grid.draw(cv_metagen_wor_prepost$global.plot)
dev.off()
```


```
png 
  2
```


```
png('./files/PT_selbal/2020-09-20_b_cb_selbal_cv_metagen_worsened_prepost.png', width=2500, height=2500, res=400)
grid.draw(cv_metagen_wor_prepost$global.plot)
```


```
dev.off()
```


```
png 
  2
```


```
#metagen improved PD
#metagen_genus_t
var <- as.numeric(bact_map_improved_worsened[gsub('.kraken','',rownames(metagen_improved)),]$PDsum)
# var <- as.factor(ifelse(var == 'Pre', '1Pre', '2Post'))
selbal_t <- metagen_improved
rownames(selbal_t) <- gsub('.kraken','',rownames(selbal_t))
nrow(selbal_t)
```


```
[1] 10
```


```
length(var)
```


```
[1] 10
```


```
#selbal time wet
cv_metagen_imp_PD <- selbal.cv(x=selbal_t,n.fold = 3, y=var, zero.rep="one",seed = 1,col = c("#0072B2","#D55E00"))
```


```
############################################################### 
 STARTING selbal.cv FUNCTION 
###############################################################

#-------------------------------------------------------------# 
# ZERO REPLACEMENT . . .


, . . . FINISHED. 
#-------------------------------------------------------------#

#-------------------------------------------------------------# 
# Starting the cross - validation procedure . . .
```


```
already exporting variable(s): logit.acc
```


```
 . . . finished. 
#-------------------------------------------------------------# 
###############################################################

 The optimal number of variables is: 2 


############################################################### 
 . . . FINISHED. 
###############################################################
```


```
cv_metagen_imp_PD$accuracy.nvar
```


```
cv_metagen_imp_PD$var.barplot
grid.draw(cv_metagen_imp_PD$global.plot)
```


```
plot.tab(cv_metagen_imp_PD$cv.tab)
cv_metagen_imp_PD$glm
```


```
Call:  glm(formula = numy ~ ., family = f.class, data = U)

Coefficients:
(Intercept)           V1  
     176.57        92.79  

Degrees of Freedom: 9 Total (i.e. Null);  8 Residual
Null Deviance:      205000 
Residual Deviance: 20870    AIC: 110.8
```


```
#make pretty selbal  cv_

png('./files/PT_selbal/2020-09-20_cb_selbal_cv_metagen_improved_PD.png', width=2500, height=2500, res=400)
```


```
grid.draw(cv_metagen_imp_PD$global.plot)
dev.off()
```


```
png 
  2
```


```
#metagen worsened PD
#metagen_genus_t
var <- as.numeric(bact_map_improved_worsened[gsub('.kraken','',rownames(metagen_worsened)),]$PDsum)
# var <- as.factor(ifelse(var == 'Pre', '1Pre', '2Post'))
selbal_t <- metagen_worsened
rownames(selbal_t) <- gsub('.kraken','',rownames(selbal_t))
nrow(selbal_t)
```


```
[1] 12
```


```
length(var)
```


```
[1] 12
```


```
#selbal time wet
cv_metagen_wor_PD <- selbal.cv(x=selbal_t,n.fold = 3, y=var,seed=1, zero.rep="one",col = c("#0072B2","#D55E00"))
```


```
############################################################### 
 STARTING selbal.cv FUNCTION 
###############################################################

#-------------------------------------------------------------# 
# ZERO REPLACEMENT . . .


, . . . FINISHED. 
#-------------------------------------------------------------#

#-------------------------------------------------------------# 
# Starting the cross - validation procedure . . .
```


```
already exporting variable(s): logit.acc
```


```
 . . . finished. 
#-------------------------------------------------------------# 
###############################################################

 The optimal number of variables is: 2 


############################################################### 
 . . . FINISHED. 
###############################################################
```


```
cv_metagen_wor_PD$accuracy.nvar
```


```
cv_metagen_wor_PD$var.barplot
grid.draw(cv_metagen_wor_PD$global.plot)
```


```
plot.tab(cv_metagen_wor_PD$cv.tab)
cv_metagen_wor_PD$glm
```


```
Call:  glm(formula = numy ~ ., family = f.class, data = U)

Coefficients:
(Intercept)           V1  
      708.1        135.3  

Degrees of Freedom: 11 Total (i.e. Null);  10 Residual
Null Deviance:      89320 
Residual Deviance: 23620    AIC: 131.1
```


```
#make pretty selbal  cv_
png('./files/PT_selbal/2020-09-20_cb_selbal_cv_metagen_worsened_PDsum.png', width=2500, height=2500, res=400)
```


```
grid.draw(cv_metagen_wor_PD$global.plot)
dev.off()
```


```
png 
  2
```


SHT selbal analysis


```
#SHT data read in 
#read in data
bact_df_ordered <- read.table('./files/2020-09-20_SHT_16S_OTU_genus_Tabl.csv',header = 1,row.names = 1,sep=',',check.names=FALSE)
bact_genus10_redslim <-bact_df_ordered
metab_df <- read.table('./files/2020-09-20_SHT_metab_table.csv',sep=',',header =1,row.names = 1,check.names = F,na.strings = 'ND')
metab_untx_red <- metab_df[rowSums(metab_df <1000000) <= 0.90 * ncol(metab_df), ]
metagen_df_ordered <- read.table('./files/2020-09-20_SHT_metagen_genus_table.csv',sep=',',row.names = 1,header = 1,check.names=FALSE)
metagen_genus_red <- metagen_df_ordered
```


```
#import sample maps
bact_map_ordered <- read.table('./files/2020-08-11_SHT_mappingfile.csv',sep=',',header =1,row.names = 1,check.names = F)

metagen_map<- read.table('./files/Metadata_and_perio_NA.txt',sep='\t',header =1,row.names = 1)
metagen_map_ordered <- metagen_map[order(row.names(metagen_map)),]
```


```
# #didease class 
# #pocket depth 

###which samples to keep  #del PA and NA pocketdepth
bact_map_noPa <- bact_map_ordered[grep( 'B|b',row.names(bact_map_ordered)),]
# bact_map_noPa_ABC <- bact_map_noPa[grep('A|B|C',bact_map_noPa$diseasclass),]
bact_df_noPa <- bact_df_ordered[,grep('B|b',colnames(bact_df_ordered))]
bact_map_noPa <- bact_map_ordered[rownames(bact_map_ordered) %in% colnames(bact_df_noPa),]
bact_map_noPa <- bact_map_noPa[!is.na(as.numeric(bact_map_noPa$pocketdepth)),]
```


```
NAs introduced by coercion
```


```
bact_map_noPa_AC <- bact_map_noPa[grep('A|C',bact_map_noPa$diseasclass),]
bact_df_noPa_AC <- bact_df_noPa[,bact_map_noPa_AC$Description]

metab_noPa <- metab_df[,grep('b|B',colnames(metab_df))]
metab_map_noPa <- bact_map_ordered[bact_map_ordered$metab_description %in% colnames(metab_noPa),]#
metab_map_noPa <- metab_map_noPa[!is.na(as.numeric(metab_map_noPa$pocketdepth)),]
```


```
NAs introduced by coercion
```


```
metab_map_noPa_AC <- metab_map_noPa[grep('A|C',metab_map_noPa$diseasclass),]
# metab_noPa_AC <- metab_noPa[,colnames(metab_noPa)%in%rownames(metab_map_noPa_AC)]

# setup
#filter metagen
# metagen_df_filtered <- metagen_df_ordered[,colnames(metagen_df_ordered)%in%row.names(metagen_map_ordered)] 
metagen_map_noPa <- metagen_map_ordered[grep('B|b',row.names(metagen_map_ordered)),]
metagen_map_noPa_AC <- metagen_map_noPa[grep('A|C',metagen_map_noPa$DiseasClass),]
metagen_noPa_AC <- metagen_df_filtered[,colnames(metagen_df_filtered)%in%rownames(metagen_map_noPa_AC)[grep('B|b',rownames(metagen_map_noPa_AC))]]
```


```
######SELBAL
# metagen_untx_sum
# bact_genus10_redslim
# metab_untx_red
# # map_untx
# # metab_untx_redb
# metagen_genus_red

###which samples to keep  #del PA based on map used on all analysis
# bact_map_noPa<-bact_map_ordered[grep( 'B|b',row.names(bact_map_ordered)),]
bact_df_noPa <- bact_genus10_redslim[,grep('B|b',colnames(bact_genus10_redslim))]
bact_df_noPa_AC <- bact_df_noPa[,bact_map_noPa_AC$Description]

metab_noPa <- metab_untx_red[,grep('b|B',colnames(metab_untx_red))]
metab_noPa_AC <- metab_noPa[,gsub('B','.B',colnames(metab_noPa))%in%rownames(metab_map_noPa_AC)]

#filter metagen
metagen_df_filtered <- metagen_genus_red[,row.names(metagen_map_noPa_AC)] 
metagen_map_noPa_AC <- metagen_map_noPa_AC[grep('B|b',row.names(metagen_map_noPa_AC)),]
metagen_noPa <- metagen_df_filtered[,grep('B|b',colnames(metagen_df_filtered))]
```


```
#bact selbal PC A C
bact_map_noPa_AC <- bact_map_noPa_AC[grep('A|C',bact_map_noPa_AC$diseasclass),]
bact_df_noPa_AC <-bact_df_noPa_AC[,bact_map_noPa_AC$Description]

var_bact <- as.factor(bact_map_noPa_AC$diseasclass)

#selbal needs two inputs- matrix with samples as rows and taxa as column
#setup 
bact_genus_sum_t <- t(bact_df_noPa_AC)#

cv_bact_AvC_DC <- selbal.cv(x=bact_genus_sum_t, y=var_bact, zero.rep="bayes",seed = 1,col = c("#0072B2","#D55E00"))
```


```
############################################################### 
 STARTING selbal.cv FUNCTION 
###############################################################

#-------------------------------------------------------------# 
# ZERO REPLACEMENT . . .


, . . . FINISHED. 
#-------------------------------------------------------------#

#-------------------------------------------------------------# 
# Starting the cross - validation procedure . . .
```


```
already exporting variable(s): logit.acc
```


```
 . . . finished. 
#-------------------------------------------------------------# 
###############################################################

 The optimal number of variables is: 5
```


```
Setting levels: control = A, case = C
Setting direction: controls < cases
Setting levels: control = 0, case = 1
Setting direction: controls < cases
```


```
############################################################### 
 . . . FINISHED. 
###############################################################
```


```
cv_bact_AvC_DC$accuracy.nvar
```


```
cv_bact_AvC_DC$var.barplot
grid.draw(cv_bact_AvC_DC$global.plot)
```


```
plot.tab(cv_bact_AvC_DC$cv.tab)
cv_bact_AvC_DC$glm
```


```
Call:  glm(formula = numy ~ ., family = f.class, data = U)

Coefficients:
(Intercept)           V1  
     0.8484       0.7647  

Degrees of Freedom: 194 Total (i.e. Null);  193 Residual
Null Deviance:      252.2 
Residual Deviance: 185.8    AIC: 189.8
```


```
#make pretty selbal
png('./files/SHT_selbal/2020-09-20_cb_selbal_cv_bact_DC_AC.png', width=5000, height=2900, res=400)
```


```
grid.draw(cv_bact_AvC_DC$global.plot)
dev.off()
```


```
png 
  2
```


```
png('./files/SHT_selbal/2020-09-20_b_cb_selbal_cv_bact_DC_AC.png', width=2500, height=2500, res=333)
grid.draw(cv_bact_AvC_DC$global.plot)
```


```
dev.off()
```


```
png 
  2
```


```
#bact selbal PC AC pocket depth
bact_map_noPa_C <- bact_map_noPa_AC[grep('C',bact_map_noPa_AC$diseasclass),]
bact_df_noPa_C <-bact_df_noPa_AC[,rownames(bact_map_noPa_C)]

var_bact <- as.numeric(bact_map_noPa_C$pocketdepth)
bact_df_noPa_C <- bact_df_noPa_C[,!is.na(var_bact)]
var_bact <- var_bact[!is.na(var_bact)]
#selbal needs two inputs- matrix with samples as rows and taxa as column
#setup 
bact_genus_sum_t <- t(bact_df_noPa_C)#
table(bact_genus_sum_t==0)
```


```
FALSE  TRUE 
 2818   990
```


```
cv_bact_PD_C <- selbal.cv(x=bact_genus_sum_t, y=var_bact, zero.rep="one",seed = 1,col = c("#0072B2","#D55E00"))
```


```
############################################################### 
 STARTING selbal.cv FUNCTION 
###############################################################

#-------------------------------------------------------------# 
# ZERO REPLACEMENT . . .


, . . . FINISHED. 
#-------------------------------------------------------------#

#-------------------------------------------------------------# 
# Starting the cross - validation procedure . . .
```


```
already exporting variable(s): logit.acc
```


```
 . . . finished. 
#-------------------------------------------------------------# 
###############################################################

 The optimal number of variables is: 2 


############################################################### 
 . . . FINISHED. 
###############################################################
```


```
cv_bact_PD_C$accuracy.nvar
```


```
cv_bact_PD_C$var.barplot
grid.draw(cv_bact_PD_C$global.plot)
```


```
plot.tab(cv_bact_PD_C$cv.tab)
cv_bact_PD_C$glm
```


```
Call:  glm(formula = numy ~ ., family = f.class, data = U)

Coefficients:
(Intercept)           V1  
     5.6166       0.5133  

Degrees of Freedom: 67 Total (i.e. Null);  66 Residual
Null Deviance:      431.1 
Residual Deviance: 343.6    AIC: 309.1
```


```
#make pretty selbal  cv_
png('./files/SHT_selbal/2020-09-20_cb_selbal_cv_bact_PD_C.png', width=2500, height=2500, res=400)
```


```
grid.draw(cv_bact_PD_C$global.plot)
dev.off()
```


```
png 
  2
```


```
#bact selbal PC A pocket depth
bact_map_noPa_A <- bact_map_noPa[grep('A',bact_map_noPa$diseasclass),]
bact_df_noPa_A <-bact_df_noPa[,rownames(bact_map_noPa_A)]

var_bact <- as.numeric(bact_map_noPa_A$pocketdepth)
bact_df_noPa_A <- bact_df_noPa_A[,!is.na(var_bact)]
var_bact <- var_bact[!is.na(var_bact)]
#selbal needs two inputs- matrix with samples as rows and taxa as column
#setup 
bact_genus_sum_t <- t(bact_df_noPa_A)#
table(bact_genus_sum_t==0)
```


```
FALSE  TRUE 
 4896  2216
```


```
#selbal pre resp
cv_bact_PD_A <- selbal.cv(x=bact_genus_sum_t, y=var_bact, zero.rep="one",seed = 1,col = c("#0072B2","#D55E00"))
```


```
############################################################### 
 STARTING selbal.cv FUNCTION 
###############################################################

#-------------------------------------------------------------# 
# ZERO REPLACEMENT . . .


, . . . FINISHED. 
#-------------------------------------------------------------#

#-------------------------------------------------------------# 
# Starting the cross - validation procedure . . .
```


```
already exporting variable(s): logit.acc
```


```
 . . . finished. 
#-------------------------------------------------------------# 
###############################################################

 The optimal number of variables is: 2 


############################################################### 
 . . . FINISHED. 
###############################################################
```


```
cv_bact_PD_A$accuracy.nvar
```


```
cv_bact_PD_A$var.barplot
grid.draw(cv_bact_PD_A$global.plot)
```


```
plot.tab(cv_bact_PD_A$cv.tab)
cv_bact_PD_A$glm
```


```
Call:  glm(formula = numy ~ ., family = f.class, data = U)

Coefficients:
(Intercept)           V1  
     4.6928       0.1325  

Degrees of Freedom: 126 Total (i.e. Null);  125 Residual
Null Deviance:      152.3 
Residual Deviance: 146.5    AIC: 384.5
```


```
#make pretty selbal  cv_
png('./files/SHT_selbal/2020-09-20_cb_selbal_cv_bact_PD_A.png', width=2500, height=2500, res=400)
```


```
grid.draw(cv_bact_PD_A$global.plot)
dev.off()
```


```
png 
  2
```


```
#metab selbal A C
metab_map_noPa_AC<- metab_map_noPa_AC[grep('A|C',metab_map_noPa_AC$diseasclass),]
metab_noPa_AC <- metab_noPa_AC[,gsub('.B','B',rownames(metab_map_noPa_AC))]

var_metab <- as.factor(metab_map_noPa_AC$diseasclass)

#selbal needs two inputs- matrix with samples as rows and taxa as column
#setup 
metab_genus_sum_t <- t(metab_noPa_AC)#

#selbal pre resp
cv_metab_AvC_DC <- selbal.cv(x=metab_genus_sum_t, y=var_metab, zero.rep="bayes",seed = 1,col = c("#0072B2","#D55E00"))
```


```
############################################################### 
 STARTING selbal.cv FUNCTION 
###############################################################

#-------------------------------------------------------------# 
# ZERO REPLACEMENT . . .


, . . . FINISHED. 
#-------------------------------------------------------------#

#-------------------------------------------------------------# 
# Starting the cross - validation procedure . . .
```


```
already exporting variable(s): logit.acc
```


```
 . . . finished. 
#-------------------------------------------------------------# 
###############################################################

 The optimal number of variables is: 3
```


```
Setting levels: control = A, case = C
Setting direction: controls < cases
Setting levels: control = 0, case = 1
Setting direction: controls < cases
```


```
############################################################### 
 . . . FINISHED. 
###############################################################
```


```
cv_metab_AvC_DC$accuracy.nvar
```


```
cv_metab_AvC_DC$var.barplot
grid.draw(cv_metab_AvC_DC$global.plot)
```


```
plot.tab(cv_metab_AvC_DC$cv.tab)
cv_metab_AvC_DC$glm
```


```
Call:  glm(formula = numy ~ ., family = f.class, data = U)

Coefficients:
(Intercept)           V1  
    -0.7656       0.6167  

Degrees of Freedom: 138 Total (i.e. Null);  137 Residual
Null Deviance:      186.6 
Residual Deviance: 161.4    AIC: 165.4
```


```
#make pretty selbal  cv_bact_resp
png('./files/SHT_selbal/2020-09-20_cb_selbal_cv_metab_DC_AC.png', width=5000, height=2900, res=400)
```


```
grid.draw(cv_metab_AvC_DC$global.plot)
dev.off()
```


```
png 
  2
```


```
png('./files/SHT_selbal/2020-09-20_b_cb_selbal_cv_metab_DC_AC.png', width=2500, height=2500, res=400)
grid.draw(cv_metab_AvC_DC$global.plot)
```


```
dev.off()
```


```
png 
  2
```


```
#metab selbal A PD
metab_map_noPa_A_PD<- metab_map_noPa_AC[grep('A',metab_map_noPa_AC$diseasclass),]
metab_noPa_A <- metab_noPa_AC[,gsub('.B','B',rownames(metab_map_noPa_A_PD))]

var_metab <- as.numeric(metab_map_noPa_A_PD$pocketdepth)
metab_noPa_A <- metab_noPa_A[,!is.na(var_metab)]
var_metab <- var_metab[!is.na(var_metab)]
#selbal needs two inputs- matrix with samples as rows and taxa as column
#setup 
metab_genus_sum_t <- t(metab_noPa_A)#

#selbal pre resp
cv_metab_A_PD <- selbal.cv(x=metab_genus_sum_t, y=var_metab, zero.rep="bayes",seed = 1,col = c("#0072B2","#D55E00"))
```


```
############################################################### 
 STARTING selbal.cv FUNCTION 
###############################################################

#-------------------------------------------------------------# 
# ZERO REPLACEMENT . . .


, . . . FINISHED. 
#-------------------------------------------------------------#

#-------------------------------------------------------------# 
# Starting the cross - validation procedure . . .
```


```
already exporting variable(s): logit.acc
```


```
 . . . finished. 
#-------------------------------------------------------------# 
###############################################################

 The optimal number of variables is: 2 


############################################################### 
 . . . FINISHED. 
###############################################################
```


```
cv_metab_A_PD$accuracy.nvar
```


```
cv_metab_A_PD$var.barplot
grid.draw(cv_metab_A_PD$global.plot)
```


```
plot.tab(cv_metab_A_PD$cv.tab)
cv_metab_A_PD$glm
```


```
Call:  glm(formula = numy ~ ., family = f.class, data = U)

Coefficients:
(Intercept)           V1  
     4.6797       0.7161  

Degrees of Freedom: 83 Total (i.e. Null);  82 Residual
Null Deviance:      90.95 
Residual Deviance: 79.42    AIC: 239.7
```


```
#make pretty selbal  cv_bact_resp
png('./files/SHT_selbal/2020-09-20_cb_selbal_cv_metab_PD_A.png', width=2500, height=2500, res=400)
```


```
grid.draw(cv_metab_A_PD$global.plot)
dev.off()
```


```
png 
  2
```


```
#metab selbal C PD
metab_map_noPa_C_PD<- metab_map_noPa_AC[grep('C',metab_map_noPa_AC$diseasclass),]
metab_noPa_C <- metab_noPa_AC[,gsub('.B','B',rownames(metab_map_noPa_C_PD))]

#selbal needs two inputs- matrix with samples as rows and taxa as column
#setup 
var_metab <- as.numeric(metab_map_noPa_C_PD$pocketdepth)
metab_noPa_C <- metab_noPa_C[,!is.na(var_metab)]
var_metab <- var_metab[!is.na(var_metab)]
metab_genus_sum_t <- t(metab_noPa_C)#

#selbal pre resp
cv_metab_C_PD <- selbal.cv(x=metab_genus_sum_t, y=var_metab, zero.rep="bayes",seed = 1,col = c("#0072B2","#D55E00"))
```


```
############################################################### 
 STARTING selbal.cv FUNCTION 
###############################################################

#-------------------------------------------------------------# 
# ZERO REPLACEMENT . . .


, . . . FINISHED. 
#-------------------------------------------------------------#

#-------------------------------------------------------------# 
# Starting the cross - validation procedure . . .
```


```
already exporting variable(s): logit.acc
```


```
 . . . finished. 
#-------------------------------------------------------------# 
###############################################################

 The optimal number of variables is: 2 


############################################################### 
 . . . FINISHED. 
###############################################################
```


```
cv_metab_C_PD$accuracy.nvar
```


```
cv_metab_C_PD$var.barplot
grid.draw(cv_metab_C_PD$global.plot)
```


```
plot.tab(cv_metab_C_PD$cv.tab)
cv_metab_C_PD$glm
```


```
Call:  glm(formula = numy ~ ., family = f.class, data = U)

Coefficients:
(Intercept)           V1  
     5.7532       0.4191  

Degrees of Freedom: 54 Total (i.e. Null);  53 Residual
Null Deviance:      319.7 
Residual Deviance: 235.4    AIC: 242
```


```
#make pretty selbal  cv_bact_resp
png('./files/SHT_selbal/2020-09-20_cb_selbal_cv_metab_PD_C.png', width=2500, height=2500, res=400)
```


```
grid.draw(cv_metab_C_PD$global.plot)
dev.off()
```


```
png 
  2
```


```
#metagen selbal A C
table(metagen_noPa>10)
```


```
TRUE 
1240
```


```
metagen_map_noPa_AC #
```


```
metagen_df_noPa_AC <-metagen_noPa[,rownames(metagen_map_noPa_AC)]
rownames(metagen_df_noPa_AC) <-gsub('.*g__','',rownames(metagen_df_noPa_AC))
metagen_df_noPa_AC
```


```
var_metagen <- as.factor(metagen_map_noPa_AC$DiseasClass)

#selbal needs two inputs- matrix with samples as rows and taxa as column
#setup 
metagen_genus_sum_t <- t(metagen_df_noPa_AC)#
table(metagen_genus_sum_t ==0)
```


```
FALSE 
 1240
```


```
#selbal pre resp
cv_metagen_AvC_DC <- selbal.cv(x=metagen_genus_sum_t, y=var_metagen, zero.rep="bayes",seed = 1,col = c("#0072B2","#D55E00"))
```


```
############################################################### 
 STARTING selbal.cv FUNCTION 
###############################################################

#-------------------------------------------------------------# 
# ZERO REPLACEMENT . . .


, . . . FINISHED. 
#-------------------------------------------------------------#

#-------------------------------------------------------------# 
# Starting the cross - validation procedure . . .
```


```
already exporting variable(s): logit.acc
```


```
 . . . finished. 
#-------------------------------------------------------------# 
###############################################################

 The optimal number of variables is: 2
```


```
Setting levels: control = A, case = C
Setting direction: controls < cases
Setting levels: control = 0, case = 1
Setting direction: controls < cases
```


```
############################################################### 
 . . . FINISHED. 
###############################################################
```


```
cv_metagen_AvC_DC$accuracy.nvar
```


```
cv_metagen_AvC_DC$var.barplot
grid.draw(cv_metagen_AvC_DC$global.plot)
```


```
plot.tab(cv_metagen_AvC_DC$cv.tab)
cv_metagen_AvC_DC$glm
```


```
Call:  glm(formula = numy ~ ., family = f.class, data = U)

Coefficients:
(Intercept)           V1  
    -0.1512       1.5458  

Degrees of Freedom: 19 Total (i.e. Null);  18 Residual
Null Deviance:      27.73 
Residual Deviance: 21.31    AIC: 25.31
```


```
#make pretty selbal  cv_bact_resp
png('./files/SHT_selbal/2020-09-20_cb_selbal_cv_metagen_DC_AC.png', width=5000, height=2900, res=400)
```


```
grid.draw(cv_metagen_AvC_DC$global.plot)
dev.off()
```


```
png 
  2
```


```
png('./files/SHT_selbal/2020-09-20_b_cb_selbal_cv_metagen_DC_AC.png', width=2500, height=2500, res=400)
grid.draw(cv_metagen_AvC_DC$global.plot)
```


```
dev.off()
```


```
png 
  2
```


```
#metagen selbal A PD
metagen_map_noPa_AC
```


```
table(metagen_noPa>10)
```


```
TRUE 
1240
```


```
metagen_map_noPa_A <- metagen_map_noPa_AC[grep('A',metagen_map_noPa_AC$DiseasClass),]
metagen_df_noPa_A <-metagen_noPa[,rownames(metagen_map_noPa_A)]
rownames(metagen_df_noPa_A) <-gsub('.*g__','',rownames(metagen_df_noPa_A))
metagen_df_noPa_A
```


```
var_metagen <- as.numeric(metagen_map_noPa_A$PocketDepth)

#selbal needs two inputs- matrix with samples as rows and taxa as column
#setup 
metagen_genus_sum_t <- t(metagen_df_noPa_A)#
table(metagen_genus_sum_t ==0)
```


```
FALSE 
  620
```


```
#selbal pre resp
cv_metagen_A_PD <- selbal.cv(x=metagen_genus_sum_t, y=var_metagen, zero.rep="bayes",seed = 1,col = c("#0072B2","#D55E00"))
```


```
############################################################### 
 STARTING selbal.cv FUNCTION 
###############################################################

#-------------------------------------------------------------# 
# ZERO REPLACEMENT . . .


, . . . FINISHED. 
#-------------------------------------------------------------#

#-------------------------------------------------------------# 
# Starting the cross - validation procedure . . .
```


```
already exporting variable(s): logit.acc
```


```
 . . . finished. 
#-------------------------------------------------------------# 
###############################################################

 The optimal number of variables is: 3 


############################################################### 
 . . . FINISHED. 
###############################################################
```


```
cv_metagen_A_PD$accuracy.nvar
```


```
cv_metagen_A_PD$var.barplot
grid.draw(cv_metagen_A_PD$global.plot)
```


```
plot.tab(cv_metagen_A_PD$cv.tab)
cv_metagen_A_PD$glm
```


```
Call:  glm(formula = numy ~ ., family = f.class, data = U)

Coefficients:
(Intercept)           V1  
     -0.252        1.567  

Degrees of Freedom: 9 Total (i.e. Null);  8 Residual
Null Deviance:      13.6 
Residual Deviance: 1.474    AIC: 15.23
```


```
#make pretty selbal  cv_bact_resp
png('./files/SHT_selbal/2020-09-20_selbal_cv_metagen_PD_A.png', width=2500, height=2500, res=400)
```


```
grid.draw(cv_metagen_A_PD$global.plot)
dev.off()
```


```
png 
  2
```


```
#metagen selbal AC PD
metagen_map_noPa
```


```
table(metagen_noPa>10)
```


```
TRUE 
1240
```


```
metagen_map_noPa_AC <- metagen_map_noPa_AC[grep('A|C',metagen_map_noPa_AC$DiseasClass),]
metagen_df_noPa_AC <-metagen_noPa[,rownames(metagen_map_noPa_AC)]
rownames(metagen_df_noPa_AC) <-gsub('.*g__','',rownames(metagen_df_noPa_AC))
metagen_df_noPa_AC
```


```
var_metagen <- as.numeric(metagen_map_noPa_AC$PocketDepth)

#selbal needs two inputs- matrix with samples as rows and taxa as column
#setup 
metagen_genus_sum_t <- t(metagen_df_noPa_AC)#
table(metagen_genus_sum_t ==0)
```


```
FALSE 
 1240
```


```
#selbal pre resp
cv_metagen_AC_PD <- selbal.cv(x=metagen_genus_sum_t, y=var_metagen, zero.rep="bayes",seed = 1,col = c("#0072B2","#D55E00"))
```


```
############################################################### 
 STARTING selbal.cv FUNCTION 
###############################################################

#-------------------------------------------------------------# 
# ZERO REPLACEMENT . . .


, . . . FINISHED. 
#-------------------------------------------------------------#

#-------------------------------------------------------------# 
# Starting the cross - validation procedure . . .
```


```
already exporting variable(s): logit.acc
```


```
 . . . finished. 
#-------------------------------------------------------------# 
###############################################################

 The optimal number of variables is: 4 


############################################################### 
 . . . FINISHED. 
###############################################################
```


```
cv_metagen_AC_PD$accuracy.nvar
```


```
cv_metagen_AC_PD$var.barplot
grid.draw(cv_metagen_AC_PD$global.plot)
```


```
plot.tab(cv_metagen_AC_PD$cv.tab)
cv_metagen_AC_PD$glm
```


```
Call:  glm(formula = numy ~ ., family = f.class, data = U)

Coefficients:
(Intercept)           V1  
      9.721        1.984  

Degrees of Freedom: 19 Total (i.e. Null);  18 Residual
Null Deviance:      123.2 
Residual Deviance: 33.78    AIC: 73.24
```


```
#make pretty selbal  cv_bact_resp
png('./files/SHT_selbal/2020-09-20_selbal_cv_metagen_PD_AC.png', width=2500, height=2500, res=400)
```


```
grid.draw(cv_metagen_AC_PD$global.plot)
dev.off()
```


```
png 
  2
```


```
#metagen selbal C PD
metagen_map_noPa_AC
```


```
table(metagen_noPa>10)
```


```
TRUE 
1240
```


```
metagen_map_noPa_C <- metagen_map_noPa_AC[grep('C',metagen_map_noPa_AC$DiseasClass),]
metagen_df_noPa_C <-metagen_noPa[,rownames(metagen_map_noPa_C)]
rownames(metagen_df_noPa_C) <-gsub('.*g__','',rownames(metagen_df_noPa_C))
metagen_df_noPa_C
```


```
var_metagen <- as.numeric(metagen_map_noPa_C$PocketDepth)

#selbal needs two inputs- matrix with samples as rows and taxa as column
#setup 
metagen_genus_sum_t <- t(metagen_df_noPa_C)#
table(metagen_genus_sum_t ==0)
```


```
FALSE 
  620
```


```
#selbal pre resp
cv_metagen_C_PD <- selbal.cv(x=metagen_genus_sum_t, y=var_metagen, zero.rep="bayes",seed = 1,col = c("#0072B2","#D55E00"))
```


```
############################################################### 
 STARTING selbal.cv FUNCTION 
###############################################################

#-------------------------------------------------------------# 
# ZERO REPLACEMENT . . .


, . . . FINISHED. 
#-------------------------------------------------------------#

#-------------------------------------------------------------# 
# Starting the cross - validation procedure . . .
```


```
already exporting variable(s): logit.acc
```


```
 . . . finished. 
#-------------------------------------------------------------# 
###############################################################

 The optimal number of variables is: 2 


############################################################### 
 . . . FINISHED. 
###############################################################
```


```
cv_metagen_C_PD$accuracy.nvar
```


```
cv_metagen_C_PD$var.barplot
cv_metagen_C_PD$glm
```


```
Call:  glm(formula = numy ~ ., family = f.class, data = U)

Coefficients:
(Intercept)           V1  
      1.585        2.275  

Degrees of Freedom: 9 Total (i.e. Null);  8 Residual
Null Deviance:      70.4 
Residual Deviance: 10.71    AIC: 35.07
```


```
#make pretty selbal  cv_bact_resp
png('./files/SHT_selbal/2020-09-20_selbal_cv_metagen_PD_C.png', width=2500, height=2500, res=400)
```


```
grid.draw(cv_metagen_C_PD$global.plot)
dev.off()
```


```
png 
  2
```


LS0tDQp0aXRsZTogIlIgc2VsYmFsIE5vdGVib29rIg0Kb3V0cHV0OiBodG1sX25vdGVib29rDQotLS0NCg0KYGBge3J9DQojaW1wb3J0IHBhY2thZ2VzDQpsaWJyYXJ5KHByb3ByKQ0KbGlicmFyeShtYWdyaXR0cikNCmxpYnJhcnkoekNvbXBvc2l0aW9ucykNCmxpYnJhcnkobWFncml0dHIpDQpsaWJyYXJ5KGRwbHlyKQ0KbGlicmFyeShnZ3Bsb3QyKQ0KbGlicmFyeSh0aWR5dmVyc2UpIA0KbGlicmFyeShyZWFkcikNCmxpYnJhcnkobWl4T21pY3MpDQpsaWJyYXJ5KHNlbGJhbCkNCmxpYnJhcnkodmVnYW4pDQpsaWJyYXJ5KHNjYWxlcykNCmxpYnJhcnkoZ2d0aGVtZXMpDQpyZXF1aXJlKGd0b29scykNCmxpYnJhcnkodmlyaWRpcykNCmxpYnJhcnkoZ2dwdWJyKQ0KbGlicmFyeShjb3JycGxvdCkNCmxpYnJhcnkocHN5Y2gpDQpsaWJyYXJ5KG1hZ2ljaykNCmxpYnJhcnkocGRmdG9vbHMpDQpsaWJyYXJ5KHBuZykNCmxpYnJhcnkoZ3JpZCkNCmxpYnJhcnkoZ3JpZEV4dHJhKQ0KYGBgDQoNClRoaXMgaXMgYW4gW1IgTWFya2Rvd25dKGh0dHA6Ly9ybWFya2Rvd24ucnN0dWRpby5jb20pIE5vdGVib29rLiBXaGVuIHlvdSBleGVjdXRlIGNvZGUgd2l0aGluIHRoZSBub3RlYm9vaywgdGhlIHJlc3VsdHMgYXBwZWFyIGJlbmVhdGggdGhlIGNvZGUuIA0KDQpUcnkgZXhlY3V0aW5nIHRoaXMgY2h1bmsgYnkgY2xpY2tpbmcgdGhlICpSdW4qIGJ1dHRvbiB3aXRoaW4gdGhlIGNodW5rIG9yIGJ5IHBsYWNpbmcgeW91ciBjdXJzb3IgaW5zaWRlIGl0IGFuZCBwcmVzc2luZyAqQ3RybCtTaGlmdCtFbnRlciouIA0KDQpgYGB7cn0gDQojUFQgZGF0YSByZWFkIGluDQojdGFibGVzDQpQVF9iYWN0X2dlbnVzX3N1bV91bnR4X3NlbGJhbCA8LSByZWFkLnRhYmxlKCcuL2ZpbGVzLzIwMjAtMDktMThfUFRfMTZTX09UVV9nZW51c19UYWJsLmNzdicsc2VwPScsJyxyb3cubmFtZXMgPSAxLGhlYWRlciA9IDEsY2hlY2submFtZXM9RkFMU0UpDQpQVF9jeXRvX3VudHhfdCA8LSByZWFkLnRhYmxlKCcuL2ZpbGVzLzIwMjAtMDktMThfUFRfY3l0b190YWJsZS5jc3YnLHNlcD0nLCcsaGVhZGVyID0xLHJvdy5uYW1lcyA9IDEsY2hlY2submFtZXMgPSBGLG5hLnN0cmluZ3MgPSAnTkQnKQ0KUFRfY3l0b191bnR4IDwtIHQoUFRfY3l0b191bnR4X3QpDQpQVF9tZXRhZ2VuX2dlbnVzXzEwX3JlZHVjZV9zbGltIDwtIHJlYWQudGFibGUoJy4vZmlsZXMvMjAyMC0wOS0xOF9QVF9tZXRhZ2VuX2dlbnVzX3RhYmxlLmNzdicsc2VwPScsJyxyb3cubmFtZXMgPSAxLGhlYWRlciA9IDEsY2hlY2submFtZXM9RkFMU0UpDQoNCmBgYA0KDQoNCmBgYHtyfQ0KI1BUIHJlYWQgaW4gYW5kIGZpbHRlciBtYXBzDQojaW1wb3J0IHNhbXBsZSBtYXBzDQpQVF9iYWN0X21hcCA8LSByZWFkLnRhYmxlKCcuL2ZpbGVzL1NjaHdhcnpiZXJnX2V0X2FsX21hcHBpbmdmaWxlLnR4dCcsc2VwPSdcdCcsaGVhZGVyID0xLHJvdy5uYW1lcyA9IDEpDQpQVF9iYWN0X21hcF9vcmRlcmVkIDwtIFBUX2JhY3RfbWFwW29yZGVyKFBUX2JhY3RfbWFwJFNhbXBsZSksXQ0KUFRfYmFjdF9tYXBfbGFyZ2UgPC0gcmVhZC5jc3YoJy4vZmlsZXMvYWxsNzZwdF9tYXAudHh0LmNzdicsIHJvdy5uYW1lcz0xLGhlYWRlcj0xKQ0KUFRfY3l0b19tYXAgPC0gYXMuZGF0YS5mcmFtZShyZWFkLmNzdignLi9maWxlcy9QRFZhbHVlc19MREZfQ3l0b2tpbmVSZXN1bHRzLmNzdicpKQ0KI2RpbShjeXRvX2RmKQ0KI2R1cGxpY2F0ZSBtYXAgdG8gcmVsZWN0IGN5dG8ga2luZSBzYW1wbGVzIGluIHRhYmxlDQpQVF9jeXRvX21hcF9kdXBsIDwtIFBUX2N5dG9fbWFwW3JlcChyb3cubmFtZXMoUFRfY3l0b19tYXApLCBQVF9jeXRvX21hcCRDYXNlX3N0YXR1cysxKSxdICU+JQ0KICBhcnJhbmdlKFBJRCkgDQpQVF9jeXRvX21hcF9kdXBsJENhc2Vfc3RhdHVzW2R1cGxpY2F0ZWQoUFRfY3l0b19tYXBfZHVwbFsxOjJdKV0gPC0gUFRfY3l0b19tYXBfZHVwbCRDYXNlX3N0YXR1c1tkdXBsaWNhdGVkKFBUX2N5dG9fbWFwX2R1cGxbMToyXSldICsxDQpQVF9tZXRhZ2VuX21hcDwtIHJlYWQudGFibGUoJy4vZmlsZXMvbWFwcGluZ19maWxlX1BULnR4dCcsc2VwPSdcdCcsaGVhZGVyID0xLHJvdy5uYW1lcyA9IDEpDQpQVF9tZXRhZ2VuX21hcF9vcmRlcmVkIDwtIFBUX21ldGFnZW5fbWFwW29yZGVyKHJvdy5uYW1lcyhQVF9tZXRhZ2VuX21hcCkpLG9yZGVyKGNvbG5hbWVzKFBUX21ldGFnZW5fbWFwKSldDQpQVF9tZXRhZ2VuX21hcF9vcmRlcmVkJFBlcmlvVHJlYXRtZW50IDwtIGdzdWIoJzF8MicsJycsUFRfbWV0YWdlbl9tYXBfb3JkZXJlZCRQZXJpb1RyZWF0bWVudCkNCg0KI2ZpbHRlciBtZXRhZ2VuDQpQVF9tZXRhZ2VuX2RmX2ZpbHRlcmVkIDwtIFBUX21ldGFnZW5fZ2VudXNfMTBfcmVkdWNlX3NsaW1bLGNvbG5hbWVzKFBUX21ldGFnZW5fZ2VudXNfMTBfcmVkdWNlX3NsaW0pICVpbiVyb3cubmFtZXMoUFRfbWV0YWdlbl9tYXBfb3JkZXJlZCldIA0KDQojR2V0IGludGVyZXN0aW5nIFBEIGZyb20gY3l0b2tpbmUgbWFwDQpQVF9iYWN0X21hcF9QRCA8LSBQVF9jeXRvX21hcF9kdXBsWzE6OTQsXSANCg0KIyMjd2hpY2ggc2FtcGxlcyB0byBrZWVwOyBvbmx5IGFuYWx5emluZyBpbXByb3ZlZCBhbmQgd29yc2VuZWQ7IA0KUFRfY3l0b19tYXBfaW1wcm92ZWRfd29yc2VuZWQgPC0gUFRfY3l0b19tYXBfZHVwbFtncmVwKCdbMS05XScsUFRfY3l0b19tYXBfZHVwbCRQRFN1bWRpZmYpLF0NClBUX2N5dG9fbWFwX2ltcHJvdmVkX3dvcnNlbmVkJFBvY2tldFJlc3BvbnNlIDwtICdJbXByb3ZlZCcNClBUX2N5dG9fbWFwX2ltcHJvdmVkX3dvcnNlbmVkJFBvY2tldFJlc3BvbnNlW1BUX2N5dG9fbWFwX2ltcHJvdmVkX3dvcnNlbmVkJFBEU3VtZGlmZiA8IDBdIDwtICdXb3JzZW5lZCcNClBUX2N5dG9fbWFwX2ltcHJvdmVkX3dvcnNlbmVkJFBEU3VtIDwtIGFzLnZlY3RvcihyYmluZChQVF9jeXRvX21hcF9pbXByb3ZlZF93b3JzZW5lZCRQcmVQRHN1bVtzZXEoMSxucm93KFBUX2N5dG9fbWFwX2ltcHJvdmVkX3dvcnNlbmVkKSwyKV0sUFRfY3l0b19tYXBfaW1wcm92ZWRfd29yc2VuZWQkUG9zdFBEc3VtW3NlcSgxLG5yb3coUFRfY3l0b19tYXBfaW1wcm92ZWRfd29yc2VuZWQpLDIpXSkpDQoNCg0KUFRfYmFjdF9tYXBfaW1wcm92ZWRfd29yc2VuZWQgPC0gUFRfYmFjdF9tYXBfbGFyZ2VbZ3JlcCggJ0ltcHJvdmVkfFdvcnNlbmVkJyxQVF9iYWN0X21hcF9sYXJnZSRPdmVyYWxsUmVzcG9uc2UpLF0NClBUX2JhY3RfbWFwX2ltcHJvdmVkX3dvcnNlbmVkJG1ldGFnZW5EZXNjcmlwIDwtIHBhc3RlKHJvd25hbWVzKFBUX2JhY3RfbWFwX2ltcHJvdmVkX3dvcnNlbmVkKSwnLmtyYWtlbicsc2VwID0gJycpDQojYmFjdF9tYXBfUEQkUElEICVpbiUgYXMubnVtZXJpYyhncmVwKCAnSW1wcm92ZWR8V29yc2VuZWQnLGJhY3RfbWFwX2xhcmdlJE92ZXJhbGxSZXNwb25zZSkpDQoNClBUX2JhY3RfbWFwX1BEX3RoZXJlIDwtIFBUX2JhY3RfbWFwX1BEW2JhY3RfbWFwX1BEJFBJRCVpbiVhcy5udW1lcmljKGdzdWIoICdBfEJ8IjAnLCcnLHJvdy5uYW1lcyhQVF9iYWN0X21hcF9pbXByb3ZlZF93b3JzZW5lZCkpKSxdDQoNCg0KYGBgDQoNCg0KDQoNCmBgYHtyfQ0KIyMjU2VsYmFsDQojcmVhZCBpbiBkYXRhDQojaW1wb3J0IGFsbCBjbHIgT1RVIHRhYmxlcyBhbmQgbWV0YWJvbGl0ZSB0YWJsZQ0KYmFjdF9kZiA8LSBQVF9iYWN0X2dlbnVzX3N1bV91bnR4X3NlbGJhbCANCmJhY3RfZGZfb3JkZXJlZCA8LSBiYWN0X2RmW29yZGVyKHJvdy5uYW1lcyhiYWN0X2RmKSksb3JkZXIoY29sbmFtZXMoYmFjdF9kZikpXQ0KY3l0b19kZiA8LSB0KFBUX2N5dG9fdW50eF90KQ0KbWV0YWdlbl9kZiA8LSBQVF9tZXRhZ2VuX2dlbnVzXzEwX3JlZHVjZV9zbGltIA0KbWV0YWdlbl9kZl9vcmRlcmVkIDwtIG1ldGFnZW5fZGZbb3JkZXIocm93Lm5hbWVzKG1ldGFnZW5fZGYpKSwgb3JkZXIoY29sbmFtZXMobWV0YWdlbl9kZikpXQ0KDQojZmlsdGVyIG1ldGFnZW4NCm1ldGFnZW5fZGZfZmlsdGVyZWQgPC0gbWV0YWdlbl9kZl9vcmRlcmVkWyxjb2xuYW1lcyhtZXRhZ2VuX2RmX29yZGVyZWQpJWluJXJvdy5uYW1lcyhtZXRhZ2VuX21hcF9vcmRlcmVkKV0gDQoNCmJhY3RfZ2VudXNfc3VtIDwtIGJhY3RfZGZfb3JkZXJlZFssb3JkZXIoY29sbmFtZXMoYmFjdF9kZl9vcmRlcmVkKSldIyMjYWRkIGhlYWx0aHkgb3Igbm90IHRvIGNvbG5hbWVzDQpgYGANCg0KDQpgYGB7cn0NCg0KDQoNCiNtYWtlIFByZSBQb3N0IGRpY290b21vdXMgdmFyaWFibGUgDQpiYWN0X21hcF9vcmRlcmVkIDwtIFBUX2JhY3RfbWFwX2ltcHJvdmVkX3dvcnNlbmVkI1tvcmRlcihiYWN0X21hcCRTYW1wbGUpLF0gDQpjeXRvX21hcF90b19yZW1vdmUgPC0gUFRfY3l0b19tYXBbKFBUX2N5dG9fbWFwJENhc2Vfc3RhdHVzID09MCksXQ0KYmFjdF9tYXBfb3JkZXJlZF9QcmVQb3N0IDwtIFBUX2JhY3RfbWFwX29yZGVyZWRbIShiYWN0X21hcF9vcmRlcmVkJFBlcmlvVHJlYXRtZW50PT0iSGVhbHRoeSIpLF0NCmJhY3RfbWFwX29yZGVyZWRfUHJlUG9zdCRUcmVhdG1lbnQgPC0gaWZlbHNlKGJhY3RfbWFwX29yZGVyZWRfUHJlUG9zdCRQZXJpb1RyZWF0bWVudD09J1ByZScsJ1ByZScsJ1Bvc3QnKSANCmN5dG9fbWFwX1ByZVBvc3QgPC0gUFRfY3l0b19tYXBfZHVwbFshY3l0b19tYXBfZHVwbCRDYXNlX3N0YXR1cz09MCxdDQpjeXRvX21hcF9QcmUgPC0gUFRfY3l0b19tYXBbY3l0b19tYXAkQ2FzZV9zdGF0dXM9PTEsXQ0KY3l0b19tYXBfUG9zdCA8LSBQVF9jeXRvX21hcFtjeXRvX21hcCRDYXNlX3N0YXR1cz09MSxdDQpiYWN0X21hcF9QcmUgPC0gYmFjdF9tYXBfb3JkZXJlZF9QcmVQb3N0W2JhY3RfbWFwX29yZGVyZWRfUHJlUG9zdCRQZXJpb1RyZWF0bWVudD09J1ByZScsXQ0KYmFjdF9tYXBfUG9zdCA8LWJhY3RfbWFwX29yZGVyZWRfUHJlUG9zdFtiYWN0X21hcF9vcmRlcmVkX1ByZVBvc3QkUGVyaW9UcmVhdG1lbnQ9PSdQb3N0JyxdDQpgYGANCg0KDQpgYGB7cn0NCiNnZXQgb25seSBQcmUgUG9zdCANCmJhY3RfZ2VudXNfc3VtX1ByZVBvc3QgPC0gYmFjdF9nZW51c19zdW1bICwgd2hpY2goY29sbmFtZXMoYmFjdF9nZW51c19zdW0pICVpbiUgcm93Lm5hbWVzKGJhY3RfbWFwX2ltcHJvdmVkX3dvcnNlbmVkKSldDQojYmFjdF9nZW51c19zdW1fUHJlUG9zdA0KI2JhY3RfbWFwX29yZGVyZWRfUHJlUG9zdA0KYmFjdF9nZW51c19zdW1fUHJlIDwtIGJhY3RfZ2VudXNfc3VtWywgd2hpY2goY29sbmFtZXMoYmFjdF9nZW51c19zdW0pICVpbiUgcm93Lm5hbWVzKGJhY3RfbWFwX1ByZSkpXQ0KYmFjdF9nZW51c19zdW1fUHJlPC0gYmFjdF9nZW51c19zdW1fUHJlWywgb3JkZXIoY29sbmFtZXMoYmFjdF9nZW51c19zdW1fUHJlKSldDQoNCmJhY3RfZ2VudXNfc3VtX1Bvc3QgPC0gYmFjdF9nZW51c19zdW1bLCB3aGljaChjb2xuYW1lcyhiYWN0X2dlbnVzX3N1bSkgJWluJSByb3cubmFtZXMoYmFjdF9tYXBfUG9zdCkpXQ0KYmFjdF9nZW51c19zdW1fUG9zdCA8LSBiYWN0X2dlbnVzX3N1bV9Qb3N0Wywgb3JkZXIoY29sbmFtZXMoYmFjdF9nZW51c19zdW1fUG9zdCkpXQ0KYGBgDQoNCg0KYGBge3J9DQoNCmJhY3RfZ2VudXNfdCA8LSB0KGJhY3RfZ2VudXNfc3VtX1ByZVBvc3QpDQpjeXRvX3QgPC0gdChjeXRvX3VudHgpDQptZXRhZ2VuX2dlbnVzX3QgPC0gdChtZXRhZ2VuX2RmX2ZpbHRlcmVkKQ0KDQpiYWN0X3ByZSA8LSBiYWN0X2dlbnVzX3RbZ3JlcCgnQScsIHJvdy5uYW1lcyhiYWN0X2dlbnVzX3QpKSxdDQpjeXRvX3ByZSA8LSBjeXRvX3RbZ3JlcCgnQScsIHJvdy5uYW1lcyhjeXRvX3QpKSxdDQptZXRhZ2VuX3ByZSA8LSBtZXRhZ2VuX2dlbnVzX3RbZ3JlcCgnQScsIHJvdy5uYW1lcyhtZXRhZ2VuX2dlbnVzX3QpKSxdDQojIGJhY3RfY3l0b19wcmUgPC0gcm1ybl9iYWN0X2N5dG9bZ3JlcCgnQScsIHJvdy5uYW1lcyhybXJuX2JhY3RfY3l0bykpLF0NCiMgYmFjdF9tZXRhZ2VuX3ByZSA8LSBybXJuX2JhY3RfbWV0YWdlbltncmVwKCdBJywgcm93Lm5hbWVzKHJtcm5fYmFjdF9jeXRvKSksXQ0KYmFjdF9wb3N0IDwtIGJhY3RfZ2VudXNfdFtncmVwKCdCJywgcm93Lm5hbWVzKGJhY3RfZ2VudXNfdCkpLF0NCmN5dG9fcG9zdCA8LSBjeXRvX3RbZ3JlcCgnQicsIHJvdy5uYW1lcyhjeXRvX3QpKSxdDQptZXRhZ2VuX3Bvc3QgPC0gbWV0YWdlbl9nZW51c190W2dyZXAoJ0InLCByb3cubmFtZXMobWV0YWdlbl9nZW51c190KSksXQ0KIyBiYWN0X2N5dG9fcG9zdCA8LSBybXJuX2JhY3RfY3l0b1tncmVwKCdCJywgcm93Lm5hbWVzKHJtcm5fYmFjdF9jeXRvKSksXQ0KIyBiYWN0X21ldGFnZW5fcG9zdCA8LSBybXJuX2JhY3RfbWV0YWdlbltncmVwKCdCJywgcm93Lm5hbWVzKHJtcm5fYmFjdF9jeXRvKSksXQ0KYGBgDQoNCg0KDQpgYGB7cn0NCmJhY3RfaW1wcm92ZWQgPC0gYmFjdF9nZW51c190W3Jvd25hbWVzKGJhY3RfZ2VudXNfdCklaW4lcm93bmFtZXMoYmFjdF9tYXBfaW1wcm92ZWRfd29yc2VuZWRbYmFjdF9tYXBfaW1wcm92ZWRfd29yc2VuZWQkT3ZlcmFsbFJlc3BvbnNlID09J0ltcHJvdmVkJyxdKSxdDQpiYWN0X3dvcnNlbmVkIDwtIGJhY3RfZ2VudXNfdFtyb3duYW1lcyhiYWN0X2dlbnVzX3QpJWluJXJvd25hbWVzKGJhY3RfbWFwX2ltcHJvdmVkX3dvcnNlbmVkW2JhY3RfbWFwX2ltcHJvdmVkX3dvcnNlbmVkJE92ZXJhbGxSZXNwb25zZSA9PSdXb3JzZW5lZCcsXSksXQ0KYmFjdF9wcmVfaW1wcm92ZWQgPC0gYmFjdF9wcmVbcm93bmFtZXMoYmFjdF9tYXBfaW1wcm92ZWRfd29yc2VuZWRbYmFjdF9tYXBfaW1wcm92ZWRfd29yc2VuZWQkT3ZlcmFsbFJlc3BvbnNlID09J0ltcHJvdmVkJyAmYmFjdF9tYXBfaW1wcm92ZWRfd29yc2VuZWQkUGVyaW9UcmVhdG1lbnQ9PSdQcmUnLF0pLF0NCiMgW3Jvd25hbWVzKGJhY3RfbWFwX2ltcHJvdmVkX3dvcnNlbmVkKVtncmVwKCdBJyxyb3duYW1lcyhiYWN0X21hcF9pbXByb3ZlZF93b3JzZW5lZFtncmVwKCdJbXByb3ZlZCcsYmFjdF9tYXBfaW1wcm92ZWRfd29yc2VuZWQkT3ZlcmFsbFJlc3BvbnNlKSxdKSldLF0NCmJhY3RfcHJlX3dvcnNlbmVkIDwtIGJhY3RfcHJlW3Jvd25hbWVzKGJhY3RfbWFwX2ltcHJvdmVkX3dvcnNlbmVkW2JhY3RfbWFwX2ltcHJvdmVkX3dvcnNlbmVkJE92ZXJhbGxSZXNwb25zZSA9PSdXb3JzZW5lZCcgJmJhY3RfbWFwX2ltcHJvdmVkX3dvcnNlbmVkJFBlcmlvVHJlYXRtZW50PT0nUHJlJyxdKSxdDQpjeXRvX2ltcHJvdmVkIDwtIGN5dG9fdFtyb3duYW1lcyhiYWN0X21hcF9pbXByb3ZlZF93b3JzZW5lZFtiYWN0X21hcF9pbXByb3ZlZF93b3JzZW5lZCRPdmVyYWxsUmVzcG9uc2UgPT0nSW1wcm92ZWQnLF0pLF0NCmN5dG9fd29yc2VuZWQgPC0gY3l0b190W3Jvd25hbWVzKGJhY3RfbWFwX2ltcHJvdmVkX3dvcnNlbmVkW2JhY3RfbWFwX2ltcHJvdmVkX3dvcnNlbmVkJE92ZXJhbGxSZXNwb25zZSA9PSdXb3JzZW5lZCcsXSksXQ0KY3l0b19wcmVfaW1wcm92ZWQgPC0gY3l0b190W3Jvd25hbWVzKGJhY3RfbWFwX2ltcHJvdmVkX3dvcnNlbmVkW2JhY3RfbWFwX2ltcHJvdmVkX3dvcnNlbmVkJE92ZXJhbGxSZXNwb25zZSA9PSdJbXByb3ZlZCcmYmFjdF9tYXBfaW1wcm92ZWRfd29yc2VuZWQkUGVyaW9UcmVhdG1lbnQ9PSdQcmUnLF0pLF0NCmN5dG9fcHJlX3dvcnNlbmVkIDwtIGN5dG9fdFtyb3duYW1lcyhiYWN0X21hcF9pbXByb3ZlZF93b3JzZW5lZFtiYWN0X21hcF9pbXByb3ZlZF93b3JzZW5lZCRPdmVyYWxsUmVzcG9uc2UgPT0nV29yc2VuZWQnICZiYWN0X21hcF9pbXByb3ZlZF93b3JzZW5lZCRQZXJpb1RyZWF0bWVudD09J1ByZScsXSksXQ0KbWV0YWdlbl9pbXByb3ZlZCA8LW1ldGFnZW5fZ2VudXNfdFtyb3duYW1lcyhtZXRhZ2VuX2dlbnVzX3QpICVpbiUgcm93bmFtZXMobWV0YWdlbl9tYXBfb3JkZXJlZFttZXRhZ2VuX21hcF9vcmRlcmVkJE92ZXJhbGxSZXNwb25zZSA9PSdJbXByb3ZlZCcgLF0pLF0NCm1ldGFnZW5fd29yc2VuZWQgPC0gbWV0YWdlbl9nZW51c190W3Jvd25hbWVzKG1ldGFnZW5fZ2VudXNfdCkgJWluJSByb3duYW1lcyhtZXRhZ2VuX21hcF9vcmRlcmVkW21ldGFnZW5fbWFwX29yZGVyZWQkT3ZlcmFsbFJlc3BvbnNlID09J1dvcnNlbmVkJyAsXSksXQ0KbWV0YWdlbl9wcmVfaW1wcm92ZWQgPC1tZXRhZ2VuX2dlbnVzX3Rbcm93bmFtZXMobWV0YWdlbl9nZW51c190KSAlaW4lIHJvd25hbWVzKG1ldGFnZW5fbWFwX29yZGVyZWRbbWV0YWdlbl9tYXBfb3JkZXJlZCRPdmVyYWxsUmVzcG9uc2UgPT0nSW1wcm92ZWQnICYgbWV0YWdlbl9tYXBfb3JkZXJlZCRQZXJpb1RyZWF0bWVudD09J1ByZScsXSksXQ0KbWV0YWdlbl9wcmVfd29yc2VuZWQgPC0gbWV0YWdlbl9nZW51c190W3Jvd25hbWVzKG1ldGFnZW5fZ2VudXNfdCkgJWluJSByb3duYW1lcyhtZXRhZ2VuX21hcF9vcmRlcmVkW21ldGFnZW5fbWFwX29yZGVyZWQkT3ZlcmFsbFJlc3BvbnNlID09J1dvcnNlbmVkJyAmIG1ldGFnZW5fbWFwX29yZGVyZWQkUGVyaW9UcmVhdG1lbnQ9PSdQcmUnLF0pLF0NCmJhY3RfcG9zdF9pbXByb3ZlZCA8LSBiYWN0X3Bvc3Rbcm93bmFtZXMoYmFjdF9tYXBfaW1wcm92ZWRfd29yc2VuZWRbYmFjdF9tYXBfaW1wcm92ZWRfd29yc2VuZWQkT3ZlcmFsbFJlc3BvbnNlID09J0ltcHJvdmVkJyAmYmFjdF9tYXBfaW1wcm92ZWRfd29yc2VuZWQkUGVyaW9UcmVhdG1lbnQ9PSdQb3N0JyxdKSxdDQpiYWN0X3Bvc3Rfd29yc2VuZWQgPC0gYmFjdF9wb3N0W3Jvd25hbWVzKGJhY3RfbWFwX2ltcHJvdmVkX3dvcnNlbmVkW2JhY3RfbWFwX2ltcHJvdmVkX3dvcnNlbmVkJE92ZXJhbGxSZXNwb25zZSA9PSdXb3JzZW5lZCcgJmJhY3RfbWFwX2ltcHJvdmVkX3dvcnNlbmVkJFBlcmlvVHJlYXRtZW50PT0nUG9zdCcsXSksXQ0KY3l0b19wb3N0X2ltcHJvdmVkIDwtIGN5dG9fdFtyb3duYW1lcyhiYWN0X21hcF9pbXByb3ZlZF93b3JzZW5lZFtiYWN0X21hcF9pbXByb3ZlZF93b3JzZW5lZCRPdmVyYWxsUmVzcG9uc2UgPT0nSW1wcm92ZWQnICZiYWN0X21hcF9pbXByb3ZlZF93b3JzZW5lZCRQZXJpb1RyZWF0bWVudD09J1Bvc3QnLF0pLF0NCmN5dG9fcG9zdF93b3JzZW5lZCA8LSBjeXRvX3Rbcm93bmFtZXMoYmFjdF9tYXBfaW1wcm92ZWRfd29yc2VuZWRbYmFjdF9tYXBfaW1wcm92ZWRfd29yc2VuZWQkT3ZlcmFsbFJlc3BvbnNlID09J1dvcnNlbmVkJyAmYmFjdF9tYXBfaW1wcm92ZWRfd29yc2VuZWQkUGVyaW9UcmVhdG1lbnQ9PSdQb3N0JyxdKSxdDQptZXRhZ2VuX3Bvc3RfaW1wcm92ZWQgPC0gbWV0YWdlbl9nZW51c190W3Jvd25hbWVzKG1ldGFnZW5fZ2VudXNfdCkgJWluJSByb3duYW1lcyhtZXRhZ2VuX21hcF9vcmRlcmVkW21ldGFnZW5fbWFwX29yZGVyZWQkT3ZlcmFsbFJlc3BvbnNlID09J0ltcHJvdmVkJyAmbWV0YWdlbl9tYXBfb3JkZXJlZCRQZXJpb1RyZWF0bWVudD09J1Bvc3QnLF0pLF0NCm1ldGFnZW5fcG9zdF93b3JzZW5lZCA8LSBtZXRhZ2VuX2dlbnVzX3Rbcm93bmFtZXMobWV0YWdlbl9nZW51c190KSAlaW4lIHJvd25hbWVzKG1ldGFnZW5fbWFwX29yZGVyZWRbbWV0YWdlbl9tYXBfb3JkZXJlZCRPdmVyYWxsUmVzcG9uc2UgPT0nV29yc2VuZWQnICZtZXRhZ2VuX21hcF9vcmRlcmVkJFBlcmlvVHJlYXRtZW50PT0nUG9zdCcsXSksXQ0KYGBgDQoNCg0KDQoNCg0KDQpgYGB7cn0NCiMjIyMjIyNQcmUgdnMgUG9zdA0KIyAjc2VsYmFsIG5lZWRzIHZlY3RvciB3aXRoIHJlc3BvbnNlIHZhcmlhYmxlLiBNYWtlIHZlY3RvcnM6IChhcyBmYWN0b3IpDQojICNjYW4gb25seSBkbyBkaWNob3RvbW91cyB2YXJpYWJsZXMtIFByZS1Qb3N0IGFuZCBjb250aW51b3VzLSBwb2NrZXRkZXB0aA0KIyANCiMgI3NlbGJhbCBuZWVkcyB0d28gaW5wdXRzLSBtYXRyaXggd2l0aCBzYW1wbGVzIGFzIHJvd3MgYW5kIHRheGEgYXMgY29sdW1uDQojc2V0dXAgDQpiYWN0X2dlbnVzX3N1bSA8LSBiYWN0X2dlbnVzX3N1bV9QcmVQb3N0IA0KYmFjdF9nZW51c19zdW1fdCA8LSB0KGJhY3RfZ2VudXNfc3VtWyxvcmRlcihjb2xuYW1lcyhiYWN0X2dlbnVzX3N1bSkpXSkjIyNhZGQgaGVhbHRoeSBvciBub3QgdG8gY29sbmFtZXMNCmJhY3RfZ2VudXNfc3VtX3QgPC0gYXMuZGF0YS5mcmFtZShiYWN0X2dlbnVzX3N1bV90KSNbIC1ncmVwKCJUUDAiLCByb3cubmFtZXMoYmFjdF9nZW51c19zdW1fdCkpLF0pDQpiYWN0X21hcF9pbXByb3ZlZF93b3JzZW5lZA0KYmFjdF9tYXAgPC0gYmFjdF9tYXBfaW1wcm92ZWRfd29yc2VuZWQjWyAtZ3JlcCgiVFAwIiwgcm93Lm5hbWVzKGJhY3RfbWFwKSksXQ0KYmFjdF90cmVhdG1lbnRfdmFyIDwtIGFzLmZhY3RvcihiYWN0X21hcCRQZXJpb1RyZWF0bWVudCkNCmJhY3RfdHJlYXRtZW50X3ZhciA8LSBhcy5mYWN0b3IoaWZlbHNlKGJhY3RfdHJlYXRtZW50X3ZhciA9PSAnUG9zdCcsJzJQb3N0JywnMVByZScpKQ0KcmVxdWlyZShndG9vbHMpDQojYmFjdF9nZW51c19zdW1fbm8wPC1iYWN0X2dlbnVzX3N1bV9ubzBbbWl4ZWRzb3J0KCByb3duYW1lcyggYmFjdF9tYXBfbm8wICkgKSxdDQpucm93KGJhY3RfZ2VudXNfc3VtKQ0KbGVuZ3RoKGJhY3RfdHJlYXRtZW50X3ZhcikNCg0KYmFjdF9jdl90cmVhdG1lbnQgPC0gc2VsYmFsLmN2KHg9YmFjdF9nZW51c19zdW1fdCwgeT1iYWN0X3RyZWF0bWVudF92YXIsbi5mb2xkPTMsIHplcm8ucmVwPSJiYXllcyIsc2VlZCA9IDEsY29sID0gYygiIzAwNzJCMiIsIiNENTVFMDAiKSkNCmJhY3RfY3ZfdHJlYXRtZW50JGFjY3VyYWN5Lm52YXINCiNiYXJwbG90IHJlcHJlc2VudGluZyB0aGUgZnJlcXVlbmN5IG9mIHRoZSB2YXJpYWJsZXMgc2VsZWN0ZWQgDQojaW4gc29tZSBzdGVwIG9mIHRoZSBDViBwcm9jZXNzIA0KYmFjdF9jdl90cmVhdG1lbnQkdmFyLmJhcnBsb3QNCmdyaWQuZHJhdyhiYWN0X2N2X3RyZWF0bWVudCRnbG9iYWwucGxvdCkNCnBsb3QudGFiKGJhY3RfY3ZfdHJlYXRtZW50JGN2LnRhYikNCmJhY3RfY3ZfdHJlYXRtZW50JGdsbQ0KYmFjdF9jdl90cmVhdG1lbnQkZ2xvYmFsLmJhbGFuY2UNCg0KI3NlbGJhbCBtYWtlIHByZXR0eQ0KcG5nKCcuL2ZpbGVzL1BUX3NlbGJhbC8yMDIwLTA5LTIxX2NiX3NlbGJhbF9iYWN0X3RyZWF0bWVudC5wbmcnLCB3aWR0aD00ODAwLCBoZWlnaHQ9MjkwMCwgcmVzPTMxNSkNCmdyaWQuZHJhdyhiYWN0X2N2X3RyZWF0bWVudCRnbG9iYWwucGxvdCkNCmRldi5vZmYoKQ0KcG5nKCcuL2ZpbGVzL1BUX3NlbGJhbC8yMDIwLTA5LTIxX2JfY2Jfc2VsYmFsX2JhY3RfdHJlYXRtZW50LnBuZycsIHdpZHRoPTI1MDAsIGhlaWdodD0yNTAwLCByZXM9MzE1KQ0KZ3JpZC5kcmF3KGJhY3RfY3ZfdHJlYXRtZW50JGdsb2JhbC5wbG90KQ0KZGV2Lm9mZigpDQpgYGANCg0KDQoNCg0KDQpgYGB7cn0NCiMgI3NlbGJhbCBuZWVkcyB2ZWN0b3Igd2l0aCByZXNwb25zZSB2YXJpYWJsZS4gTWFrZSB2ZWN0b3JzOiAoYXMgZmFjdG9yKQ0KIyAjY2FuIG9ubHkgZG8gZGljaG90b21vdXMgdmFyaWFibGVzLSB3ZXQtZHJ5IGFuZCBjb250aW51b3VzLSB0aW1lDQojIGRlcHRoX3Zhcl9iYWN0IDwtIGFzLm51bWVyaWMoKChiYWN0X21hcF9pbXByb3ZlZF93b3JzZW5lZCRBdmdQb2NrZXREZXB0aCkpKSMqLjEpDQojIA0KIyAjc2VsYmFsIG5lZWRzIHR3byBpbnB1dHMtIG1hdHJpeCB3aXRoIHNhbXBsZXMgYXMgcm93cyBhbmQgdGF4YSBhcyBjb2x1bW4NCg0KIyMjIyMjIyNub3cgZG8gUEQvUEQgc3VtDQpiYWN0X21hcF9pbXByb3ZlZF93b3JzZW5lZCRQRHN1bQ0KcGRzdW1fdmFyX2JhY3QgPC0gYXMubnVtZXJpYygoKGJhY3RfbWFwX2ltcHJvdmVkX3dvcnNlbmVkJFBEc3VtKSkpIyouMSkNCm5jb2woYmFjdF9nZW51c19zdW1fdCkNCm5yb3coYmFjdF9nZW51c19zdW1fdCkNCmxlbmd0aChwZHN1bV92YXJfYmFjdCkNCmN2X2JhY3RfcGRzdW0gPC0gc2VsYmFsLmN2KHg9YmFjdF9nZW51c19zdW1fdCwgeT1wZHN1bV92YXJfYmFjdCwgemVyby5yZXAgPSAnYmF5ZXMnLHNlZWQgPSAxLGNvbCA9IGMoIiMwMDcyQjIiLCIjRDU1RTAwIikpDQpjdl9iYWN0X3Bkc3VtJGFjY3VyYWN5Lm52YXINCmN2X2JhY3RfcGRzdW0kdmFyLmJhcnBsb3QNCmN2X2JhY3RfcGRzdW0kZ2xtDQpncmlkLmRyYXcoY3ZfYmFjdF9wZHN1bSRnbG9iYWwucGxvdCkNCnBsb3QudGFiKGN2X2JhY3RfcGRzdW0kY3YudGFiKQ0KDQojbWFrZSBwcmV0dHkgc2VsYmFsICBjdl9iYWN0X3Bkc3VtDQpwbmcoJy4vZmlsZXMvUFRfc2VsYmFsLzIwMjAtMDktMjBfY2Jfc2VsYmFsX2N2X2JhY3RfcGRzdW0ucG5nJywgd2lkdGg9MjUwMCwgaGVpZ2h0PTI1MDAsIHJlcz00MDApDQpncmlkLmRyYXcoY3ZfYmFjdF9wZHN1bSRnbG9iYWwucGxvdCkNCmRldi5vZmYoKQ0KDQpgYGANCg0KDQoNCmBgYHtyfQ0KDQojc2VsYmFsIHBvc2tldCBpbXByb3Ygd29yc2VuZWQNCmJhY3RfaW1wcm92X3dvcl92YXIgPC0gYXMuZmFjdG9yKGJhY3RfbWFwX2ltcHJvdmVkX3dvcnNlbmVkJE92ZXJhbGxSZXNwb25zZSkNCmN2X2JhY3RfaW1wcm92X3dvciA8LSBzZWxiYWwuY3YoeD1iYWN0X2dlbnVzX3N1bV90LHk9YmFjdF9pbXByb3Zfd29yX3Zhcix6ZXJvLnJlcCA9ICJiYXllcyIsc2VlZCA9IDEsY29sID0gYygiIzAwNzJCMiIsIiNENTVFMDAiKSkNCmN2X2JhY3RfaW1wcm92X3dvciRhY2N1cmFjeS5udmFyDQpjdl9iYWN0X2ltcHJvdl93b3IkdmFyLmJhcnBsb3QNCnZfYmFjdF9pbXByb3Zfd29yJGdsbQ0KZ3JpZC5kcmF3KGN2X2JhY3RfaW1wcm92X3dvciRnbG9iYWwucGxvdCkNCnBsb3QudGFiKGN2X2JhY3RfaW1wcm92X3dvciRjdi50YWIpDQoNCiNtYWtlIHByZXR0eSBzZWxiYWwgIGN2X2JhY3RfcGRzdW0NCnBuZygnLi9maWxlcy9QVF9zZWxiYWwvMjAyMC0wOS0yMF9jYl9zZWxiYWxfY3ZfYmFjdF9pbXByb3Zfd29yLnBuZycsIHdpZHRoPTUwMDAsIGhlaWdodD0yOTAwLCByZXM9NDAwKQ0KZ3JpZC5kcmF3KGN2X2JhY3RfaW1wcm92X3dvciRnbG9iYWwucGxvdCkNCmRldi5vZmYoKQ0KcG5nKCcuL2ZpbGVzL1BUX3NlbGJhbC8yMDIwLTA5LTIwX2NiX3NlbGJhbF9jdl9iYWN0X2ltcHJvdl93b3IucG5nJywgd2lkdGg9MjUwMCwgaGVpZ2h0PTI1MDAsIHJlcz00MDApDQpncmlkLmRyYXcoY3ZfYmFjdF9pbXByb3Zfd29yJGdsb2JhbC5wbG90KQ0KZGV2Lm9mZigpDQpgYGANCg0KDQoNCmBgYHtyfQ0KIyMgSW1wcm92ZWQgcHJldnNwb3N0DQp2YXIgPC0gYXMuZmFjdG9yKGJhY3RfbWFwX2ltcHJvdmVkX3dvcnNlbmVkWydJbXByb3ZlZCc9PWJhY3RfbWFwX2ltcHJvdmVkX3dvcnNlbmVkJE92ZXJhbGxSZXNwb25zZSxdJFBlcmlvVHJlYXRtZW50KQ0KdmFyIDwtIGFzLmZhY3RvcihpZmVsc2UodmFyID09ICdQb3N0JywnMlBvc3QnLCcxUHJlJykpDQpzZWJhbF90IDwtIChiYWN0X2ltcHJvdmVkKQ0KDQojc2VsYmFsIA0KY3ZfYmFjdF9pbXByb3ZlZCA8LSBzZWxiYWwuY3YoeD1zZWJhbF90LCB5PXZhciwgemVyby5yZXA9Im9uZSIsc2VlZCA9IDEsY29sID0gYygiIzAwNzJCMiIsIiNENTVFMDAiKSkNCmN2X2JhY3RfaW1wcm92ZWQkYWNjdXJhY3kubnZhcg0KY3ZfYmFjdF9pbXByb3ZlZCR2YXIuYmFycGxvdA0KZ3JpZC5kcmF3KGN2X2JhY3RfaW1wcm92ZWQkZ2xvYmFsLnBsb3QpDQpwbG90LnRhYihjdl9iYWN0X2ltcHJvdmVkJGN2LnRhYikNCmN2X2JhY3RfaW1wcm92ZWQkZ2xtDQoNCiNtYWtlIHByZXR0eSBzZWxiYWwgIGN2X2JhY3QNCnBuZygnLi9maWxlcy9QVF9zZWxiYWwvMjAyMC0wOS0yMF9jYl9zZWxiYWxfY3ZfYmFjdF9pbXByb3ZlZF9wcmVwb3N0LnBuZycsIHdpZHRoPTUwMDAsIGhlaWdodD0yOTAwLCByZXM9NDAwKQ0KZ3JpZC5kcmF3KGN2X2JhY3RfaW1wcm92ZWQkZ2xvYmFsLnBsb3QpDQpkZXYub2ZmKCkNCnBuZygnLi9maWxlcy9QVF9zZWxiYWwvMjAyMC0wOS0yMF9jYl9zZWxiYWxfY3ZfYmFjdF9pbXByb3ZlZF9wcmVwb3N0LnBuZycsIHdpZHRoPTI1MDAsIGhlaWdodD0yNTAwLCByZXM9NDAwKQ0KZ3JpZC5kcmF3KGN2X2JhY3RfaW1wcm92ZWQkZ2xvYmFsLnBsb3QpDQpkZXYub2ZmKCkNCmBgYA0KDQoNCg0KYGBge3J9DQojIyBXb3JzZW5lZCBwcmV2c3Bvc3QNCnZhciA8LSBhcy5mYWN0b3IoYmFjdF9tYXBfaW1wcm92ZWRfd29yc2VuZWRbJ1dvcnNlbmVkJz09YmFjdF9tYXBfaW1wcm92ZWRfd29yc2VuZWQkT3ZlcmFsbFJlc3BvbnNlLF0kUGVyaW9UcmVhdG1lbnQpDQp2YXIgPC0gYXMuZmFjdG9yKGlmZWxzZSh2YXIgPT0gJ1Bvc3QnLCcyUG9zdCcsJzFQcmUnKSkNCnNlYmFsX3QgPC0gKGJhY3Rfd29yc2VuZWQpDQoNCiNzZWxiYWwgdGltZSB3ZXQNCmN2X2JhY3Rfd29yc2VuZWQgPC0gc2VsYmFsLmN2KHg9c2ViYWxfdCwgeT12YXIsIHplcm8ucmVwPSJiYXllcyIsc2VlZCA9IDEsY29sID0gYygiIzAwNzJCMiIsIiNENTVFMDAiKSkNCmN2X2JhY3Rfd29yc2VuZWQkYWNjdXJhY3kubnZhcg0KY3ZfYmFjdF93b3JzZW5lZCR2YXIuYmFycGxvdA0KZ3JpZC5kcmF3KGN2X2JhY3Rfd29yc2VuZWQkZ2xvYmFsLnBsb3QpDQpwbG90LnRhYihjdl9iYWN0X3dvcnNlbmVkJGN2LnRhYikNCmN2X2JhY3Rfd29yc2VuZWQkZ2xtDQoNCiNtYWtlIHByZXR0eSBzZWxiYWwgIGN2Xw0KcG5nKCcuL2ZpbGVzL1BUX3NlbGJhbC8yMDIwLTA5LTIwX2NiX3NlbGJhbF9jdl9iYWN0X3dvcnNlbmVkX3ByZXBvc3QucG5nJywgd2lkdGg9NTAwMCwgaGVpZ2h0PTI5MDAsIHJlcz00MDApDQpncmlkLmRyYXcoY3ZfYmFjdF93b3JzZW5lZCRnbG9iYWwucGxvdCkNCmRldi5vZmYoKQ0KcG5nKCcuL2ZpbGVzL1BUX3NlbGJhbC8yMDIwLTA5LTIwX2JfY2Jfc2VsYmFsX2N2X2JhY3Rfd29yc2VuZWRfcHJlcG9zdC5wbmcnLCB3aWR0aD0yNTAwLCBoZWlnaHQ9MjUwMCwgcmVzPTQwMCkNCmdyaWQuZHJhdyhjdl9iYWN0X3dvcnNlbmVkJGdsb2JhbC5wbG90KQ0KZGV2Lm9mZigpDQpgYGANCg0KDQoNCmBgYHtyfQ0KIyMgSW1wcm92ZWQgc3VtIGRpZmYgb3Igc3VtIHBkID8NCmJhY3RfbWFwX2ltcHJvdmVkX3dvcnNlbmVkWydJbXByb3ZlZCc9PWJhY3RfbWFwX2ltcHJvdmVkX3dvcnNlbmVkJE92ZXJhbGxSZXNwb25zZSxdJFBEc3VtDQpzYW1wbGVzIDwtIHJvdy5uYW1lcyhiYWN0X21hcF9pbXByb3ZlZF93b3JzZW5lZFsnSW1wcm92ZWQnPT1iYWN0X21hcCRPdmVyYWxsUmVzcG9uc2UsXSkNCnZhciA8LSBhcy5udW1lcmljKChiYWN0X21hcF9pbXByb3ZlZF93b3JzZW5lZFtzYW1wbGVzLF0kUERzdW0pKQ0Kc2ViYWxfdCA8LSAoYmFjdF9pbXByb3ZlZCkNCiMgc2ViYWxfX3QgPC0gc2ViYWxfdFssY29sU3VtcyhzZWJhbF90ID09IDApIDw9IDAuOSAqbnJvdyhzZWJhbF90KV0NCg0KI3NlbGJhbCB0aW1lIHdldA0KY3ZfYmFjdF9pbXByb3ZlZF9wZHN1bSA8LSBzZWxiYWwuY3YoeD1zZWJhbF90LCB5PXZhciwgemVyby5yZXA9Im9uZSIsc2VlZCA9IDEsY29sID0gYygiIzAwNzJCMiIsIiNENTVFMDAiKSkNCmN2X2JhY3RfaW1wcm92ZWRfcGRzdW0kYWNjdXJhY3kubnZhcg0KY3ZfYmFjdF9pbXByb3ZlZF9wZHN1bSR2YXIuYmFycGxvdA0KZ3JpZC5kcmF3KGN2X2JhY3RfaW1wcm92ZWRfcGRzdW0kZ2xvYmFsLnBsb3QpDQpwbG90LnRhYihjdl9iYWN0X2ltcHJvdmVkX3Bkc3VtJGN2LnRhYikNCmN2X2JhY3RfaW1wcm92ZWRfcGRzdW0kZ2xtDQoNCiNtYWtlIHByZXR0eSBzZWxiYWwgIGN2Xw0KcG5nKCcuL2ZpbGVzL1BUX3NlbGJhbC8yMDIwLTA5LTIwX2NiX3NlbGJhbF9jdl9iYWN0X2ltcHJvdmVkX1BEc3VtLnBuZycsIHdpZHRoPTI1MDAsIGhlaWdodD0yNTAwLCByZXM9NDAwKQ0KZ3JpZC5kcmF3KGN2X2JhY3RfaW1wcm92ZWRfcGRzdW0kZ2xvYmFsLnBsb3QpDQpkZXYub2ZmKCkNCmBgYA0KDQoNCmBgYHtyfQ0KIyMgd29yc2VuZWQgc3VtIGRpZmYgb3Igc3VtIHBkID8NCmJhY3RfbWFwX2ltcHJvdmVkX3dvcnNlbmVkWydXb3JzZW5lZCc9PWJhY3RfbWFwX2ltcHJvdmVkX3dvcnNlbmVkJE92ZXJhbGxSZXNwb25zZSxdJFBEc3VtDQoNCnNhbXBsZXMgPC0gcm93Lm5hbWVzKGJhY3RfbWFwX2ltcHJvdmVkX3dvcnNlbmVkWydXb3JzZW5lZCc9PWJhY3RfbWFwJE92ZXJhbGxSZXNwb25zZSxdKQ0KdmFyIDwtIGFzLm51bWVyaWMoKGJhY3RfbWFwX2ltcHJvdmVkX3dvcnNlbmVkW3NhbXBsZXMsXSRQRHN1bSkpDQpzZWJhbF90IDwtIChiYWN0X3dvcnNlbmVkKQ0KDQojc2VsYmFsIHRpbWUgd2V0DQpjdl9iYWN0X3dvcnNlbmVkX3Bkc3VtIDwtIHNlbGJhbC5jdih4PXNlYmFsX3QsIHk9dmFyLCB6ZXJvLnJlcD0ib25lIixzZWVkID0gMSxjb2wgPSBjKCIjMDA3MkIyIiwiI0Q1NUUwMCIpKQ0KY3ZfYmFjdF93b3JzZW5lZF9wZHN1bSRhY2N1cmFjeS5udmFyDQpjdl9iYWN0X3dvcnNlbmVkX3Bkc3VtJHZhci5iYXJwbG90DQpncmlkLmRyYXcoY3ZfYmFjdF93b3JzZW5lZF9wZHN1bSRnbG9iYWwucGxvdCkNCnBsb3QudGFiKGN2X2JhY3Rfd29yc2VuZWRfcGRzdW0kY3YudGFiKQ0KY3ZfYmFjdF93b3JzZW5lZF9wZHN1bSRnbG0NCg0KI21ha2UgcHJldHR5IHNlbGJhbCAgY3ZfDQpwbmcoJy4vZmlsZXMvUFRfc2VsYmFsLzIwMjAtMDktMjBfY2Jfc2VsYmFsX2N2X2JhY3Rfd29yc2VuZWRfUERzdW0ucG5nJywgd2lkdGg9MjUwMCwgaGVpZ2h0PTI1MDAsIHJlcz00MDApDQpncmlkLmRyYXcoY3ZfYmFjdF93b3JzZW5lZF9wZHN1bSRnbG9iYWwucGxvdCkNCmRldi5vZmYoKQ0KYGBgDQoNCg0KYGBge3J9DQoNCiNtYWtlIFByZSBQb3N0IGRpY290b21vdXMgdmFyaWFibGUgDQpjeXRvX3VudHhfdF9wcmVwb3N0IDwtIGN5dG9fdFthcy5udW1lcmljKGdzdWIoJ0F8QicsJycscm93bmFtZXMoY3l0b190KSkpJWluJWN5dG9fbWFwX2ltcHJvdmVkX3dvcnNlbmVkJFBJRCxdDQoNCiMjcmVndWxhciBQcmVwb3N0IA0KcHJlcG9zdF92YXIgPC0gYXMuZmFjdG9yKGN5dG9fbWFwX2ltcHJvdmVkX3dvcnNlbmVkJENhc2Vfc3RhdHVzKQ0KcHJlcG9zdF92YXIgPC0gYXMuZmFjdG9yKGlmZWxzZShwcmVwb3N0X3ZhciA9PSAxLCAnMVByZScsICcyUG9zdCcpKQ0KDQojc2VsYmFsIHRpbWUgd2V0DQpjdl9jeXRvX3ByZXBvc3QgPC0gc2VsYmFsLmN2KHg9Y3l0b191bnR4X3RfcHJlcG9zdCwgeT1wcmVwb3N0X3ZhciwgemVyby5yZXA9ImJheWVzIixzZWVkID0gMSxjb2wgPSBjKCIjMDA3MkIyIiwiI0Q1NUUwMCIpKQ0KY3ZfY3l0b19wcmVwb3N0JGFjY3VyYWN5Lm52YXINCmN2X2N5dG9fcHJlcG9zdCR2YXIuYmFycGxvdA0KZ3JpZC5kcmF3KGN2X2N5dG9fcHJlcG9zdCRnbG9iYWwucGxvdCkNCnBsb3QudGFiKGN2X2N5dG9fcHJlcG9zdCRjdi50YWIpDQpjdl9jeXRvX3ByZXBvc3QkZ2xtDQoNCiNtYWtlIHByZXR0eSBzZWxiYWwgIGN2Xw0KcG5nKCcuL2ZpbGVzL1BUX3NlbGJhbC8yMDIwLTA5LTIwX2NiX3NlbGJhbF9jdl9jeXRvX3ByZXBvc3QucG5nJywgd2lkdGg9NTAwMCwgaGVpZ2h0PTI5MDAsIHJlcz00MDApDQpncmlkLmRyYXcoY3ZfY3l0b19wcmVwb3N0JGdsb2JhbC5wbG90KQ0KZGV2Lm9mZigpDQpwbmcoJy4vZmlsZXMvUFRfc2VsYmFsLzIwMjAtMDktMjBfYl9jYl9zZWxiYWxfY3ZfY3l0b19wcmVwb3N0LnBuZycsIHdpZHRoPTI1MDAsIGhlaWdodD0yNTAwLCByZXM9NDAwKQ0KZ3JpZC5kcmF3KGN2X2N5dG9fcHJlcG9zdCRnbG9iYWwucGxvdCkNCmRldi5vZmYoKQ0KYGBgDQoNCmBgYHtyfQ0KI21ha2UgUHJlIFBvc3QgZGljb3RvbW91cyB2YXJpYWJsZSANCmN5dG9fbWFwX1ByZVBvc3QkUElEDQoNCmN5dG9fbWFwX2ltcHJvdmVkX3dvcnNlbmVkDQpjeXRvX3VudHhfdFthcy5udW1lcmljKGdzdWIoJ0F8QicsJycscm93bmFtZXMoY3l0b191bnR4X3QpKSklaW4lY3l0b19tYXBfaW1wcm92ZWRfd29yc2VuZWQkUElELF0NCmN5dG9fdF9wcmVwb3N0IDwtIGN5dG9fdFtyb3duYW1lcyhjeXRvX3QpJWluJXJvd25hbWVzKGJhY3RfbWFwX2ltcHJvdmVkX3dvcnNlbmVkKSxdDQoNCiMjcmVndWxhciBQcmVwb3N0IA0KDQojcHJlcG9zdF92YXIgPC0gYXMubnVtZXJpYyhjeXRvX21hcF9pbXByb3ZlZF93b3JzZW5lZCRQRFN1bWRpZmYpDQp2YXIgPC0gYXMubnVtZXJpYyhiYWN0X21hcF9pbXByb3ZlZF93b3JzZW5lZFtyb3duYW1lcyhjeXRvX3RfcHJlcG9zdCksXSRQRHN1bSkNCg0KDQojc2VsYmFsIHRpbWUgd2V0DQpjdl9jeXRvX1BEIDwtIHNlbGJhbC5jdih4PWN5dG9fdF9wcmVwb3N0LCB5PXZhciwgemVyby5yZXA9ImJheWVzIixzZWVkID0gMSxjb2wgPSBjKCIjMDA3MkIyIiwiI0Q1NUUwMCIpKQ0KY3ZfY3l0b19QRCRhY2N1cmFjeS5udmFyDQpjdl9jeXRvX1BEJHZhci5iYXJwbG90DQpncmlkLmRyYXcoY3ZfY3l0b19QRCRnbG9iYWwucGxvdCkNCnBsb3QudGFiKGN2X2N5dG9fUEQkY3YudGFiKQ0KY3ZfY3l0b19QRCRnbG0NCg0KI21ha2UgcHJldHR5IHNlbGJhbCAgY3ZfDQpwbmcoJy4vZmlsZXMvUFRfc2VsYmFsLzIwMjAtMDktMjBfY2Jfc2VsYmFsX2N2X2N5dG9fUEQucG5nJywgd2lkdGg9MjUwMCwgaGVpZ2h0PTI1MDAsIHJlcz00MDApDQpncmlkLmRyYXcoY3ZfY3l0b19QRCRnbG9iYWwucGxvdCkNCmRldi5vZmYoKQ0KYGBgDQoNCmBgYHtyfQ0KIyNyZWd1bGFyIGltcHIvd29ycyANCmN5dG9fdF9wcmVwb3N0IDwtIGN5dG9fdFtyb3duYW1lcyhjeXRvX3QpJWluJXJvd25hbWVzKGJhY3RfbWFwX2ltcHJvdmVkX3dvcnNlbmVkKSxdDQojcHJlcG9zdF92YXIgPC0gYXMubnVtZXJpYyhjeXRvX21hcF9pbXByb3ZlZF93b3JzZW5lZCRQRFN1bWRpZmYpDQp2YXIgPC0gYXMuZmFjdG9yKGJhY3RfbWFwX2ltcHJvdmVkX3dvcnNlbmVkW3Jvd25hbWVzKGN5dG9fdF9wcmVwb3N0KSxdJE92ZXJhbGxSZXNwb25zZSkNCg0KDQojc2VsYmFsIHRpbWUgd2V0DQpjdl9jeXRvX09SIDwtIHNlbGJhbC5jdih4PWN5dG9fdF9wcmVwb3N0LCB5PXZhciwgemVyby5yZXA9ImJheWVzIixzZWVkID0gMSxjb2wgPSBjKCIjMDA3MkIyIiwiI0Q1NUUwMCIpKQ0KY3ZfY3l0b19PUiRhY2N1cmFjeS5udmFyDQpjdl9jeXRvX09SJHZhci5iYXJwbG90DQpncmlkLmRyYXcoY3ZfY3l0b19PUiRnbG9iYWwucGxvdCkNCnBsb3QudGFiKGN2X2N5dG9fT1IkY3YudGFiKQ0KY3ZfY3l0b19PUiRnbG0NCg0KI21ha2UgcHJldHR5IHNlbGJhbCAgY3YgY3l0byBPUg0KcG5nKCcuL2ZpbGVzL1BUX3NlbGJhbC8yMDIwLTA5LTIwX2NiX3NlbGJhbF9jdl9jeXRvX09SLnBuZycsIHdpZHRoPTUwMDAsIGhlaWdodD0yOTAwLCByZXM9NDAwKQ0KZ3JpZC5kcmF3KGN2X2N5dG9fT1IkZ2xvYmFsLnBsb3QpDQpkZXYub2ZmKCkNCnBuZygnLi9maWxlcy9QVF9zZWxiYWwvMjAyMC0wOS0yMF9iX2NiX3NlbGJhbF9jdl9jeXRvX09SLnBuZycsIHdpZHRoPTI1MDAsIGhlaWdodD0yNTAwLCByZXM9NDAwKQ0KZ3JpZC5kcmF3KGN2X2N5dG9fT1IkZ2xvYmFsLnBsb3QpDQpkZXYub2ZmKCkNCmBgYA0KDQpgYGB7cn0NCg0KIyNiZXR0ZXIgcHJlIHBvc3QgDQoNCmN5dG9fbWFwX2ltcHJvdmVkX3dvcnNlbmVkDQpjeXRvX3VudHhfdFthcy5udW1lcmljKGdzdWIoJ0F8QicsJycscm93bmFtZXMoY3l0b191bnR4X3QpKSklaW4lY3l0b19tYXBfaW1wcm92ZWRfd29yc2VuZWQkUElELF0NCmN5dG9fdW50eF90X3ByZXBvc3QgPC0gY3l0b191bnR4X3RbYXMubnVtZXJpYyhnc3ViKCdBfEInLCcnLHJvd25hbWVzKGN5dG9fdW50eF90KSkpJWluJWN5dG9fbWFwX2ltcHJvdmVkX3dvcnNlbmVkJFBJRCxdDQoNCmluZGV4X2ltcHJvdmVkIDwtIGFzLm51bWVyaWMoZ3N1YignQXxCfCIwJywnJyxyb3duYW1lcyhjeXRvX2ltcHJvdmVkKSkpDQpwcmVwb3N0X3ZhciA8LSBhcy5mYWN0b3IoY3l0b19tYXBfaW1wcm92ZWRfd29yc2VuZWRbY3l0b19tYXBfaW1wcm92ZWRfd29yc2VuZWQkUElEICVpbiUgaW5kZXhfaW1wcm92ZWQsXSRDYXNlX3N0YXR1cykNCnByZXBvc3RfdmFyIDwtIGFzLmZhY3RvcihpZmVsc2UocHJlcG9zdF92YXIgPT0gMSwgJzFQcmUnLCAnMlBvc3QnKSkNCg0KbnJvdyhjeXRvX2ltcHJvdmVkKQ0KbmNvbChjeXRvX2ltcHJvdmVkKQ0KbGVuZ3RoKHByZXBvc3RfdmFyKQ0KI3NlbGJhbCBiZXR0ZXIgcHJlIHBvc3QgDQpjdl9jeXRvX2ltcHJvdmVkX3ByZXBvc3QgPC0gc2VsYmFsLmN2KHg9Y3l0b19pbXByb3ZlZCwgeT1wcmVwb3N0X3ZhciwgemVyby5yZXA9ImJheWVzIixzZWVkID0gMSxjb2wgPSBjKCIjMDA3MkIyIiwiI0Q1NUUwMCIpKQ0KY3ZfY3l0b19pbXByb3ZlZF9wcmVwb3N0JGFjY3VyYWN5Lm52YXINCmN2X2N5dG9faW1wcm92ZWRfcHJlcG9zdCR2YXIuYmFycGxvdA0KZ3JpZC5kcmF3KGN2X2N5dG9faW1wcm92ZWRfcHJlcG9zdCRnbG9iYWwucGxvdCkNCnBsb3QudGFiKGN2X2N5dG9faW1wcm92ZWRfcHJlcG9zdCRjdi50YWIpDQpjdl9jeXRvX2ltcHJvdmVkX3ByZXBvc3QkZ2xtDQoNCiNtYWtlIHByZXR0eSBzZWxiYWwgIGJldHRlciBwcmUgcG9zdCANCnBuZygnLi9maWxlcy9QVF9zZWxiYWwvMjAyMC0wOS0yMF9jYl9zZWxiYWxfY3ZfY3l0b19pbXByb3ZlZF9wcmVwb3N0LnBuZycsIHdpZHRoPTUwMDAsIGhlaWdodD0yOTAwLCByZXM9NDAwKQ0KZ3JpZC5kcmF3KGN2X2N5dG9faW1wcm92ZWRfcHJlcG9zdCRnbG9iYWwucGxvdCkNCmRldi5vZmYoKQ0KcG5nKCcuL2ZpbGVzL1BUX3NlbGJhbC8yMDIwLTA5LTIwX2JfY2Jfc2VsYmFsX2N2X2N5dG9faW1wcm92ZWRfcHJlcG9zdC5wbmcnLCB3aWR0aD0yNTAwLCBoZWlnaHQ9MjUwMCwgcmVzPTQwMCkNCmdyaWQuZHJhdyhjdl9jeXRvX2ltcHJvdmVkX3ByZXBvc3QkZ2xvYmFsLnBsb3QpDQpkZXYub2ZmKCkNCmBgYA0KDQpgYGB7cn0NCiMjd29yc2UgcHJlIHBvc3QNCg0KaW5kZXhfd29yc2VuZWQgPC0gYXMubnVtZXJpYyhnc3ViKCdBfEJ8IjAnLCcnLHJvd25hbWVzKGN5dG9fd29yc2VuZWQpKSkNCnByZXBvc3RfdmFyIDwtIGFzLmZhY3RvcihjeXRvX21hcF9pbXByb3ZlZF93b3JzZW5lZFtjeXRvX21hcF9pbXByb3ZlZF93b3JzZW5lZCRQSUQgJWluJSBpbmRleF93b3JzZW5lZCxdJENhc2Vfc3RhdHVzKQ0KcHJlcG9zdF92YXIgPC0gYXMuZmFjdG9yKGlmZWxzZShwcmVwb3N0X3ZhciA9PSAxLCAnMVByZScsICcyUG9zdCcpKQ0KUERwcmVfdmFyIDwtIGFzLm51bWVyaWMoY3l0b19tYXBfaW1wcm92ZWRfd29yc2VuZWRbY3l0b19tYXBfaW1wcm92ZWRfd29yc2VuZWQkUElEICVpbiUgaW5kZXhfd29yc2VuZWQsXSRQcmVQRHN1bSkNClBEcG9zdF92YXIgPC0gYXMubnVtZXJpYyhjeXRvX21hcF9pbXByb3ZlZF93b3JzZW5lZFtjeXRvX21hcF9pbXByb3ZlZF93b3JzZW5lZCRQSUQgJWluJSBpbmRleF93b3JzZW5lZCxdJFBvc3RQRHN1bSkNCg0KDQojc2VsYmFsIHRpbWUgd2V0DQpjdl9jeXRvX3dvcnNlbmVkX3ByZXBvc3QgPC0gc2VsYmFsLmN2KHg9Y3l0b193b3JzZW5lZCwgeT1wcmVwb3N0X3ZhciwgemVyby5yZXA9ImJheWVzIixzZWVkID0gMSxjb2wgPSBjKCIjMDA3MkIyIiwiI0Q1NUUwMCIpKQ0KY3ZfY3l0b193b3JzZW5lZF9wcmVwb3N0JGFjY3VyYWN5Lm52YXINCmN2X2N5dG9fd29yc2VuZWRfcHJlcG9zdCR2YXIuYmFycGxvdA0KZ3JpZC5kcmF3KGN2X2N5dG9fd29yc2VuZWRfcHJlcG9zdCRnbG9iYWwucGxvdCkNCnBsb3QudGFiKGN2X2N5dG9fd29yc2VuZWRfcHJlcG9zdCRjdi50YWIpDQpjdl9jeXRvX3dvcnNlbmVkX3ByZXBvc3QkZ2xtDQoNCiNtYWtlIHByZXR0eSBzZWxiYWwgIGN2Xw0KcG5nKCcuL2ZpbGVzL1BUX3NlbGJhbC8yMDIwLTA5LTIwX2NiX3NlbGJhbF9jdl9jeXRvX3dvcnNlbmVkX3ByZXBvc3QucG5nJywgd2lkdGg9NTAwMCwgaGVpZ2h0PTI5MDAsIHJlcz00MDApDQpncmlkLmRyYXcoY3ZfY3l0b193b3JzZW5lZF9wcmVwb3N0JGdsb2JhbC5wbG90KQ0KZGV2Lm9mZigpDQpwbmcoJy4vZmlsZXMvUFRfc2VsYmFsLzIwMjAtMDktMjBfY2Jfc2VsYmFsX2JfY3ZfY3l0b193b3JzZW5lZF9wcmVwb3N0LnBuZycsIHdpZHRoPTI1MDAsIGhlaWdodD0yNTAwLCByZXM9NDAwKQ0KZ3JpZC5kcmF3KGN2X2N5dG9fd29yc2VuZWRfcHJlcG9zdCRnbG9iYWwucGxvdCkNCmRldi5vZmYoKQ0KYGBgDQoNCmBgYHtyfQ0KDQojI2ltcHIgUEQNCmluZGV4X2ltcHJvdmVkIDwtIGFzLm51bWVyaWMoZ3N1YignQXxCfCIwJywnJyxyb3duYW1lcyhjeXRvX2ltcHJvdmVkKSkpDQpQRF92YXIgPC0gYXMubnVtZXJpYyhjeXRvX21hcF9pbXByb3ZlZF93b3JzZW5lZFtjeXRvX21hcF9pbXByb3ZlZF93b3JzZW5lZCRQSUQgJWluJSBpbmRleF9pbXByb3ZlZCxdJFBEU3VtKQ0KI3NlbGJhbCB0aW1lIHdldA0KY3ZfY3l0b19pbXByb3ZlZF9QRCA8LSBzZWxiYWwuY3YoeD1jeXRvX2ltcHJvdmVkLCB5PVBEX3ZhciwgemVyby5yZXA9ImJheWVzIixzZWVkID0gMSxjb2wgPSBjKCIjMDA3MkIyIiwiI0Q1NUUwMCIpKQ0KY3ZfY3l0b19pbXByb3ZlZF9QRCRhY2N1cmFjeS5udmFyDQpjdl9jeXRvX2ltcHJvdmVkX1BEJHZhci5iYXJwbG90DQpncmlkLmRyYXcoY3ZfY3l0b19pbXByb3ZlZF9QRCRnbG9iYWwucGxvdCkNCnBsb3QudGFiKGN2X2N5dG9faW1wcm92ZWRfUEQkY3YudGFiKQ0KY3ZfY3l0b19pbXByb3ZlZF9QRCRnbG0NCg0KI21ha2UgcHJldHR5IHNlbGJhbCAgY3ZfZGVwdGgNCnBuZygnLi9maWxlcy9QVF9zZWxiYWwvMjAyMC0wOS0yMF9jYl9zZWxiYWxfY3ZfY3l0b19pbXByb3ZlZF9QRC5wbmcnLCB3aWR0aD0yNTAwLCBoZWlnaHQ9MjUwMCwgcmVzPTQwMCkNCmdyaWQuZHJhdyhjdl9jeXRvX2ltcHJvdmVkX1BEJGdsb2JhbC5wbG90KQ0KZGV2Lm9mZigpDQpgYGANCg0KYGBge3J9DQojI3dvcnNlIFBEDQppbmRleF93b3JzZW5lZCA8LSBhcy5udW1lcmljKGdzdWIoJ0F8QnwiMCcsJycscm93bmFtZXMoY3l0b193b3JzZW5lZCkpKQ0KUERfdmFyIDwtIGFzLm51bWVyaWMoY3l0b19tYXBfaW1wcm92ZWRfd29yc2VuZWRbY3l0b19tYXBfaW1wcm92ZWRfd29yc2VuZWQkUElEICVpbiUgaW5kZXhfd29yc2VuZWQsXSRQRFN1bSkNCiNzZWxiYWwgdGltZSB3ZXQNCmN2X2N5dG9fd29yc2VuZWRfUEQgPC0gc2VsYmFsLmN2KHg9Y3l0b193b3JzZW5lZCwgeT1QRF92YXIsIHplcm8ucmVwPSJiYXllcyIsc2VlZCA9IDEsY29sID0gYygiIzAwNzJCMiIsIiNENTVFMDAiKSkNCmN2X2N5dG9fd29yc2VuZWRfUEQkYWNjdXJhY3kubnZhcg0KY3ZfY3l0b193b3JzZW5lZF9QRCR2YXIuYmFycGxvdA0KZ3JpZC5kcmF3KGN2X2N5dG9fd29yc2VuZWRfUEQkZ2xvYmFsLnBsb3QpDQpwbG90LnRhYihjdl9jeXRvX3dvcnNlbmVkX1BEJGN2LnRhYikNCmN2X2N5dG9fd29yc2VuZWRfUEQkZ2xtDQoNCiNtYWtlIHByZXR0eSBzZWxiYWwgIGN2X2RlcHRoDQpwbmcoJy4vZmlsZXMvUFRfc2VsYmFsLzIwMjAtMDktMjBfY2Jfc2VsYmFsX2N2X2N5dG9fd29yc2VuZWRfUEQucG5nJywgd2lkdGg9MjUwMCwgaGVpZ2h0PTI1MDAsIHJlcz00MDApDQpncmlkLmRyYXcoY3ZfY3l0b193b3JzZW5lZF9QRCRnbG9iYWwucGxvdCkNCmRldi5vZmYoKQ0KYGBgDQoNCg0KDQoNCmBgYHtyfQ0KI21ldGFnZW4gcHJlcG9zdA0KDQp2YXIgPC0gYXMuZmFjdG9yKG1ldGFnZW5fbWFwX29yZGVyZWQkUGVyaW9UcmVhdG1lbnQpDQp2YXIgPC0gYXMuZmFjdG9yKGlmZWxzZSh2YXIgPT0gJ1ByZScsICcxUHJlJywgJzJQb3N0JykpDQpzZWxiYWxfdCA8LSBtZXRhZ2VuX2dlbnVzX3QNCnJvd25hbWVzKHNlbGJhbF90KSA8LSBnc3ViKCcua3Jha2VuJywnJyxyb3duYW1lcyhzZWxiYWxfdCkpDQpucm93KHNlbGJhbF90KQ0KbGVuZ3RoKHZhcikNCiNzZWxiYWwgdGltZSB3ZXQNCmN2X21ldGFnZW5fcHJlcG9zdCA8LSBzZWxiYWwuY3YoeD1zZWxiYWxfdCwgeT12YXIsIHplcm8ucmVwPSJiYXllcyIsc2VlZCA9IDEsY29sID0gYygiIzAwNzJCMiIsIiNENTVFMDAiKSkNCmN2X21ldGFnZW5fcHJlcG9zdCRhY2N1cmFjeS5udmFyDQpjdl9tZXRhZ2VuX3ByZXBvc3QkdmFyLmJhcnBsb3QNCmdyaWQuZHJhdyhjdl9tZXRhZ2VuX3ByZXBvc3QkZ2xvYmFsLnBsb3QpDQpwbG90LnRhYihjdl9tZXRhZ2VuX3ByZXBvc3QkY3YudGFiKQ0KY3ZfbWV0YWdlbl9wcmVwb3N0JGdsbQ0KDQojbWFrZSBwcmV0dHkgc2VsYmFsICBjdl8NCnBuZygnLi9maWxlcy9QVF9zZWxiYWwvMjAyMC0wOS0yMF9jYl9zZWxiYWxfY3ZfbWV0YWdlbl9wcmVwb3N0LnBuZycsIHdpZHRoPTUwMDAsIGhlaWdodD0yOTAwLCByZXM9NDAwKQ0KZ3JpZC5kcmF3KGN2X21ldGFnZW5fcHJlcG9zdCRnbG9iYWwucGxvdCkNCmRldi5vZmYoKQ0KcG5nKCcuL2ZpbGVzL1BUX3NlbGJhbC8yMDIwLTA5LTIwX2NiX3NlbGJhbF9jdl9tZXRhZ2VuX3ByZXBvc3QucG5nJywgd2lkdGg9MjUwMCwgaGVpZ2h0PTI1MDAsIHJlcz00MDApDQpncmlkLmRyYXcoY3ZfbWV0YWdlbl9wcmVwb3N0JGdsb2JhbC5wbG90KQ0KZGV2Lm9mZigpDQpgYGANCg0KDQpgYGB7cn0NCiNtZXRhZ2VuIE9SDQoNCnZhciA8LSBhcy5mYWN0b3IobWV0YWdlbl9tYXBfb3JkZXJlZCRPdmVyYWxsUmVzcG9uc2UpDQojdmFyIDwtIGFzLmZhY3RvcihpZmVsc2UodmFyID09ICdQcmUnLCAnMVByZScsICcyUG9zdCcpKQ0Kc2VsYmFsX3QgPC0gbWV0YWdlbl9nZW51c190DQpyb3duYW1lcyhzZWxiYWxfdCkgPC0gZ3N1YignLmtyYWtlbicsJycscm93bmFtZXMoc2VsYmFsX3QpKQ0KbnJvdyhzZWxiYWxfdCkNCmxlbmd0aCh2YXIpDQojc2VsYmFsIHRpbWUgd2V0DQpjdl9tZXRhZ2VuX09SIDwtIHNlbGJhbC5jdih4PXNlbGJhbF90LCB5PXZhciwgemVyby5yZXA9ImJheWVzIixzZWVkID0gMSxjb2wgPSBjKCIjMDA3MkIyIiwiI0Q1NUUwMCIpKQ0KY3ZfbWV0YWdlbl9PUiRhY2N1cmFjeS5udmFyDQpjdl9tZXRhZ2VuX09SJGJhcnBsb3QNCmdyaWQuZHJhdyhjdl9tZXRhZ2VuX09SJGdsb2JhbC5wbG90KQ0KcGxvdC50YWIoY3ZfbWV0YWdlbl9PUiRjdi50YWIpDQpjdl9tZXRhZ2VuX09SJGdsbQ0KDQojbWFrZSBwcmV0dHkgc2VsYmFsICBjdl8NCnBuZygnLi9maWxlcy9QVF9zZWxiYWwvMjAyMC0wOS0yMF9jYl9zZWxiYWxfY3ZfbWV0YWdlbl9PUi5wbmcnLCB3aWR0aD01MDAwLCBoZWlnaHQ9MjkwMCwgcmVzPTQwMCkNCmdyaWQuZHJhdyhjdl9tZXRhZ2VuX09SJGdsb2JhbC5wbG90KQ0KZGV2Lm9mZigpDQpwbmcoJy4vZmlsZXMvUFRfc2VsYmFsLzIwMjAtMDktMjBfYl9jYl9zZWxiYWxfY3ZfbWV0YWdlbl9PUi5wbmcnLCB3aWR0aD0yNTAwLCBoZWlnaHQ9MjUwMCwgcmVzPTQwMCkNCmdyaWQuZHJhdyhjdl9tZXRhZ2VuX09SJGdsb2JhbC5wbG90KQ0KZGV2Lm9mZigpDQpgYGANCg0KYGBge3J9DQojbWV0YWdlbiBwZHN1bQ0KdmFyIDwtIGFzLm51bWVyaWMoYmFjdF9tYXBfaW1wcm92ZWRfd29yc2VuZWRbZ3N1YignLmtyYWtlbicsJycscm93bmFtZXMobWV0YWdlbl9nZW51c190KSksXSRQRHN1bSkNCiN2YXIgPC0gYXMuZmFjdG9yKGlmZWxzZSh2YXIgPT0gJ1ByZScsICcxUHJlJywgJzJQb3N0JykpDQpzZWxiYWxfdCA8LSBtZXRhZ2VuX2dlbnVzX3QNCnJvd25hbWVzKHNlbGJhbF90KSA8LSBnc3ViKCcua3Jha2VuJywnJyxyb3duYW1lcyhzZWxiYWxfdCkpDQpucm93KHNlbGJhbF90KQ0KbGVuZ3RoKHZhcikNCiNzZWxiYWwgdGltZSB3ZXQNCmN2X21ldGFnZW5fUEQgPC0gc2VsYmFsLmN2KHg9c2VsYmFsX3QsIHk9dmFyLCB6ZXJvLnJlcD0iYmF5ZXMiLHNlZWQgPSAxLGNvbCA9IGMoIiMwMDcyQjIiLCIjRDU1RTAwIikpDQpjdl9tZXRhZ2VuX1BEJGFjY3VyYWN5Lm52YXINCmN2X21ldGFnZW5fUEQkdmFyLmJhcnBsb3QNCmdyaWQuZHJhdyhjdl9tZXRhZ2VuX1BEJGdsb2JhbC5wbG90KQ0KcGxvdC50YWIoY3ZfbWV0YWdlbl9QRCRjdi50YWIpDQpjdl9tZXRhZ2VuX1BEJGdsbQ0KDQojbWFrZSBwcmV0dHkgc2VsYmFsICBjdl8NCnBuZygnLi9maWxlcy9QVF9zZWxiYWwvMjAyMC0wOS0yMF9jYl9zZWxiYWxfY3ZfbWV0YWdlbl9QRC5wbmcnLCB3aWR0aD0zNTAwLCBoZWlnaHQ9MzUwMCwgcmVzPTQwMCkNCmdyaWQuZHJhdyhjdl9tZXRhZ2VuX1BEJGdsb2JhbC5wbG90KQ0KZGV2Lm9mZigpDQpgYGANCg0KYGBge3J9DQojbWV0YWdlbiBpbXByb3ZlZCBwcmVwb3N0DQp2YXIgPC0gYXMuZmFjdG9yKG1ldGFnZW5fbWFwX29yZGVyZWRbbWV0YWdlbl9tYXBfb3JkZXJlZCRPdmVyYWxsUmVzcG9uc2U9PSdJbXByb3ZlZCcsXSRQZXJpb1RyZWF0bWVudCkNCnZhciA8LSBhcy5mYWN0b3IoaWZlbHNlKHZhciA9PSAnUHJlJywgJzFQcmUnLCAnMlBvc3QnKSkNCnNlbGJhbF90IDwtIG1ldGFnZW5faW1wcm92ZWQNCnJvd25hbWVzKHNlbGJhbF90KSA8LSBnc3ViKCcua3Jha2VuJywnJyxyb3duYW1lcyhzZWxiYWxfdCkpDQpucm93KHNlbGJhbF90KQ0KbGVuZ3RoKHZhcikNCiNzZWxiYWwgdGltZSB3ZXQNCmN2X21ldGFnZW5faW1wX3ByZXBvc3QgPC0gc2VsYmFsLmN2KHg9c2VsYmFsX3Qsbi5mb2xkID0gMywgeT12YXIsc2VlZD0xLCB6ZXJvLnJlcD0ib25lIixjb2wgPSBjKCIjMDA3MkIyIiwiI0Q1NUUwMCIpKQ0KY3ZfbWV0YWdlbl9pbXBfcHJlcG9zdCRhY2N1cmFjeS5udmFyDQpjdl9tZXRhZ2VuX2ltcF9wcmVwb3N0JHZhci5iYXJwbG90DQpncmlkLmRyYXcoY3ZfbWV0YWdlbl9pbXBfcHJlcG9zdCRnbG9iYWwucGxvdCkNCnBsb3QudGFiKGN2X21ldGFnZW5faW1wX3ByZXBvc3QkY3YudGFiKQ0KY3ZfbWV0YWdlbl9pbXBfcHJlcG9zdCRnbG0NCg0KI21ha2UgcHJldHR5IHNlbGJhbCAgY3ZfDQpwbmcoJy4vZmlsZXMvUFRfc2VsYmFsLzIwMjAtMDktMjBfY2Jfc2VsYmFsX2N2X21ldGFnZW5faW1wcm92ZWRfcHJlcG9zdC5wbmcnLCB3aWR0aD01MDAwLCBoZWlnaHQ9MjkwMCwgcmVzPTQwMCkNCmdyaWQuZHJhdyhjdl9tZXRhZ2VuX2ltcF9wcmVwb3N0JGdsb2JhbC5wbG90KQ0KZGV2Lm9mZigpDQojbWFrZSBwcmV0dHkgc2VsYmFsICBjdl8NCnBuZygnLi9maWxlcy9QVF9zZWxiYWwvMjAyMC0wOS0yMF9iX2NiX3NlbGJhbF9jdl9tZXRhZ2VuX2ltcHJvdmVkX3ByZXBvc3QucG5nJywgd2lkdGg9MjUwMCwgaGVpZ2h0PTI1MDAsIHJlcz00MDApDQpncmlkLmRyYXcoY3ZfbWV0YWdlbl9pbXBfcHJlcG9zdCRnbG9iYWwucGxvdCkNCmRldi5vZmYoKQ0KYGBgDQoNCmBgYHtyfQ0KI21ldGFnZW4gd29yc2VuZWQgcHJlcG9zdA0KdmFyIDwtIGFzLmZhY3RvcihtZXRhZ2VuX21hcF9vcmRlcmVkW21ldGFnZW5fbWFwX29yZGVyZWQkT3ZlcmFsbFJlc3BvbnNlPT0nV29yc2VuZWQnLF0kUGVyaW9UcmVhdG1lbnQpDQp2YXIgPC0gYXMuZmFjdG9yKGlmZWxzZSh2YXIgPT0gJ1ByZScsICcxUHJlJywgJzJQb3N0JykpDQpzZWxiYWxfdCA8LSBtZXRhZ2VuX3dvcnNlbmVkDQpyb3duYW1lcyhzZWxiYWxfdCkgPC0gZ3N1YignLmtyYWtlbicsJycscm93bmFtZXMoc2VsYmFsX3QpKQ0KbnJvdyhzZWxiYWxfdCkNCmxlbmd0aCh2YXIpDQojc2VsYmFsIHRpbWUgd2V0DQpjdl9tZXRhZ2VuX3dvcl9wcmVwb3N0IDwtIHNlbGJhbC5jdih4PXNlbGJhbF90LCB5PXZhciwgemVyby5yZXA9Im9uZSIsc2VlZCA9IDEsY29sID0gYygiIzAwNzJCMiIsIiNENTVFMDAiKSkNCmN2X21ldGFnZW5fd29yX3ByZXBvc3QkYWNjdXJhY3kubnZhcg0KY3ZfbWV0YWdlbl93b3JfcHJlcG9zdCR2YXIuYmFycGxvdA0KZ3JpZC5kcmF3KGN2X21ldGFnZW5fd29yX3ByZXBvc3QkZ2xvYmFsLnBsb3QpDQpwbG90LnRhYihjdl9tZXRhZ2VuX3dvcl9wcmVwb3N0JGN2LnRhYikNCmN2X21ldGFnZW5fd29yX3ByZXBvc3QkZ2xtDQoNCiNtYWtlIHByZXR0eSBzZWxiYWwgIGN2Xw0KcG5nKCcuL2ZpbGVzL1BUX3NlbGJhbC8yMDIwLTA5LTIwX2NiX3NlbGJhbF9jdl9tZXRhZ2VuX3dvcnNlbmVkX3ByZXBvc3QucG5nJywgd2lkdGg9NTAwMCwgaGVpZ2h0PTI5MDAsIHJlcz00MDApDQpncmlkLmRyYXcoY3ZfbWV0YWdlbl93b3JfcHJlcG9zdCRnbG9iYWwucGxvdCkNCmRldi5vZmYoKQ0KcG5nKCcuL2ZpbGVzL1BUX3NlbGJhbC8yMDIwLTA5LTIwX2JfY2Jfc2VsYmFsX2N2X21ldGFnZW5fd29yc2VuZWRfcHJlcG9zdC5wbmcnLCB3aWR0aD0yNTAwLCBoZWlnaHQ9MjUwMCwgcmVzPTQwMCkNCmdyaWQuZHJhdyhjdl9tZXRhZ2VuX3dvcl9wcmVwb3N0JGdsb2JhbC5wbG90KQ0KZGV2Lm9mZigpDQpgYGANCg0KYGBge3J9DQojbWV0YWdlbiBpbXByb3ZlZCBQRA0KI21ldGFnZW5fZ2VudXNfdA0KdmFyIDwtIGFzLm51bWVyaWMoYmFjdF9tYXBfaW1wcm92ZWRfd29yc2VuZWRbZ3N1YignLmtyYWtlbicsJycscm93bmFtZXMobWV0YWdlbl9pbXByb3ZlZCkpLF0kUERzdW0pDQojIHZhciA8LSBhcy5mYWN0b3IoaWZlbHNlKHZhciA9PSAnUHJlJywgJzFQcmUnLCAnMlBvc3QnKSkNCnNlbGJhbF90IDwtIG1ldGFnZW5faW1wcm92ZWQNCnJvd25hbWVzKHNlbGJhbF90KSA8LSBnc3ViKCcua3Jha2VuJywnJyxyb3duYW1lcyhzZWxiYWxfdCkpDQpucm93KHNlbGJhbF90KQ0KbGVuZ3RoKHZhcikNCiNzZWxiYWwgdGltZSB3ZXQNCmN2X21ldGFnZW5faW1wX1BEIDwtIHNlbGJhbC5jdih4PXNlbGJhbF90LG4uZm9sZCA9IDMsIHk9dmFyLCB6ZXJvLnJlcD0ib25lIixzZWVkID0gMSxjb2wgPSBjKCIjMDA3MkIyIiwiI0Q1NUUwMCIpKQ0KY3ZfbWV0YWdlbl9pbXBfUEQkYWNjdXJhY3kubnZhcg0KY3ZfbWV0YWdlbl9pbXBfUEQkdmFyLmJhcnBsb3QNCmdyaWQuZHJhdyhjdl9tZXRhZ2VuX2ltcF9QRCRnbG9iYWwucGxvdCkNCnBsb3QudGFiKGN2X21ldGFnZW5faW1wX1BEJGN2LnRhYikNCmN2X21ldGFnZW5faW1wX1BEJGdsbQ0KDQojbWFrZSBwcmV0dHkgc2VsYmFsICBjdl8NCg0KcG5nKCcuL2ZpbGVzL1BUX3NlbGJhbC8yMDIwLTA5LTIwX2NiX3NlbGJhbF9jdl9tZXRhZ2VuX2ltcHJvdmVkX1BELnBuZycsIHdpZHRoPTI1MDAsIGhlaWdodD0yNTAwLCByZXM9NDAwKQ0KZ3JpZC5kcmF3KGN2X21ldGFnZW5faW1wX1BEJGdsb2JhbC5wbG90KQ0KZGV2Lm9mZigpDQoNCmBgYA0KDQpgYGB7cn0NCiNtZXRhZ2VuIHdvcnNlbmVkIFBEDQojbWV0YWdlbl9nZW51c190DQp2YXIgPC0gYXMubnVtZXJpYyhiYWN0X21hcF9pbXByb3ZlZF93b3JzZW5lZFtnc3ViKCcua3Jha2VuJywnJyxyb3duYW1lcyhtZXRhZ2VuX3dvcnNlbmVkKSksXSRQRHN1bSkNCiMgdmFyIDwtIGFzLmZhY3RvcihpZmVsc2UodmFyID09ICdQcmUnLCAnMVByZScsICcyUG9zdCcpKQ0Kc2VsYmFsX3QgPC0gbWV0YWdlbl93b3JzZW5lZA0Kcm93bmFtZXMoc2VsYmFsX3QpIDwtIGdzdWIoJy5rcmFrZW4nLCcnLHJvd25hbWVzKHNlbGJhbF90KSkNCm5yb3coc2VsYmFsX3QpDQpsZW5ndGgodmFyKQ0KI3NlbGJhbCB0aW1lIHdldA0KY3ZfbWV0YWdlbl93b3JfUEQgPC0gc2VsYmFsLmN2KHg9c2VsYmFsX3Qsbi5mb2xkID0gMywgeT12YXIsc2VlZD0xLCB6ZXJvLnJlcD0ib25lIixjb2wgPSBjKCIjMDA3MkIyIiwiI0Q1NUUwMCIpKQ0KY3ZfbWV0YWdlbl93b3JfUEQkYWNjdXJhY3kubnZhcg0KY3ZfbWV0YWdlbl93b3JfUEQkdmFyLmJhcnBsb3QNCmdyaWQuZHJhdyhjdl9tZXRhZ2VuX3dvcl9QRCRnbG9iYWwucGxvdCkNCnBsb3QudGFiKGN2X21ldGFnZW5fd29yX1BEJGN2LnRhYikNCmN2X21ldGFnZW5fd29yX1BEJGdsbQ0KDQojbWFrZSBwcmV0dHkgc2VsYmFsICBjdl8NCnBuZygnLi9maWxlcy9QVF9zZWxiYWwvMjAyMC0wOS0yMF9jYl9zZWxiYWxfY3ZfbWV0YWdlbl93b3JzZW5lZF9QRHN1bS5wbmcnLCB3aWR0aD0yNTAwLCBoZWlnaHQ9MjUwMCwgcmVzPTQwMCkNCmdyaWQuZHJhdyhjdl9tZXRhZ2VuX3dvcl9QRCRnbG9iYWwucGxvdCkNCmRldi5vZmYoKQ0KYGBgDQoNCg0KDQoNCg0KDQoNCg0KDQoNCg0KDQoNCg0KDQpTSFQgc2VsYmFsIGFuYWx5c2lzDQoNCmBgYHtyfQ0KI1NIVCBkYXRhIHJlYWQgaW4gDQojcmVhZCBpbiBkYXRhDQpiYWN0X2RmX29yZGVyZWQgPC0gcmVhZC50YWJsZSgnLi9maWxlcy8yMDIwLTA5LTIwX1NIVF8xNlNfT1RVX2dlbnVzX1RhYmwuY3N2JyxoZWFkZXIgPSAxLHJvdy5uYW1lcyA9IDEsc2VwPScsJyxjaGVjay5uYW1lcz1GQUxTRSkNCmJhY3RfZ2VudXMxMF9yZWRzbGltIDwtYmFjdF9kZl9vcmRlcmVkDQptZXRhYl9kZiA8LSByZWFkLnRhYmxlKCcuL2ZpbGVzLzIwMjAtMDktMjBfU0hUX21ldGFiX3RhYmxlLmNzdicsc2VwPScsJyxoZWFkZXIgPTEscm93Lm5hbWVzID0gMSxjaGVjay5uYW1lcyA9IEYsbmEuc3RyaW5ncyA9ICdORCcpDQptZXRhYl91bnR4X3JlZCA8LSBtZXRhYl9kZltyb3dTdW1zKG1ldGFiX2RmIDwxMDAwMDAwKSA8PSAwLjkwICogbmNvbChtZXRhYl9kZiksIF0NCm1ldGFnZW5fZGZfb3JkZXJlZCA8LSByZWFkLnRhYmxlKCcuL2ZpbGVzLzIwMjAtMDktMjBfU0hUX21ldGFnZW5fZ2VudXNfdGFibGUuY3N2JyxzZXA9JywnLHJvdy5uYW1lcyA9IDEsaGVhZGVyID0gMSxjaGVjay5uYW1lcz1GQUxTRSkNCm1ldGFnZW5fZ2VudXNfcmVkIDwtIG1ldGFnZW5fZGZfb3JkZXJlZA0KDQpgYGANCg0KDQpgYGB7cn0NCiNpbXBvcnQgc2FtcGxlIG1hcHMNCmJhY3RfbWFwX29yZGVyZWQgPC0gcmVhZC50YWJsZSgnLi9maWxlcy8yMDIwLTA4LTExX1NIVF9tYXBwaW5nZmlsZS5jc3YnLHNlcD0nLCcsaGVhZGVyID0xLHJvdy5uYW1lcyA9IDEsY2hlY2submFtZXMgPSBGKQ0KDQptZXRhZ2VuX21hcDwtIHJlYWQudGFibGUoJy4vZmlsZXMvTWV0YWRhdGFfYW5kX3BlcmlvX05BLnR4dCcsc2VwPSdcdCcsaGVhZGVyID0xLHJvdy5uYW1lcyA9IDEpDQptZXRhZ2VuX21hcF9vcmRlcmVkIDwtIG1ldGFnZW5fbWFwW29yZGVyKHJvdy5uYW1lcyhtZXRhZ2VuX21hcCkpLF0NCg0KYGBgDQoNCg0KYGBge3J9DQojICNkaWRlYXNlIGNsYXNzIA0KIyAjcG9ja2V0IGRlcHRoIA0KDQojIyN3aGljaCBzYW1wbGVzIHRvIGtlZXAgICNkZWwgUEEgYW5kIE5BIHBvY2tldGRlcHRoDQpiYWN0X21hcF9ub1BhIDwtIGJhY3RfbWFwX29yZGVyZWRbZ3JlcCggJ0J8Yicscm93Lm5hbWVzKGJhY3RfbWFwX29yZGVyZWQpKSxdDQojIGJhY3RfbWFwX25vUGFfQUJDIDwtIGJhY3RfbWFwX25vUGFbZ3JlcCgnQXxCfEMnLGJhY3RfbWFwX25vUGEkZGlzZWFzY2xhc3MpLF0NCmJhY3RfZGZfbm9QYSA8LSBiYWN0X2RmX29yZGVyZWRbLGdyZXAoJ0J8YicsY29sbmFtZXMoYmFjdF9kZl9vcmRlcmVkKSldDQpiYWN0X21hcF9ub1BhIDwtIGJhY3RfbWFwX29yZGVyZWRbcm93bmFtZXMoYmFjdF9tYXBfb3JkZXJlZCkgJWluJSBjb2xuYW1lcyhiYWN0X2RmX25vUGEpLF0NCmJhY3RfbWFwX25vUGEgPC0gYmFjdF9tYXBfbm9QYVshaXMubmEoYXMubnVtZXJpYyhiYWN0X21hcF9ub1BhJHBvY2tldGRlcHRoKSksXQ0KYmFjdF9tYXBfbm9QYV9BQyA8LSBiYWN0X21hcF9ub1BhW2dyZXAoJ0F8QycsYmFjdF9tYXBfbm9QYSRkaXNlYXNjbGFzcyksXQ0KYmFjdF9kZl9ub1BhX0FDIDwtIGJhY3RfZGZfbm9QYVssYmFjdF9tYXBfbm9QYV9BQyREZXNjcmlwdGlvbl0NCg0KbWV0YWJfbm9QYSA8LSBtZXRhYl9kZlssZ3JlcCgnYnxCJyxjb2xuYW1lcyhtZXRhYl9kZikpXQ0KbWV0YWJfbWFwX25vUGEgPC0gYmFjdF9tYXBfb3JkZXJlZFtiYWN0X21hcF9vcmRlcmVkJG1ldGFiX2Rlc2NyaXB0aW9uICVpbiUgY29sbmFtZXMobWV0YWJfbm9QYSksXSMNCm1ldGFiX21hcF9ub1BhIDwtIG1ldGFiX21hcF9ub1BhWyFpcy5uYShhcy5udW1lcmljKG1ldGFiX21hcF9ub1BhJHBvY2tldGRlcHRoKSksXQ0KbWV0YWJfbWFwX25vUGFfQUMgPC0gbWV0YWJfbWFwX25vUGFbZ3JlcCgnQXxDJyxtZXRhYl9tYXBfbm9QYSRkaXNlYXNjbGFzcyksXQ0KIyBtZXRhYl9ub1BhX0FDIDwtIG1ldGFiX25vUGFbLGNvbG5hbWVzKG1ldGFiX25vUGEpJWluJXJvd25hbWVzKG1ldGFiX21hcF9ub1BhX0FDKV0NCg0KIyBzZXR1cA0KI2ZpbHRlciBtZXRhZ2VuDQojIG1ldGFnZW5fZGZfZmlsdGVyZWQgPC0gbWV0YWdlbl9kZl9vcmRlcmVkWyxjb2xuYW1lcyhtZXRhZ2VuX2RmX29yZGVyZWQpJWluJXJvdy5uYW1lcyhtZXRhZ2VuX21hcF9vcmRlcmVkKV0gDQptZXRhZ2VuX21hcF9ub1BhIDwtIG1ldGFnZW5fbWFwX29yZGVyZWRbZ3JlcCgnQnxiJyxyb3cubmFtZXMobWV0YWdlbl9tYXBfb3JkZXJlZCkpLF0NCm1ldGFnZW5fbWFwX25vUGFfQUMgPC0gbWV0YWdlbl9tYXBfbm9QYVtncmVwKCdBfEMnLG1ldGFnZW5fbWFwX25vUGEkRGlzZWFzQ2xhc3MpLF0NCm1ldGFnZW5fbm9QYV9BQyA8LSBtZXRhZ2VuX2RmX2ZpbHRlcmVkWyxjb2xuYW1lcyhtZXRhZ2VuX2RmX2ZpbHRlcmVkKSVpbiVyb3duYW1lcyhtZXRhZ2VuX21hcF9ub1BhX0FDKVtncmVwKCdCfGInLHJvd25hbWVzKG1ldGFnZW5fbWFwX25vUGFfQUMpKV1dDQpgYGANCg0KDQoNCmBgYHtyfQ0KIyMjIyMjU0VMQkFMDQojIG1ldGFnZW5fdW50eF9zdW0NCiMgYmFjdF9nZW51czEwX3JlZHNsaW0NCiMgbWV0YWJfdW50eF9yZWQNCiMgIyBtYXBfdW50eA0KIyAjIG1ldGFiX3VudHhfcmVkYg0KIyBtZXRhZ2VuX2dlbnVzX3JlZA0KDQojIyN3aGljaCBzYW1wbGVzIHRvIGtlZXAgICNkZWwgUEEgYmFzZWQgb24gbWFwIHVzZWQgb24gYWxsIGFuYWx5c2lzDQojIGJhY3RfbWFwX25vUGE8LWJhY3RfbWFwX29yZGVyZWRbZ3JlcCggJ0J8Yicscm93Lm5hbWVzKGJhY3RfbWFwX29yZGVyZWQpKSxdDQpiYWN0X2RmX25vUGEgPC0gYmFjdF9nZW51czEwX3JlZHNsaW1bLGdyZXAoJ0J8YicsY29sbmFtZXMoYmFjdF9nZW51czEwX3JlZHNsaW0pKV0NCmJhY3RfZGZfbm9QYV9BQyA8LSBiYWN0X2RmX25vUGFbLGJhY3RfbWFwX25vUGFfQUMkRGVzY3JpcHRpb25dDQoNCm1ldGFiX25vUGEgPC0gbWV0YWJfdW50eF9yZWRbLGdyZXAoJ2J8QicsY29sbmFtZXMobWV0YWJfdW50eF9yZWQpKV0NCm1ldGFiX25vUGFfQUMgPC0gbWV0YWJfbm9QYVssZ3N1YignQicsJy5CJyxjb2xuYW1lcyhtZXRhYl9ub1BhKSklaW4lcm93bmFtZXMobWV0YWJfbWFwX25vUGFfQUMpXQ0KDQojZmlsdGVyIG1ldGFnZW4NCm1ldGFnZW5fZGZfZmlsdGVyZWQgPC0gbWV0YWdlbl9nZW51c19yZWRbLHJvdy5uYW1lcyhtZXRhZ2VuX21hcF9ub1BhX0FDKV0gDQptZXRhZ2VuX21hcF9ub1BhX0FDIDwtIG1ldGFnZW5fbWFwX25vUGFfQUNbZ3JlcCgnQnxiJyxyb3cubmFtZXMobWV0YWdlbl9tYXBfbm9QYV9BQykpLF0NCm1ldGFnZW5fbm9QYSA8LSBtZXRhZ2VuX2RmX2ZpbHRlcmVkWyxncmVwKCdCfGInLGNvbG5hbWVzKG1ldGFnZW5fZGZfZmlsdGVyZWQpKV0NCmBgYA0KDQpgYGB7cn0NCg0KI2JhY3Qgc2VsYmFsIFBDIEEgQw0KYmFjdF9tYXBfbm9QYV9BQyA8LSBiYWN0X21hcF9ub1BhX0FDW2dyZXAoJ0F8QycsYmFjdF9tYXBfbm9QYV9BQyRkaXNlYXNjbGFzcyksXQ0KYmFjdF9kZl9ub1BhX0FDIDwtYmFjdF9kZl9ub1BhX0FDWyxiYWN0X21hcF9ub1BhX0FDJERlc2NyaXB0aW9uXQ0KDQp2YXJfYmFjdCA8LSBhcy5mYWN0b3IoYmFjdF9tYXBfbm9QYV9BQyRkaXNlYXNjbGFzcykNCg0KI3NlbGJhbCBuZWVkcyB0d28gaW5wdXRzLSBtYXRyaXggd2l0aCBzYW1wbGVzIGFzIHJvd3MgYW5kIHRheGEgYXMgY29sdW1uDQojc2V0dXAgDQpiYWN0X2dlbnVzX3N1bV90IDwtIHQoYmFjdF9kZl9ub1BhX0FDKSMNCg0KY3ZfYmFjdF9BdkNfREMgPC0gc2VsYmFsLmN2KHg9YmFjdF9nZW51c19zdW1fdCwgeT12YXJfYmFjdCwgemVyby5yZXA9ImJheWVzIixzZWVkID0gMSxjb2wgPSBjKCIjMDA3MkIyIiwiI0Q1NUUwMCIpKQ0KY3ZfYmFjdF9BdkNfREMkYWNjdXJhY3kubnZhcg0KY3ZfYmFjdF9BdkNfREMkdmFyLmJhcnBsb3QNCmdyaWQuZHJhdyhjdl9iYWN0X0F2Q19EQyRnbG9iYWwucGxvdCkNCnBsb3QudGFiKGN2X2JhY3RfQXZDX0RDJGN2LnRhYikNCmN2X2JhY3RfQXZDX0RDJGdsbQ0KDQojbWFrZSBwcmV0dHkgc2VsYmFsDQpwbmcoJy4vZmlsZXMvU0hUX3NlbGJhbC8yMDIwLTA5LTIwX2NiX3NlbGJhbF9jdl9iYWN0X0RDX0FDLnBuZycsIHdpZHRoPTUwMDAsIGhlaWdodD0yOTAwLCByZXM9NDAwKQ0KZ3JpZC5kcmF3KGN2X2JhY3RfQXZDX0RDJGdsb2JhbC5wbG90KQ0KZGV2Lm9mZigpDQoNCnBuZygnLi9maWxlcy9TSFRfc2VsYmFsLzIwMjAtMDktMjBfYl9jYl9zZWxiYWxfY3ZfYmFjdF9EQ19BQy5wbmcnLCB3aWR0aD0yNTAwLCBoZWlnaHQ9MjUwMCwgcmVzPTMzMykNCmdyaWQuZHJhdyhjdl9iYWN0X0F2Q19EQyRnbG9iYWwucGxvdCkNCmRldi5vZmYoKQ0KYGBgDQoNCmBgYHtyfQ0KDQojYmFjdCBzZWxiYWwgUEMgQUMgcG9ja2V0IGRlcHRoDQpiYWN0X21hcF9ub1BhX0MgPC0gYmFjdF9tYXBfbm9QYV9BQ1tncmVwKCdDJyxiYWN0X21hcF9ub1BhX0FDJGRpc2Vhc2NsYXNzKSxdDQpiYWN0X2RmX25vUGFfQyA8LWJhY3RfZGZfbm9QYV9BQ1sscm93bmFtZXMoYmFjdF9tYXBfbm9QYV9DKV0NCg0KdmFyX2JhY3QgPC0gYXMubnVtZXJpYyhiYWN0X21hcF9ub1BhX0MkcG9ja2V0ZGVwdGgpDQpiYWN0X2RmX25vUGFfQyA8LSBiYWN0X2RmX25vUGFfQ1ssIWlzLm5hKHZhcl9iYWN0KV0NCnZhcl9iYWN0IDwtIHZhcl9iYWN0WyFpcy5uYSh2YXJfYmFjdCldDQojc2VsYmFsIG5lZWRzIHR3byBpbnB1dHMtIG1hdHJpeCB3aXRoIHNhbXBsZXMgYXMgcm93cyBhbmQgdGF4YSBhcyBjb2x1bW4NCiNzZXR1cCANCmJhY3RfZ2VudXNfc3VtX3QgPC0gdChiYWN0X2RmX25vUGFfQykjDQp0YWJsZShiYWN0X2dlbnVzX3N1bV90PT0wKQ0KDQpjdl9iYWN0X1BEX0MgPC0gc2VsYmFsLmN2KHg9YmFjdF9nZW51c19zdW1fdCwgeT12YXJfYmFjdCwgemVyby5yZXA9Im9uZSIsc2VlZCA9IDEsY29sID0gYygiIzAwNzJCMiIsIiNENTVFMDAiKSkNCmN2X2JhY3RfUERfQyRhY2N1cmFjeS5udmFyDQpjdl9iYWN0X1BEX0MkdmFyLmJhcnBsb3QNCmdyaWQuZHJhdyhjdl9iYWN0X1BEX0MkZ2xvYmFsLnBsb3QpDQpwbG90LnRhYihjdl9iYWN0X1BEX0MkY3YudGFiKQ0KY3ZfYmFjdF9QRF9DJGdsbQ0KDQojbWFrZSBwcmV0dHkgc2VsYmFsICBjdl8NCnBuZygnLi9maWxlcy9TSFRfc2VsYmFsLzIwMjAtMDktMjBfY2Jfc2VsYmFsX2N2X2JhY3RfUERfQy5wbmcnLCB3aWR0aD0yNTAwLCBoZWlnaHQ9MjUwMCwgcmVzPTQwMCkNCmdyaWQuZHJhdyhjdl9iYWN0X1BEX0MkZ2xvYmFsLnBsb3QpDQpkZXYub2ZmKCkNCmBgYA0KDQpgYGB7cn0NCiNiYWN0IHNlbGJhbCBQQyBBIHBvY2tldCBkZXB0aA0KYmFjdF9tYXBfbm9QYV9BIDwtIGJhY3RfbWFwX25vUGFbZ3JlcCgnQScsYmFjdF9tYXBfbm9QYSRkaXNlYXNjbGFzcyksXQ0KYmFjdF9kZl9ub1BhX0EgPC1iYWN0X2RmX25vUGFbLHJvd25hbWVzKGJhY3RfbWFwX25vUGFfQSldDQoNCnZhcl9iYWN0IDwtIGFzLm51bWVyaWMoYmFjdF9tYXBfbm9QYV9BJHBvY2tldGRlcHRoKQ0KYmFjdF9kZl9ub1BhX0EgPC0gYmFjdF9kZl9ub1BhX0FbLCFpcy5uYSh2YXJfYmFjdCldDQp2YXJfYmFjdCA8LSB2YXJfYmFjdFshaXMubmEodmFyX2JhY3QpXQ0KI3NlbGJhbCBuZWVkcyB0d28gaW5wdXRzLSBtYXRyaXggd2l0aCBzYW1wbGVzIGFzIHJvd3MgYW5kIHRheGEgYXMgY29sdW1uDQojc2V0dXAgDQpiYWN0X2dlbnVzX3N1bV90IDwtIHQoYmFjdF9kZl9ub1BhX0EpIw0KdGFibGUoYmFjdF9nZW51c19zdW1fdD09MCkNCiNzZWxiYWwgcHJlIHJlc3ANCmN2X2JhY3RfUERfQSA8LSBzZWxiYWwuY3YoeD1iYWN0X2dlbnVzX3N1bV90LCB5PXZhcl9iYWN0LCB6ZXJvLnJlcD0ib25lIixzZWVkID0gMSxjb2wgPSBjKCIjMDA3MkIyIiwiI0Q1NUUwMCIpKQ0KY3ZfYmFjdF9QRF9BJGFjY3VyYWN5Lm52YXINCmN2X2JhY3RfUERfQSR2YXIuYmFycGxvdA0KZ3JpZC5kcmF3KGN2X2JhY3RfUERfQSRnbG9iYWwucGxvdCkNCnBsb3QudGFiKGN2X2JhY3RfUERfQSRjdi50YWIpDQpjdl9iYWN0X1BEX0EkZ2xtDQoNCiNtYWtlIHByZXR0eSBzZWxiYWwgIGN2Xw0KcG5nKCcuL2ZpbGVzL1NIVF9zZWxiYWwvMjAyMC0wOS0yMF9jYl9zZWxiYWxfY3ZfYmFjdF9QRF9BLnBuZycsIHdpZHRoPTI1MDAsIGhlaWdodD0yNTAwLCByZXM9NDAwKQ0KZ3JpZC5kcmF3KGN2X2JhY3RfUERfQSRnbG9iYWwucGxvdCkNCmRldi5vZmYoKQ0KYGBgDQoNCmBgYHtyfQ0KI21ldGFiIHNlbGJhbCBBIEMNCm1ldGFiX21hcF9ub1BhX0FDPC0gbWV0YWJfbWFwX25vUGFfQUNbZ3JlcCgnQXxDJyxtZXRhYl9tYXBfbm9QYV9BQyRkaXNlYXNjbGFzcyksXQ0KbWV0YWJfbm9QYV9BQyA8LSBtZXRhYl9ub1BhX0FDWyxnc3ViKCcuQicsJ0InLHJvd25hbWVzKG1ldGFiX21hcF9ub1BhX0FDKSldDQoNCnZhcl9tZXRhYiA8LSBhcy5mYWN0b3IobWV0YWJfbWFwX25vUGFfQUMkZGlzZWFzY2xhc3MpDQoNCiNzZWxiYWwgbmVlZHMgdHdvIGlucHV0cy0gbWF0cml4IHdpdGggc2FtcGxlcyBhcyByb3dzIGFuZCB0YXhhIGFzIGNvbHVtbg0KI3NldHVwIA0KbWV0YWJfZ2VudXNfc3VtX3QgPC0gdChtZXRhYl9ub1BhX0FDKSMNCg0KI3NlbGJhbCBwcmUgcmVzcA0KY3ZfbWV0YWJfQXZDX0RDIDwtIHNlbGJhbC5jdih4PW1ldGFiX2dlbnVzX3N1bV90LCB5PXZhcl9tZXRhYiwgemVyby5yZXA9ImJheWVzIixzZWVkID0gMSxjb2wgPSBjKCIjMDA3MkIyIiwiI0Q1NUUwMCIpKQ0KY3ZfbWV0YWJfQXZDX0RDJGFjY3VyYWN5Lm52YXINCmN2X21ldGFiX0F2Q19EQyR2YXIuYmFycGxvdA0KZ3JpZC5kcmF3KGN2X21ldGFiX0F2Q19EQyRnbG9iYWwucGxvdCkNCnBsb3QudGFiKGN2X21ldGFiX0F2Q19EQyRjdi50YWIpDQpjdl9tZXRhYl9BdkNfREMkZ2xtDQoNCiNtYWtlIHByZXR0eSBzZWxiYWwgIGN2Xw0KcG5nKCcuL2ZpbGVzL1NIVF9zZWxiYWwvMjAyMC0wOS0yMF9jYl9zZWxiYWxfY3ZfbWV0YWJfRENfQUMucG5nJywgd2lkdGg9NTAwMCwgaGVpZ2h0PTI5MDAsIHJlcz00MDApDQpncmlkLmRyYXcoY3ZfbWV0YWJfQXZDX0RDJGdsb2JhbC5wbG90KQ0KZGV2Lm9mZigpDQoNCnBuZygnLi9maWxlcy9TSFRfc2VsYmFsLzIwMjAtMDktMjBfYl9jYl9zZWxiYWxfY3ZfbWV0YWJfRENfQUMucG5nJywgd2lkdGg9MjUwMCwgaGVpZ2h0PTI1MDAsIHJlcz00MDApDQpncmlkLmRyYXcoY3ZfbWV0YWJfQXZDX0RDJGdsb2JhbC5wbG90KQ0KZGV2Lm9mZigpDQpgYGANCg0KYGBge3J9DQojbWV0YWIgc2VsYmFsIEEgUEQNCm1ldGFiX21hcF9ub1BhX0FfUEQ8LSBtZXRhYl9tYXBfbm9QYV9BQ1tncmVwKCdBJyxtZXRhYl9tYXBfbm9QYV9BQyRkaXNlYXNjbGFzcyksXQ0KbWV0YWJfbm9QYV9BIDwtIG1ldGFiX25vUGFfQUNbLGdzdWIoJy5CJywnQicscm93bmFtZXMobWV0YWJfbWFwX25vUGFfQV9QRCkpXQ0KDQp2YXJfbWV0YWIgPC0gYXMubnVtZXJpYyhtZXRhYl9tYXBfbm9QYV9BX1BEJHBvY2tldGRlcHRoKQ0KbWV0YWJfbm9QYV9BIDwtIG1ldGFiX25vUGFfQVssIWlzLm5hKHZhcl9tZXRhYildDQp2YXJfbWV0YWIgPC0gdmFyX21ldGFiWyFpcy5uYSh2YXJfbWV0YWIpXQ0KI3NlbGJhbCBuZWVkcyB0d28gaW5wdXRzLSBtYXRyaXggd2l0aCBzYW1wbGVzIGFzIHJvd3MgYW5kIHRheGEgYXMgY29sdW1uDQojc2V0dXAgDQptZXRhYl9nZW51c19zdW1fdCA8LSB0KG1ldGFiX25vUGFfQSkjDQoNCiNzZWxiYWwgcHJlIHJlc3ANCmN2X21ldGFiX0FfUEQgPC0gc2VsYmFsLmN2KHg9bWV0YWJfZ2VudXNfc3VtX3QsIHk9dmFyX21ldGFiLCB6ZXJvLnJlcD0iYmF5ZXMiLHNlZWQgPSAxLGNvbCA9IGMoIiMwMDcyQjIiLCIjRDU1RTAwIikpDQpjdl9tZXRhYl9BX1BEJGFjY3VyYWN5Lm52YXINCmN2X21ldGFiX0FfUEQkdmFyLmJhcnBsb3QNCmdyaWQuZHJhdyhjdl9tZXRhYl9BX1BEJGdsb2JhbC5wbG90KQ0KcGxvdC50YWIoY3ZfbWV0YWJfQV9QRCRjdi50YWIpDQpjdl9tZXRhYl9BX1BEJGdsbQ0KDQojbWFrZSBwcmV0dHkgc2VsYmFsICBjdl8NCnBuZygnLi9maWxlcy9TSFRfc2VsYmFsLzIwMjAtMDktMjBfY2Jfc2VsYmFsX2N2X21ldGFiX1BEX0EucG5nJywgd2lkdGg9MjUwMCwgaGVpZ2h0PTI1MDAsIHJlcz00MDApDQpncmlkLmRyYXcoY3ZfbWV0YWJfQV9QRCRnbG9iYWwucGxvdCkNCmRldi5vZmYoKQ0KYGBgDQoNCmBgYHtyfQ0KI21ldGFiIHNlbGJhbCBDIFBEDQptZXRhYl9tYXBfbm9QYV9DX1BEPC0gbWV0YWJfbWFwX25vUGFfQUNbZ3JlcCgnQycsbWV0YWJfbWFwX25vUGFfQUMkZGlzZWFzY2xhc3MpLF0NCm1ldGFiX25vUGFfQyA8LSBtZXRhYl9ub1BhX0FDWyxnc3ViKCcuQicsJ0InLHJvd25hbWVzKG1ldGFiX21hcF9ub1BhX0NfUEQpKV0NCg0KI3NlbGJhbCBuZWVkcyB0d28gaW5wdXRzLSBtYXRyaXggd2l0aCBzYW1wbGVzIGFzIHJvd3MgYW5kIHRheGEgYXMgY29sdW1uDQojc2V0dXAgDQp2YXJfbWV0YWIgPC0gYXMubnVtZXJpYyhtZXRhYl9tYXBfbm9QYV9DX1BEJHBvY2tldGRlcHRoKQ0KbWV0YWJfbm9QYV9DIDwtIG1ldGFiX25vUGFfQ1ssIWlzLm5hKHZhcl9tZXRhYildDQp2YXJfbWV0YWIgPC0gdmFyX21ldGFiWyFpcy5uYSh2YXJfbWV0YWIpXQ0KbWV0YWJfZ2VudXNfc3VtX3QgPC0gdChtZXRhYl9ub1BhX0MpIw0KDQojc2VsYmFsIHByZSByZXNwDQpjdl9tZXRhYl9DX1BEIDwtIHNlbGJhbC5jdih4PW1ldGFiX2dlbnVzX3N1bV90LCB5PXZhcl9tZXRhYiwgemVyby5yZXA9ImJheWVzIixzZWVkID0gMSxjb2wgPSBjKCIjMDA3MkIyIiwiI0Q1NUUwMCIpKQ0KY3ZfbWV0YWJfQ19QRCRhY2N1cmFjeS5udmFyDQpjdl9tZXRhYl9DX1BEJHZhci5iYXJwbG90DQpncmlkLmRyYXcoY3ZfbWV0YWJfQ19QRCRnbG9iYWwucGxvdCkNCnBsb3QudGFiKGN2X21ldGFiX0NfUEQkY3YudGFiKQ0KY3ZfbWV0YWJfQ19QRCRnbG0NCg0KI21ha2UgcHJldHR5IHNlbGJhbCAgY3ZfDQpwbmcoJy4vZmlsZXMvU0hUX3NlbGJhbC8yMDIwLTA5LTIwX2NiX3NlbGJhbF9jdl9tZXRhYl9QRF9DLnBuZycsIHdpZHRoPTI1MDAsIGhlaWdodD0yNTAwLCByZXM9NDAwKQ0KZ3JpZC5kcmF3KGN2X21ldGFiX0NfUEQkZ2xvYmFsLnBsb3QpDQpkZXYub2ZmKCkNCmBgYA0KDQpgYGB7cn0NCiNtZXRhZ2VuIHNlbGJhbCBBIEMNCnRhYmxlKG1ldGFnZW5fbm9QYT4xMCkNCm1ldGFnZW5fbWFwX25vUGFfQUMgIw0KbWV0YWdlbl9kZl9ub1BhX0FDIDwtbWV0YWdlbl9ub1BhWyxyb3duYW1lcyhtZXRhZ2VuX21hcF9ub1BhX0FDKV0NCnJvd25hbWVzKG1ldGFnZW5fZGZfbm9QYV9BQykgPC1nc3ViKCcuKmdfXycsJycscm93bmFtZXMobWV0YWdlbl9kZl9ub1BhX0FDKSkNCm1ldGFnZW5fZGZfbm9QYV9BQw0KdmFyX21ldGFnZW4gPC0gYXMuZmFjdG9yKG1ldGFnZW5fbWFwX25vUGFfQUMkRGlzZWFzQ2xhc3MpDQoNCiNzZWxiYWwgbmVlZHMgdHdvIGlucHV0cy0gbWF0cml4IHdpdGggc2FtcGxlcyBhcyByb3dzIGFuZCB0YXhhIGFzIGNvbHVtbg0KI3NldHVwIA0KbWV0YWdlbl9nZW51c19zdW1fdCA8LSB0KG1ldGFnZW5fZGZfbm9QYV9BQykjDQp0YWJsZShtZXRhZ2VuX2dlbnVzX3N1bV90ID09MCkNCiNzZWxiYWwgcHJlIHJlc3ANCmN2X21ldGFnZW5fQXZDX0RDIDwtIHNlbGJhbC5jdih4PW1ldGFnZW5fZ2VudXNfc3VtX3QsIHk9dmFyX21ldGFnZW4sIHplcm8ucmVwPSJiYXllcyIsc2VlZCA9IDEsY29sID0gYygiIzAwNzJCMiIsIiNENTVFMDAiKSkNCmN2X21ldGFnZW5fQXZDX0RDJGFjY3VyYWN5Lm52YXINCmN2X21ldGFnZW5fQXZDX0RDJHZhci5iYXJwbG90DQpncmlkLmRyYXcoY3ZfbWV0YWdlbl9BdkNfREMkZ2xvYmFsLnBsb3QpDQpwbG90LnRhYihjdl9tZXRhZ2VuX0F2Q19EQyRjdi50YWIpDQpjdl9tZXRhZ2VuX0F2Q19EQyRnbG0NCg0KI21ha2UgcHJldHR5IHNlbGJhbCAgY3ZfDQpwbmcoJy4vZmlsZXMvU0hUX3NlbGJhbC8yMDIwLTA5LTIwX2NiX3NlbGJhbF9jdl9tZXRhZ2VuX0RDX0FDLnBuZycsIHdpZHRoPTUwMDAsIGhlaWdodD0yOTAwLCByZXM9NDAwKQ0KZ3JpZC5kcmF3KGN2X21ldGFnZW5fQXZDX0RDJGdsb2JhbC5wbG90KQ0KZGV2Lm9mZigpDQoNCnBuZygnLi9maWxlcy9TSFRfc2VsYmFsLzIwMjAtMDktMjBfYl9jYl9zZWxiYWxfY3ZfbWV0YWdlbl9EQ19BQy5wbmcnLCB3aWR0aD0yNTAwLCBoZWlnaHQ9MjUwMCwgcmVzPTQwMCkNCmdyaWQuZHJhdyhjdl9tZXRhZ2VuX0F2Q19EQyRnbG9iYWwucGxvdCkNCmRldi5vZmYoKQ0KYGBgDQoNCmBgYHtyfQ0KDQoNCiNtZXRhZ2VuIHNlbGJhbCBBIFBEDQptZXRhZ2VuX21hcF9ub1BhX0FDDQoNCnRhYmxlKG1ldGFnZW5fbm9QYT4xMCkNCm1ldGFnZW5fbWFwX25vUGFfQSA8LSBtZXRhZ2VuX21hcF9ub1BhX0FDW2dyZXAoJ0EnLG1ldGFnZW5fbWFwX25vUGFfQUMkRGlzZWFzQ2xhc3MpLF0NCm1ldGFnZW5fZGZfbm9QYV9BIDwtbWV0YWdlbl9ub1BhWyxyb3duYW1lcyhtZXRhZ2VuX21hcF9ub1BhX0EpXQ0Kcm93bmFtZXMobWV0YWdlbl9kZl9ub1BhX0EpIDwtZ3N1YignLipnX18nLCcnLHJvd25hbWVzKG1ldGFnZW5fZGZfbm9QYV9BKSkNCm1ldGFnZW5fZGZfbm9QYV9BDQp2YXJfbWV0YWdlbiA8LSBhcy5udW1lcmljKG1ldGFnZW5fbWFwX25vUGFfQSRQb2NrZXREZXB0aCkNCg0KI3NlbGJhbCBuZWVkcyB0d28gaW5wdXRzLSBtYXRyaXggd2l0aCBzYW1wbGVzIGFzIHJvd3MgYW5kIHRheGEgYXMgY29sdW1uDQojc2V0dXAgDQptZXRhZ2VuX2dlbnVzX3N1bV90IDwtIHQobWV0YWdlbl9kZl9ub1BhX0EpIw0KdGFibGUobWV0YWdlbl9nZW51c19zdW1fdCA9PTApDQojc2VsYmFsIHByZSByZXNwDQpjdl9tZXRhZ2VuX0FfUEQgPC0gc2VsYmFsLmN2KHg9bWV0YWdlbl9nZW51c19zdW1fdCwgeT12YXJfbWV0YWdlbiwgemVyby5yZXA9ImJheWVzIixzZWVkID0gMSxjb2wgPSBjKCIjMDA3MkIyIiwiI0Q1NUUwMCIpKQ0KY3ZfbWV0YWdlbl9BX1BEJGFjY3VyYWN5Lm52YXINCmN2X21ldGFnZW5fQV9QRCR2YXIuYmFycGxvdA0KZ3JpZC5kcmF3KGN2X21ldGFnZW5fQV9QRCRnbG9iYWwucGxvdCkNCnBsb3QudGFiKGN2X21ldGFnZW5fQV9QRCRjdi50YWIpDQpjdl9tZXRhZ2VuX0FfUEQkZ2xtDQoNCiNtYWtlIHByZXR0eSBzZWxiYWwgIGN2Xw0KcG5nKCcuL2ZpbGVzL1NIVF9zZWxiYWwvMjAyMC0wOS0yMF9zZWxiYWxfY3ZfbWV0YWdlbl9QRF9BLnBuZycsIHdpZHRoPTI1MDAsIGhlaWdodD0yNTAwLCByZXM9NDAwKQ0KZ3JpZC5kcmF3KGN2X21ldGFnZW5fQV9QRCRnbG9iYWwucGxvdCkNCmRldi5vZmYoKQ0KYGBgDQoNCmBgYHtyfQ0KI21ldGFnZW4gc2VsYmFsIEFDIFBEDQptZXRhZ2VuX21hcF9ub1BhDQoNCnRhYmxlKG1ldGFnZW5fbm9QYT4xMCkNCm1ldGFnZW5fbWFwX25vUGFfQUMgPC0gbWV0YWdlbl9tYXBfbm9QYV9BQ1tncmVwKCdBfEMnLG1ldGFnZW5fbWFwX25vUGFfQUMkRGlzZWFzQ2xhc3MpLF0NCm1ldGFnZW5fZGZfbm9QYV9BQyA8LW1ldGFnZW5fbm9QYVsscm93bmFtZXMobWV0YWdlbl9tYXBfbm9QYV9BQyldDQpyb3duYW1lcyhtZXRhZ2VuX2RmX25vUGFfQUMpIDwtZ3N1YignLipnX18nLCcnLHJvd25hbWVzKG1ldGFnZW5fZGZfbm9QYV9BQykpDQptZXRhZ2VuX2RmX25vUGFfQUMNCnZhcl9tZXRhZ2VuIDwtIGFzLm51bWVyaWMobWV0YWdlbl9tYXBfbm9QYV9BQyRQb2NrZXREZXB0aCkNCg0KI3NlbGJhbCBuZWVkcyB0d28gaW5wdXRzLSBtYXRyaXggd2l0aCBzYW1wbGVzIGFzIHJvd3MgYW5kIHRheGEgYXMgY29sdW1uDQojc2V0dXAgDQptZXRhZ2VuX2dlbnVzX3N1bV90IDwtIHQobWV0YWdlbl9kZl9ub1BhX0FDKSMNCnRhYmxlKG1ldGFnZW5fZ2VudXNfc3VtX3QgPT0wKQ0KI3NlbGJhbCBwcmUgcmVzcA0KY3ZfbWV0YWdlbl9BQ19QRCA8LSBzZWxiYWwuY3YoeD1tZXRhZ2VuX2dlbnVzX3N1bV90LCB5PXZhcl9tZXRhZ2VuLCB6ZXJvLnJlcD0iYmF5ZXMiLHNlZWQgPSAxLGNvbCA9IGMoIiMwMDcyQjIiLCIjRDU1RTAwIikpDQpjdl9tZXRhZ2VuX0FDX1BEJGFjY3VyYWN5Lm52YXINCmN2X21ldGFnZW5fQUNfUEQkdmFyLmJhcnBsb3QNCmdyaWQuZHJhdyhjdl9tZXRhZ2VuX0FDX1BEJGdsb2JhbC5wbG90KQ0KcGxvdC50YWIoY3ZfbWV0YWdlbl9BQ19QRCRjdi50YWIpDQpjdl9tZXRhZ2VuX0FDX1BEJGdsbQ0KDQojbWFrZSBwcmV0dHkgc2VsYmFsICBjdl8NCnBuZygnLi9maWxlcy9TSFRfc2VsYmFsLzIwMjAtMDktMjBfc2VsYmFsX2N2X21ldGFnZW5fUERfQUMucG5nJywgd2lkdGg9MjUwMCwgaGVpZ2h0PTI1MDAsIHJlcz00MDApDQpncmlkLmRyYXcoY3ZfbWV0YWdlbl9BQ19QRCRnbG9iYWwucGxvdCkNCmRldi5vZmYoKQ0KYGBgDQoNCmBgYHtyfQ0KI21ldGFnZW4gc2VsYmFsIEMgUEQNCm1ldGFnZW5fbWFwX25vUGFfQUMNCg0KdGFibGUobWV0YWdlbl9ub1BhPjEwKQ0KbWV0YWdlbl9tYXBfbm9QYV9DIDwtIG1ldGFnZW5fbWFwX25vUGFfQUNbZ3JlcCgnQycsbWV0YWdlbl9tYXBfbm9QYV9BQyREaXNlYXNDbGFzcyksXQ0KbWV0YWdlbl9kZl9ub1BhX0MgPC1tZXRhZ2VuX25vUGFbLHJvd25hbWVzKG1ldGFnZW5fbWFwX25vUGFfQyldDQpyb3duYW1lcyhtZXRhZ2VuX2RmX25vUGFfQykgPC1nc3ViKCcuKmdfXycsJycscm93bmFtZXMobWV0YWdlbl9kZl9ub1BhX0MpKQ0KbWV0YWdlbl9kZl9ub1BhX0MNCnZhcl9tZXRhZ2VuIDwtIGFzLm51bWVyaWMobWV0YWdlbl9tYXBfbm9QYV9DJFBvY2tldERlcHRoKQ0KDQojc2VsYmFsIG5lZWRzIHR3byBpbnB1dHMtIG1hdHJpeCB3aXRoIHNhbXBsZXMgYXMgcm93cyBhbmQgdGF4YSBhcyBjb2x1bW4NCiNzZXR1cCANCm1ldGFnZW5fZ2VudXNfc3VtX3QgPC0gdChtZXRhZ2VuX2RmX25vUGFfQykjDQp0YWJsZShtZXRhZ2VuX2dlbnVzX3N1bV90ID09MCkNCiNzZWxiYWwgcHJlIHJlc3ANCmN2X21ldGFnZW5fQ19QRCA8LSBzZWxiYWwuY3YoeD1tZXRhZ2VuX2dlbnVzX3N1bV90LCB5PXZhcl9tZXRhZ2VuLCB6ZXJvLnJlcD0iYmF5ZXMiLHNlZWQgPSAxLGNvbCA9IGMoIiMwMDcyQjIiLCIjRDU1RTAwIikpDQpjdl9tZXRhZ2VuX0NfUEQkYWNjdXJhY3kubnZhcg0KY3ZfbWV0YWdlbl9DX1BEJHZhci5iYXJwbG90DQpjdl9tZXRhZ2VuX0NfUEQkZ2xtDQoNCiNtYWtlIHByZXR0eSBzZWxiYWwgIGN2Xw0KcG5nKCcuL2ZpbGVzL1NIVF9zZWxiYWwvMjAyMC0wOS0yMF9zZWxiYWxfY3ZfbWV0YWdlbl9QRF9DLnBuZycsIHdpZHRoPTI1MDAsIGhlaWdodD0yNTAwLCByZXM9NDAwKQ0KZ3JpZC5kcmF3KGN2X21ldGFnZW5fQ19QRCRnbG9iYWwucGxvdCkNCmRldi5vZmYoKQ0KYGBgDQo=
